# Helicalization of Covalent Organic Framework Nanofibers with Amplified Spin Polarizability for Boosting Photocatalytic Hydrogen Evolution

**DOI:** 10.1002/advs.75127

**Published:** 2026-04-03

**Authors:** Qi Zhong, Yongtu Tian, Yutao Sang, Changchun Wang, Jia Guo

**Affiliations:** ^1^ State Key Laboratory of Molecular Engineering of Polymers Department of Macromolecular Science Fudan University Shanghai P. R. China

**Keywords:** chiral‐induced spin selectivity, covalent organic frameworks, helical chirality, photocatalysis hydrogen evolution

## Abstract

Given that chirality can induce spin polarization via the chirality‐induced spin selectivity (CISS) effect, integrating conformational chirality into covalent organic frameworks (COFs) can offer a route to harness CISS for long‐range spin‐polarized charge transfer in crystalline materials. However, achieving both high crystallinity and strong chirality in COFs remains challenging. Here, we report a noncovalent chirality transfer strategy to modulate helical assembly of 2D COF nanofibers from achiral building blocks. The chiral solvent (*R/S*)‐3‐chloro‐1,2‐propanediol triggers a stacking deviation of bipyridine modules via hydrogen bonding and halogen bonding, leading to chirality transfer and steering helical growth. The resulting COF features global conformational chirality, helically intertwined morphology and high crystallinity. In photocatalytic hydrogen evolution, the helical COF achieves a H_2_ production rate of 44 mmol g^−1^ h^−1^ with the quantum efficiency of 7.18% at 500 nm, markedly outperforming achiral and weakly chiral analogues. The superior performance originates from the helical structure, which induces exceptional spin polarization (74%‐88%) via CISS, thereby enhancing exciton dissociation and extending carrier lifetime. This work establishes a strategy of noncovalently directed helical crystallization of 2D COFs and demonstrates that rendering organic semiconductors helicity can amplify spin polarization to enhance intrinsic photocatalytic activity, offering a design pathway for advanced photocatalysts.

## Graphical abstract

A non‐covalent chirality‐induced strategy via chiral solvents is presented to synthesize helical 2D COF nanofibers with high crystallinity, high chirality, and high spin polarization. The helical structure enhances the concentration of photogenerated charges, suppresses exciton recombination, and extends carrier lifetimes, thereby significantly improving photocatalytic hydrogen production performance, which is twice that of the achiral analog.

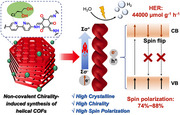

## 1 Introduction

For organic semiconductors, a significant challenge in photocatalysis lies in the requirement for photogenerated charges to migrate over long distances to the surface or interface to participate in redox reactions [1]. Despite this challenge, they offer distinct advantages over inorganic counterparts. Through rational molecular design, they enable broad‐spectrum light absorption and allow for flexible regulation of electronic properties via donor‐acceptor architectures [2, 3]. However, their intrinsically low dielectric properties severely restrict long‐range charge carrier mobility and promote charge recombination during migration. Consequently, only charges generated near the surface contribute significantly to photocatalysis, leaving the majority of bulk‐generated charges underutilized [4]. Therefore, enhancing long‐range electron transport to leverage bulk‐generated charges poses a critical challenge in advancing organic photocatalysts.

**SCHEME 1:**
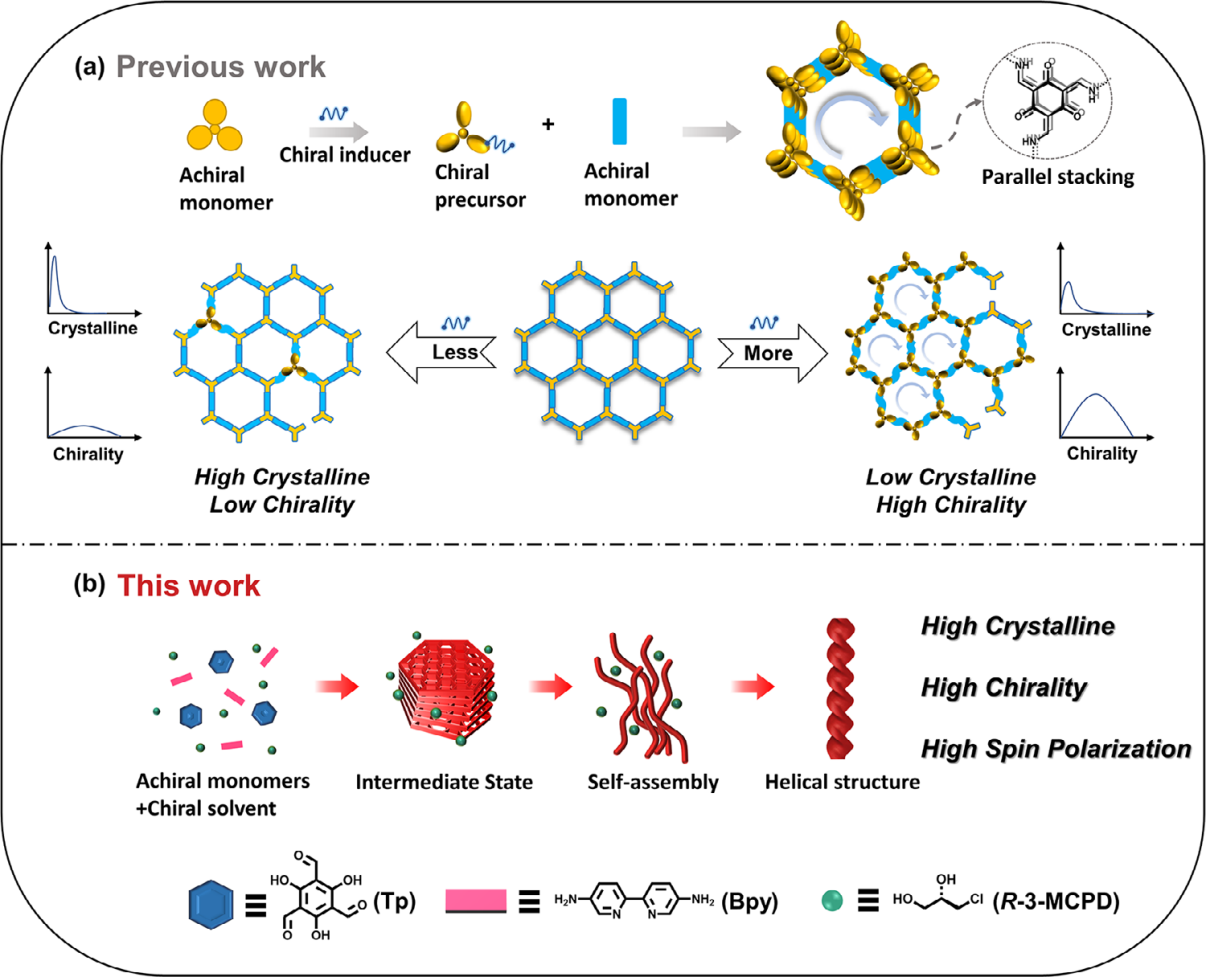
(a) Illustration of the reported chiral induction strategy for 2D COFs with impaired crystallinity or chirality. (b) Proposed noncovalent chiral‐solvent‐induced strategy for the synthesis of helical 2D COF.

Covalent organic frameworks (COFs) represent an emerging class of porous crystalline materials consisting of organic building blocks alternatively connected via covalent bonds. Their predictable crystalline architectures with defined topological motifs allow for the bottom‐up or post‐synthetic integration of multiple components such as photosensitizers [5, 6] donor‐acceptor pairs [7, 8, 9], and chiral modules [10, 11, 12, 13] into COF frameworks. Specifically, 2D COFs with periodic atomic arrangement facilitate eclipsed stacking one on top of the other, creating extended pathways conducive to long‐range charge carrier mobility [14]. As demonstrated in our recent work, chirality‐induced synthesis of 2D COFs enables stereo control over the orientation of covalent linkages, thereby minimizing structural misalignment and achieving well‐ordered parallel layer stacking [15, 16]. In contrast, bottom‐up incorporation of external chiral centers into the frameworks fails to restrict bond rotation in linear linkages such as imine and vinylene bonds [17, 18]. Given that the globally conformational chirality of 2D COFs allows for the superimposed arrangement between neighboring layers, the transfer dynamics and concentrations of photogenerated electrons are significantly enhanced, outperforming achiral analogues [19]. It seems to address the persistent challenge of photogenerated charge transfer in bulk phases. Nevertheless, the utilization of a large number of chiral inducers such as (*R/S*)‐1‐phenylethylamine with monofunctional groups largely suppresses lateral growth of planar frameworks, impairing crystallinity and in turn impeding charge mobility due to increased defective sites (Scheme 1a). Therefore, a key challenge remains in simultaneously achieving high crystallinity and strong chirality in 2D COFs synthesized via the currently reported chirality‐induced approach.

In chiral materials, chirality‐induced spin selectivity effect (CISS) plays a crucial role in electron transmission with one spin orientation filtered by chiral configuration [20, 21]. CISS‐enhanced electronic dynamics have been explored in a few of inorganic systems such as BrOBi [22] and ZnO [23] for photocatalytic applications including CO_2_ reduction and O_2_ evolution. The helical structure has been incorporated into inorganic photocatalysts for amplifying their intrinsic chiroptical properties. Accordingly, it renders a promising strategy to enhance spin polarization and improve electron transfer efficiency in photocatalytic reactions. In contrast, the application of the CISS effect to enhance catalytic performances of chiral COFs primarily focuses on electrocatalytic oxygen evolution [24, 25]. Although transitioning from molecular chiral configuration to their helical structure has been well established, the effect of helical structures on the performances of organic photocatalysts remains largely unexplored.

Herein, we report a chiral solvent‐directed crystallization strategy for the helicalization of 2D COFs using achiral building blocks (Scheme 1b). The preparation of helical COFs have been reported by the bottom‐up synthesis using chiral monomers [26, 27, 28, 29], in situ growth on the helical templates [30, 31], and micelle‐assisted self‐assembly of achiral COFs [32, 33]. Unlike those methods, regulating the helical assembly of achiral COFs via chiral solvent is more challenging, owing to the poor uniformity in the mesoscopically oriented assembly of COF microcrystals. Using 3‐chloro‐1,2‐propanediol as a chiral solvent, we successfully achieve the helical assembly of bipyridine‐based 2D COF nanofibers. Furthermore, we demonstrate that adjusting the solvent ratio is crucial for the transfer and amplification of configurational chirality in 2D COFs. In photocatalytic tests, the helical COF remarkably outperforms the achiral and weakly chiral COFs in hydrogen evolution. Mechanistic studies reveal that the helical COF exhibits a high polarization value of 74%–88%, far exceeding that of the weakly chiral analog (15%‐33%), thereby promoting exciton dissociation and CISS‐mediated photoinduced electron transport. This work establishes a non‐covalent chirality‐induced crystallization methodology for 2D COFs, wherein the interaction between achiral building blocks and chiral solvents dictates the helical assembly of COF nanofibers. Meanwhile, we substantiate that helical organic photocatalysts benefit photogenerated electronic dynamics, illuminating a promising pathway for advanced photocatalysis.

## 2 Results and Discussion

We employed a solvothermal method to crystallize a 2D TpBpy‐COF, which consists of the achiral cyclohexane trione (Tp) as a knot and bipyridine (Bpy) as a strut. Unlike the reported strategy using reactive chiral inducers [34, 35, 36], we introduced a non‐covalent chirality transfer strategy to trigger the initial assembly of oligomers into an oriented stacking pattern early in the reaction. This was achieved by using (*R/S*)‐3‐chloro‐1,2‐propanediol (*R/S*‐3‐MCPD) as a chiral solvent, which interacts with electron‐rich N atoms on the *cis*‐form Bpy moieties through hydrogen bonding (N•••H) and halogen bonding (N•••Cl). These multiple non‐covalent interactions imprint chiral handedness into the repeating units, leading to the formation of oriented stacks in the crystal nuclei. According to earlier studies on the crystallization mechanism of 2D COFs [37, 38], such initial configurations can be propagated and maintained throughout the subsequent structural evolution, preserving both the molecular conformation and stacking mode in the layered COF without significant perturbation. Therefore, we propose that chiral transfer from (*R/S*)‐3‐MCPD to the COF structure likely occurs during the nucleation stage and is subsequently amplified through global crystal growth and self‐assembly into the resulting helical architecture.

**FIGURE 1:**
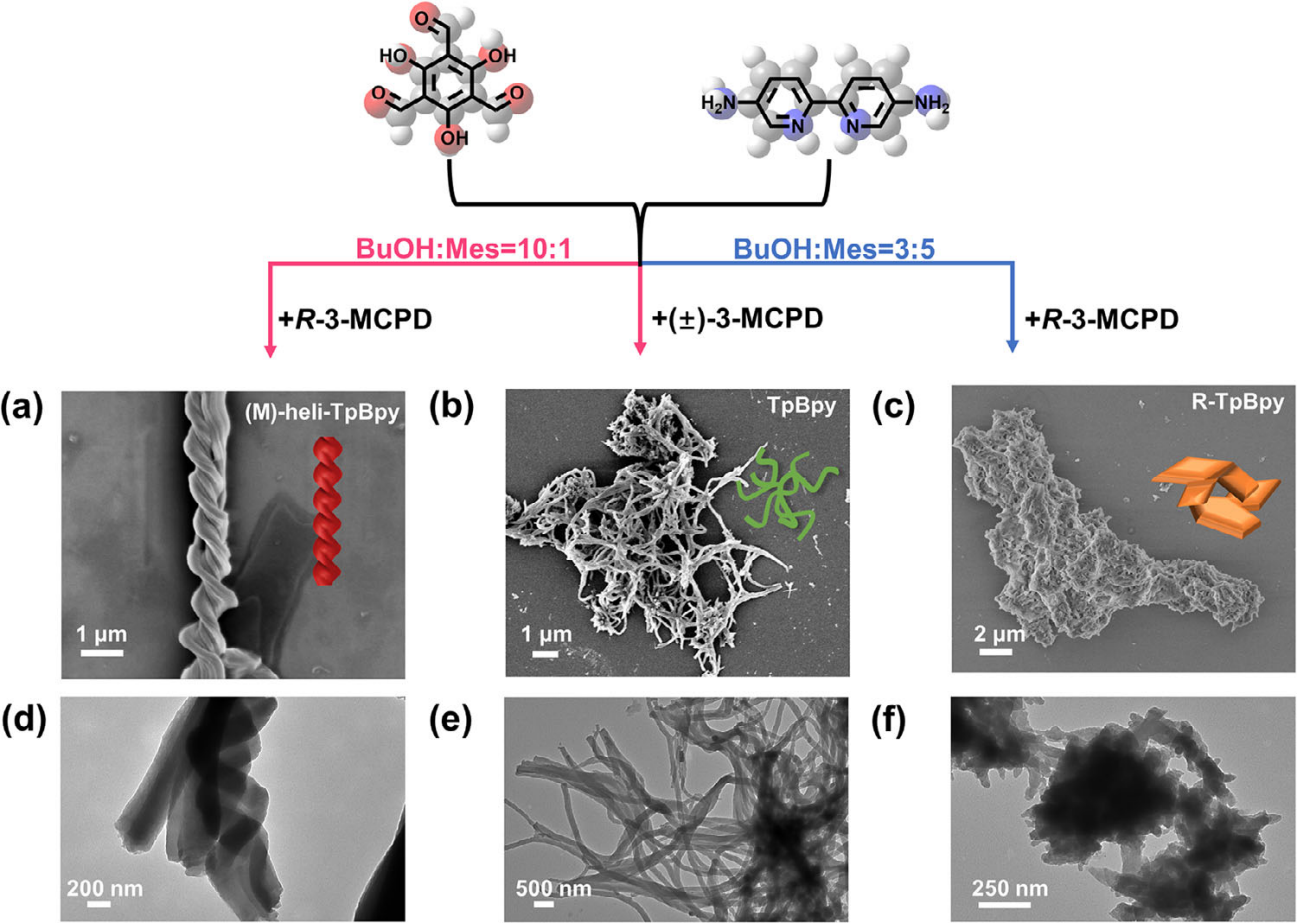
SEM and TEM images of (a,d) (*M*)‐heli‐TpBpy, (b,e) TpBpy, and (c,f) *R*‐TpBpy, which are synthesized using the different ratios of n‐butanol (BuOH) and mesitylene (Mes) (10:1 or 3:5, v/v) with *R*‐3‐MCPD or 3‐MCPD.

To validate this assumption, we synthesized TpBpy‐COF under solvothermal conditions (120°C, 3 days) using a solvent mixture of *n*‐butanol (BuOH), mesitylene (Mes) and (*R/S*)‐3‐MCPD, with 6 M HOAc as catalyst. In contrast to covalent chirality‐induced method, our non‐covalent approach allows the crystallinity, chirality, and helical morphology to be independently modulated by varying the solvent composition. As observed in Figure 1, when using a mixture of BuOH, Mes and (*R/S*)‐3‐MCPD in a volume ratio of 10:1:4, the COF assembled into multiple nanofibers intertwining into well‐defined helical structures with diameters around 850 nm and lengths ranging from 5 to 7 µm (Figure 1a,d; Figure S1). The helical orientation was directed by (*R/S*)‐3‐MCPD, yielding (*M*)‐ or (*P*)‐heli‐TpBpy COFs. In contrast, when achiral 3‐MCPD was used under the identical conditions, no helical structures formed (Figure 1b,e). Furthermore, by varying the solvent ratio of BuOH to Mes while maintaining the amount of (*R/S*)‐3‐MCPD, the product morphology was shifted from uniform nanofibers to irregular bulk aggregates (Figure 1c,f; Figure S2). These aggregates retained conformational chirality due to the induction of chiral solvents during the COF crystallization.

**FIGURE 2:**
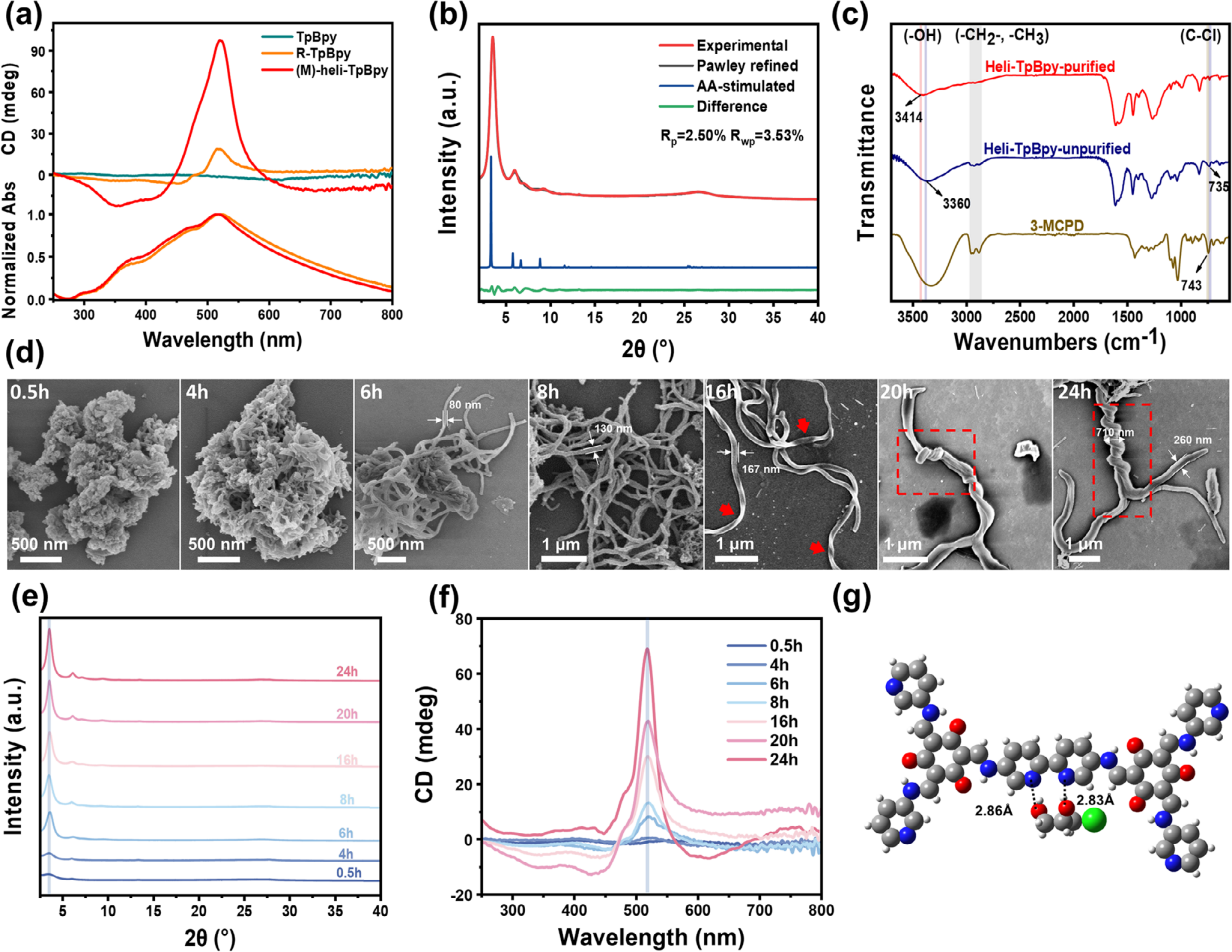
(a) CD and UV–vis spectra of TpBpy, *R*‐TpBpy and (*M*)‐heli‐TpBpy COFs. (b) PXRD pattern of (*M*)‐heli‐TpBpy (black) refined by the Pawley method (blue) with the difference from the observed pattern (green). (c) FT IR spectra of 3‐MCPD and helical TpBpy before and after purification. (d‐f) SEM images (d), PXRD patterns (e) and CD spectra (f) of (*M*)‐heli‐TpBpy obtained at different reaction times. (g) DFT optimization of the interaction between TpBpy moiety and (*R*)‐3‐MCPD.

Circular dichroism (CD) spectroscopy confirmed the global chirality of TpBpy‐COFs synthesized with chiral solvents. First, we verified that (*R/S*)‐3‐MCPD exhibited only a single CD peak at 200 nm in the UV region (Figure S3). Also, there was no CD signal in the TpBpy‐COF fibers synthesized with achiral 3‐MCPD. In contrast, both *R*‐TpBpy and (*M*)‐heli‐TpBpy displayed the positive Cotton effect around 500 nm, which well coincided with their absorption peaks (Figure 2a). The CD intensity of (*M*)‐heli‐TpBpy was much stronger than that of *R*‐TpBpy, which can be attributed to the higher degree of helical handedness in the morphology, thereby amplifying the chiral expression beyond molecular or structural chirality [27]. Correspondingly, *S*‐TpBpy and (*P*)‐heli‐TpBpy caused an opposite Cotton effect, resulting in nearly mirror‐image CD spectra relative to their *R*‐ and *M*‐counterparts (Figure S4).

To verify the crystalline structures of the obtained COFs in the chiral environment, we performed powder X‐ray diffraction (PXRD) analysis. As shown in Figure 2b, the (*M*)‐heli‐TpBpy COF exhibits a high‐quality diffraction pattern with X‐ray signals at 3.5°, 6.2°, and 26.8°, corresponding to the (100), (110) and (001) crystal facets, respectively. The experimental observation well aligned with those reported for TpBpy‐COF synthesized via a typical solvothermal route [39, 40]. As compared to the Pawley refined pattern, the helical COF still maintained an eclipsed‐AA stacking structure in the P6 space group, with lattice parameters a = b = 30.5293 Å, c = 3.4982 Å, and α = β = 90°, γ = 120° (R_p_ = 2.50%, R_wp_ = 3.53%). Besides, both the achiral TpBpy and the weakly chiral (*R/S*)‐TpBpy exhibited pronounced crystallinity (Figure S5). Moreover, their full width at half maxima (FWHM) of (100) peaks fell within 0.6–0.7°, indicating similar grain sizes (Table S1). In contrast, the use of a reactive chiral inducer led to significantly weakened X‐ray diffraction signals, implying that such monofunctional chiral inducer severely impeded the long‐range structural order of the chiral COF (Figure S6). These results further manifest the advantage of the non‐covalent chirality transfer strategy in constructing highly crystalline chiral COFs.

Nitrogen adsorption and desorption measurements at 77 K verified the permanent porosity of the chiral TpBpy COFs (Figure S7). All COFs exhibited typical type‐I sorption isotherms. The Brunauer–Emmett–Teller (BET) surface areas were accessed to be 705, 696, and 742 m^2^ g^−1^ for TpBpy, *R*‐TpBpy, and (*M*)‐heli‐TpBpy, respectively. Pore‐size distribution analysis based on the Non‐local Density Functional Theory (NLDFT) method revealed a dominant pore width of 2.2 nm for (*M*)‐heli‐TpBpy, signifying its microporous nature arising from ordered 1D pore channels (Figure S8). Additionally, FTIR spectra unambiguously validated the formation of all COFs, showing the characteristic stretching bands at 1611cm^−1^ for the C═O bond and at 1266 cm^−1^ for the C─N bond, which are attributed to β‐ketoenamine linkage (Figure S9) [41]. Thermogravimetric analysis (TGA) performed in air demonstrated that all materials retained thermal stability up to 350°C, with the framework decomposition gradually occurring above 400°C (Figure S10).

To manifest the interaction between 3‐MCPD and Bpy moiety, we compared the FTIR spectra of the helical TpBpy COF before and after Soxhlet extraction with THF. As depicted in Figure 2c, the as‐synthesized TpBpy facilitates the inclusion of (*R/S*)‐3‐MCPD within the main backbones, as evidenced by the presence of characteristic vibrations corresponding to saturated C─H bonds (3000–2700 cm^−1^) and C─Cl bonds (743 cm^−1^). There was a notable red shift in the C‐Cl vibration band from 735 to 743 cm^−1^, indicating the presence of halogen bonding between 3‐MCPD chlorine and Bpy nitrogen [42]. After purification, all characteristic bands associated with 3‐MCPD disappeared. Meanwhile, the O‐H stretching vibration shifted from 3360 to 3414 cm^−1^, which can be attributed to the dissociation of hydrogen bonding between the hydroxyl group of 3‐MCPD and TpBpy [43]. Also, X‐ray photoelectron spectroscopy (XPS) confirmed the absence of chlorine (Figure S11), corroborating that no (*R*)‐3‐MCPD remained on the surface or within the pore of (*M*)‐heli‐TpBpy.

The morphological evolution of (*M*)‐heli‐TpBpy during crystallization was investigated to clarify the helical originality. As exhibited in Figure 2d, the initial product obtained after 0.5 h reaction appears as irregular grains and develop into short rods within 4 h. As the reaction continuously proceeded, these nanorods further elongated into micrometer‐long flexible nanofibers with widths of ∼80 and ∼130 nm for the 6‐h and 8‐h products, respectively. Subsequently, we observed that the individual nanofibers began to twist by 16 h and several nanofibers twisted together to form a spiral‐shaped knot by 20 h. Then the entanglement of multiple nanofibers became more pronounced by 24 h, eventually assembling into well‐defined helical structures. Throughout this helical formation process, both the (100) domain sizes and the CD signals of TpBpy increased progressively (Figure 2e,f) and presented a nonlinear positive correlation (Figure S12), in which the chiral signature became increasingly pronounced after 8 h. Given that the initially formed solid exhibited neither helical morphology nor significant crystallinity or chirality, we infer that (*R/S*)‐3‐MCPD serves as an inducer that generates weak conformational chirality during the layered stacking of the chiral framework. This process originates from the interactions of hydrogen bonding and halogen bonding between (*R/S*)‐3‐MCPD and TpBpy. The subsequent helical assembly of nanofibers then amplifies the weak conformational chirality into spatially expressed helical chirality.

Taking all the findings into account, insightfully understanding the molecular mechanism for the chiral induction effect of (*R/S*)‐3‐MCPD is essential. We performed density function theory (DFT) calculation to seek for the possible atomic sites on TpBpy moiety that could engage in noncovalent interactions with (*R/S*)‐3‐MCPD. The computed outcomes demonstrated that the adjacent dual N atoms on the *cis*‐Bpy moiety represented the most favorable sites, interacting with the two hydroxyl groups of (*R/S*)‐3‐MCPD at distances of 2.83 and 2.86 Å, respectively (Figure 2g). Both values fall within the typical range of hydrogen bonding lengths. Further validation through electrostatic potential (ESP) mapping and noncovalent interaction (NCI) analysis confirmed that noncovalent interactions, mainly hydrogen bonding of the type N•••H‐O between Bpy moiety and (*R/S*)‐3‐MCPD, play a critical role in directing the chirality‐induced stacking of COF layers along the z‐axis (Figures S13 and S14).

Although theoretical calculations suggest a predominant role for hydrogen bonding between (*R/S*)‐3‐MCPD molecules and TpBpy units, we performed comparative experiments to further verify the indispensable contribution of halogen bonding in the chiral solvent‐induced helical assembly. Under identical synthetic conditions, (*R/S*)‐3‐MCPD was replaced with (*R/S*)‐1,2‐propanediol (*R/S*‐PD) to create the chiral environment (Figure S15). Although both (*R/S*)‐PD and (*R/S*)‐3‐MCPD exhibited similar CD signals with a peak around 200 nm in the UV region (Figure S16a), the CD signal of the chiral TpBpy synthesized using an equivalent amount of (*R/S*)‐PD was weaker (Figure S16b), and no macroscopic helical morphology was observed (Figure S17). These findings provide compelling evidence that the torsional force driving the helical twisting of COF nanofibers in chiral solvents arises from the synergistic interaction of both hydrogen bonding and halogen bonding.

Next, we investigated the effect of a mixed solvent system on the helical morphology and chirality. With an increase in the volume ratio of BuOH/Mes in the range from 20/1 to 40/1 under otherwise identical conditions, the width of the formed nanofibers gradually decreased and no helical morphology was observed (Figure S18). We infer that the induction effect of chiral solvents is severely impaired in the presence of excessive BuOH. This is presumably due to the competition between *R*‐3‐MCPD and BuOH in the noncovalent interaction with the COF frameworks. Thus, the low density of chiral inducers attached on the COF fibers is not enough to drive the helical assembly. Meanwhile, we varied the added amount of *R*‐3‐MCPD while keeping the optimal ratio of BuOH/Mes (10/1, v/v). When *R*‐3‐MCPD was added 50 and 100 µL, the material tended to form a single helical nanobelt with weak twisting degree. This is likely because the reduced proportion of *R*‐3‐MCPD weakens the chiral induction effect. When *R*‐3‐MCPD increased to 300 µL, the dense coiling of nanowires without distinct helical structure was observed, accompanied by a decrease in crystallinity and CD signal intensity (Figure S19). This indicates that the excessive *R*‐3‐MCPD disrupts the ordered stacking of the COF frameworks and their corresponding morphology evolution.

Prior to the photocatalytic test, we investigated the photophysical properties of (*P/M*)‐heli‐TpBpy compared to weakly chiral and achiral TpBpy COFs. UV–vis reflectance spectra revealed that all materials covered broad visible absorption, with absorption edges around 524 nm (Figure S20). The corresponding optical bandgaps, derived from Tauc plots, were determined to be 2.05 eV for achiral TpBpy and 2.10 eV for (*M*)‐heli‐TpBpy (Figure S21). As verified by the Mott‐Schottky plots, both materials behaved as n‐type semiconductors (Figure S22), with the flat‐band potentials of −0.93 and −0.98 V (vs. RHE) for achiral TpBpy and (*M*)‐heli‐TpBpy, respectively. As early reported, the flat‐band potential of n‐type semiconductors can be approximated as the conduction band (CB) minimum [44]. Based on the optical bandgap and CB values, the valence band (VB) positions were calculated to be 1.72 V (vs. RHE) for both achiral and helical TpBpy. Since the molecular‐level framework structure of all COFs remains consistent, the positions of the CB and VB were basically unchanged (Figure S23). Hence, (*M*)‐heli‐TpBpy remains thermodynamically capable of driving proton reduction.

**FIGURE 3:**
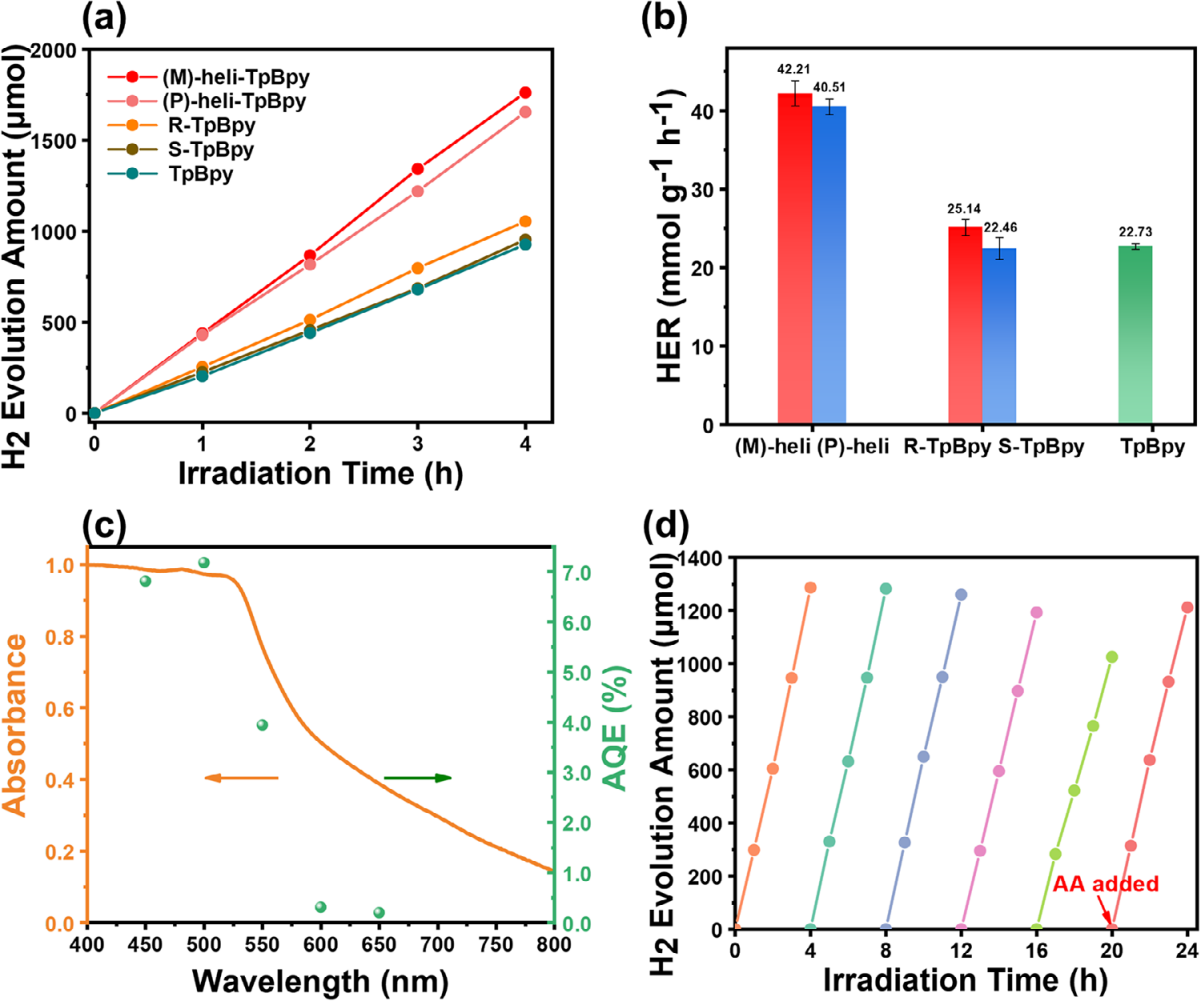
(a) Time course for photocatalytic H_2_ production under visible irradiation. (b) Comparing HERs of different COFs, the error bars are defined as standard deviation, and the center of each error bar represents the mean of three independent measurements (*n*  =  3). (c) Wavelength‐dependent AQE and (d) photocatalytic cycling test of (*M*)‐heli‐TpBpy.

The photocatalytic hydrogen evolution (PHE) experiments were performed in pure water under irradiation from a 300 W Xe‐lamp (λ > 420 nm) at 10°C, with ascorbic acid (AA, 0.1 M) serving as the sacrificial agent. Pt nanoparticles were photo‐deposited as a co‐catalyst onto the COFs and the optimum loading amount was determined to be 1wt% H_2_PtCl_6_ through the comparison of hydrogen evolution performance under identical conditions (Figure S24). Upon exposure to 4‐h visible irradiation at an average power density of 0.14 W cm^−2^ (Figure S25), all COFs provided a nearly linear increase in evolved H_2_ quantity over time (Figure 3a). The H_2_ evolution rate (HER) was calculated using 10 mg of catalysts. The helical COFs showed exceptionally high HER values, reaching 40.51 and 42.21 mmol g^−1^ h^−1^ for (*P*)‐ and (*M*)‐heli‐TpBpy, respectively, which were almost twice those of the weakly chiral and achiral COFs (Figure 3b). Based on the hourly hydrogen evolution amount (441 µmol h^−1^, 10 mg catalyst), (*M*)‐heli‐TpBpy can be ranked among the top of COF‐based photocatalysts (Figure S26 and Table S2).

The reproducibility of PHE performance was evidenced by the consistent HER values obtained from multiple batches of helical COFs synthesized under the same conditions (Figure S27). As illustrated in Figure 3c, the apparent quantum efficiency (AQE) aligns well with the corresponding absorption profile, signifying that photon absorption is directly associated with the photocatalytic conversion. The maximum AQE reaches 7.18% at 500 nm, likewise surpassing the values measured for weakly chiral and achiral TpBpy COFs.

To investigate the effect of the COF crystallization and helical formation processes on the photocatalytic performance, we evaluated heli‐TpBpy samples obtained from the different reaction times (0.5, 8, 16, 24, and 72 h). After 8 h, the dominant (100) peak intensity increased slowly while the corresponding chiral signals were dramatically enhanced (Figure S28). The HER of the 72‐h product (44.0 mmol g^−1^ h^−1^) was approximately 7.3 times higher than that of the 8‐h product (6.0 mmol g^−1^ h^−1^). This comparison indicates that the transformation from weakly chiral COF to helical COF is predominately attributed to the enhanced photocatalytic performance.

The long‐term photocatalytic stability of (*M*)‐heli‐TpBpy was validated by recycling tests, in which a consistent HER was maintained over multiple 4‐h irradiation cycles (Figure 3d). A decrease in H_2_ evolution was observed in the fifth cycle, attributable to the gradual consumption of the sacrificial agent AA. Upon replenishment of AA, the photocatalytic performance recovered to its original level, manifesting the exceptional stability of the helical TpBpy under photocatalytic conditions. In addition, the post‐photocatalysis characterizations confirmed that the helical morphology of (*M*)‐heli‐TpBpy was preserved while the crystallinity, porosity, and chiral signals were compromised to some extent as a result of a slight increase in disordered stacking of frameworks (Figure S29) [45]. The photo‐deposited Pt nanoparticles with a predominant size of ∼2 nm also remained a well‐dispersed distribution on the COF surface (Figure S30).

**FIGURE 4:**
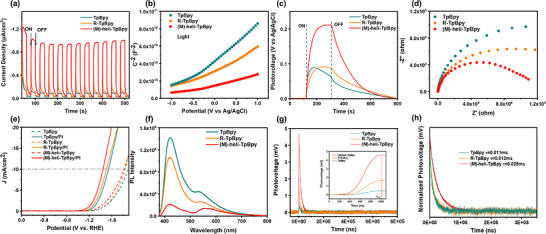
(a) Transient photocurrents responses. (b) Mott–Schottky plots under visible irradiation (λ > 420 nm). (c) Transient open‐circuit voltage decay profiles. (d) EIS spectra. (e) LSV plots for photo‐catalysts before and after Pt loading. (f) PL spectra. (g) Transient photovoltage curves. (h) Transient photovoltage decay curves with the single‐exponential fitting for calculating the decay lifetimes. The inset in (g) shows a magnified view in the range of 0–1100 ns.

Transient photocurrent measurement was carried out to investigate the photoinduced charge transport behavior. Under repeated on/off illumination cycles at 20‐s intervals, the helical TpBpy COF exhibited a significantly higher photocurrent intensity compared to the weakly chiral and achiral analogues (Figure 4a; Figure S31). Meanwhile, (*M*)‐heli‐TpBpy offered the approximately 10‐times higher photocurrent density compared to the weakly chiral and achiral analogues. Mott–Schottky plots obtained at the same frequency allowed for a much lower slope for helical TpBpy, indicating a higher carrier concentration relative to the other COFs (Figure 4b; Figure S32).

To probe the photogenerated carrier dynamics, transient open circuit voltage decay (OCVD) was performed under pulsed laser excitation. The helical TpBpy displayed a significantly higher open‐circuit voltage compared to its achiral and weakly chiral counterparts (Figure 4c; Figure S33), indicating a greater density of photogenerated carriers and an extended carrier lifetime. This enhancement can originate from markedly suppressed charge recombination within the helical structure. Electrochemical impedance spectra (EIS) reflected the increasing order of charge transfer resistance, followed by helical TpBpy, weakly chiral TpBpy and achiral TpBpy (Figure 4d; Figure S34). This trend is in a good agreement with the transient photocurrent result, proving that the helical TpBpy enables the generation of a greater number of charge carriers, thereby contributing to its superior conductivity. Upon immobilization of Pt nanoparticles onto the COFs, the electronic structure of Pt is modified due to multitude interactions between the attached Pt and the polarized moieties of the COF frameworks. Linear sweep voltammetry (LSV) was employed to monitor this change through the measurement of onset potentials for electrochemical reduction. We can see that (*M*)‐heli‐TpBpy allowed for the lowest overpotential of −1.44 V (vs. RHE) at a current density of 10 mA cm^−2^ after Pt deposition, indicative of a favorable reduction overpotential and enhanced catalytic activity of Pt in photocatalysis (Figure 4e; Figure S35).

Steady–state photoluminescence (PL) spectroscopy was utilized to investigate the exciton dynamics. As shown in Figure 4f and Figure S36, the dominant fluorescent emission is most strongly suppressed in the helical TpBpy over the 400–450 nm range, followed by weakly chiral TpBpy and finally achiral TpBpy. This manifests that the helical TpBpy facilitates electron‐hole dissociation in the excited state. The time‐resolved PL decay curves were fitted with a single‐exponential function, giving the exciton lifetimes of 3.00, 2.95, and 2.54 ns for (*M*)‐heli‐TpBpy, weakly chiral *R*‐TpBpy and achiral TpBpy, respectively (Figure S37). Also, the enantiomeric (*P*)‐heli‐TpBpy equally showed a longer exciton lifetime than *S*‐TpBpy (Figure S38). These findings support the assumption that the enhanced spin polarization effect in the helical TpBpy plays a critical role in prolonging carrier lifetimes and facilitating exciton dissociation, primarily presenting intrinsic origin of the photocatalytic mechanism.

Next, we carried out transient photovoltage (TPV) measurement to investigate photogenerated carrier transmission dynamics. As shown in Figure 4g, upon exposure to pulsed laser, (*M*)‐heli‐TpBpy exhibits the highest accumulation of holes on its surface, resulting in the greatest increase in surface photovoltage compared to *R*‐TpBpy and TpBpy. The charge extraction time (*t*_max_) of (*M*)‐heli‐TpBpy (1009 ns) was approximate to those of *R*‐TpBpy (1017 ns) and TpBpy (1021 ns), indicating their similar carrier transfer rates. By fitting the decay curve of the transient surface photocurrent, (*M*)‐heli‐TpBpy gave a carrier lifetime of 0.025 ms, which was almost twice those of *R*‐TpBpy (0.013 ms) and TpBpy (0.011 ms) (Figure 4h). Thus, we determine that the helical COF provides the enhanced polarizability for suppressing electron‐hole recombination, extending carrier lifetimes and accumulating more carriers at the material surface for photocatalytic hydrogen production.

**FIGURE 5:**
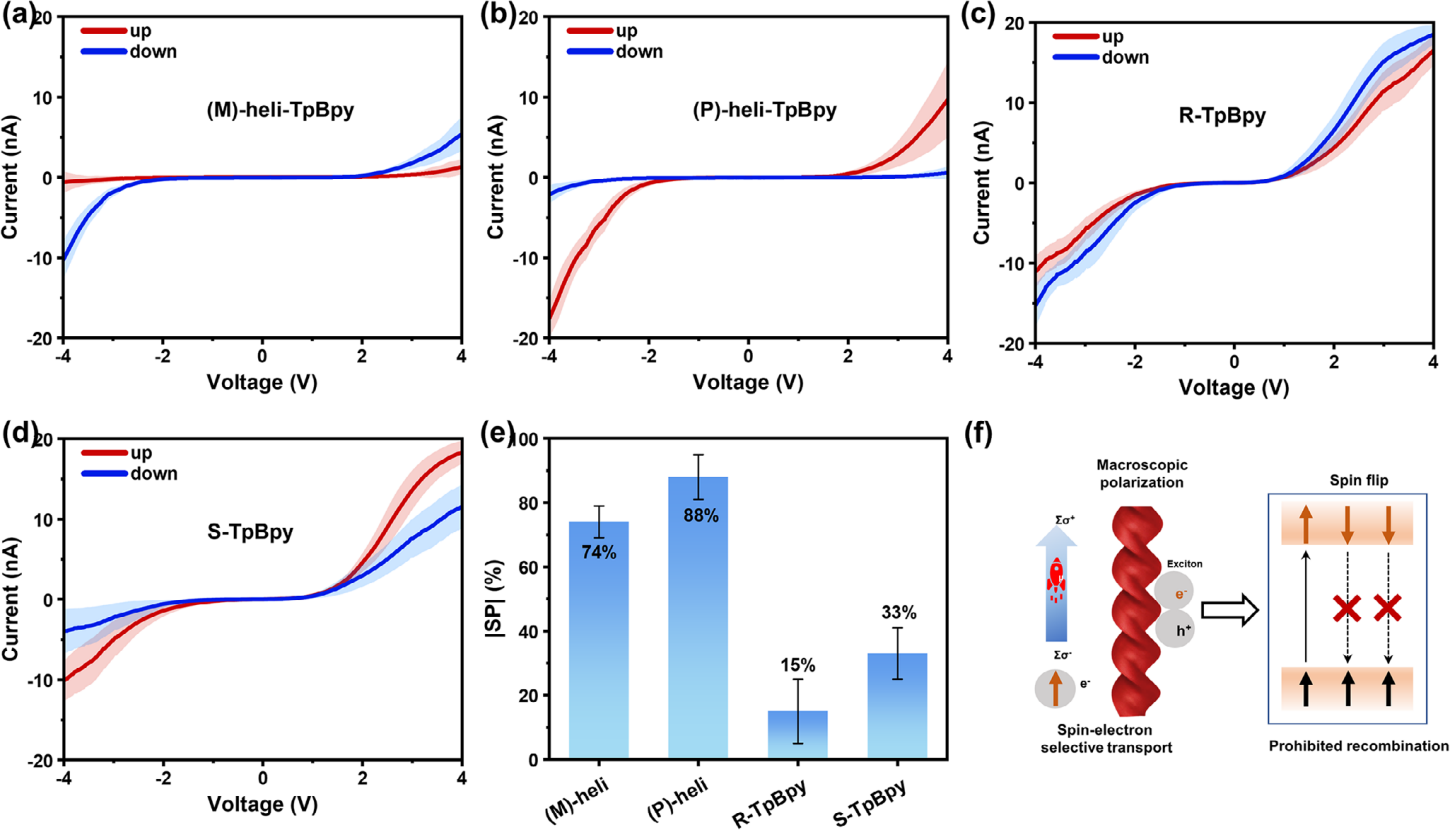
(a–d) Average I‐V curves for (a,b) (*M*)‐ and (*P*)‐heli‐TpBpy, (c,d) weakly chiral *R*‐ and *S*‐TpBpy, measured by mc‐AFM. (e) Spin polarization absolute values of different chiral TpBpy COFs. (f) Insight into the photocatalytic mechanism of the helical COF via CISS.

Based on the collective experimental evidence, we thoroughly investigated the electron spin polarization effect to elucidate the origin of chirality‐dependent photocatalytic performances. Magnetic current AFM (mc‐AFM) was employed to evaluate the spin selectivity of electrons transmitted through the helical TpBpy, which was deposited on an indium tin oxide (ITO)‐coated glass substrate. The ITO‐supported COF was subjected to a voltage sweep from −4 to +4 V, and averaged current‐voltage (I‐V) curves were recorded with the tip magnetized upward (red line) and downward (blue line). As displayed in Figure 5a–d, the influence of magnetic polarity on the I‐V response is more pronounced in the helical COF than in the weakly chiral COF. Specifically, (*M*)‐heli‐TpBpy gave a significantly higher current under a downwardly magnetized AFM tip compared to an upwardly magnetized one as the bias voltage increased. Also, this trend was completely reversed for (*P*)‐heli‐TpBpy, showing a much weaker current under downward magnetization. By contrast, the achiral TpBpy exhibited the indistinguishable I‐V responses for upward and downward tip magnetization, indicating the absence of spin‐selective transport in the achiral COF (Figure S39).

The anisotropy of the spin‐polarized current was quantitatively evaluated using the spin polarization (SP) parameter, which can be calculated by the equation SP = (*I*_down_ − *I*_up_) / (*I*_up_ + *I*_down_) × 100%, where *I*_up_ and *I*_down_ denote the current values measured in the upward and downward magnetization direction, respectively. (*P*)‐ and (*M*)‐heli‐TpBpy yielded average SP values of −74 ± 5% and 88 ± 7%, respectively, over a bias range of −4 to +4 V (Figure S40). This performance is among the best achieved by intrinsic COF‐based materials to date (Table S3). In comparison, the weakly chiral COFs rendered notably lower average SP values of −15 ± 10% and 33 ± 8% for *R*‐ and *S*‐TpBpy, respectively (Figure S41).

These results provide definitive evidence that the well‐defined helical conformation substantially enhances spin selectivity in the chiral COFs. Upon irradiation, the helical structure functions as an electron transport channel, wherein charge transfer is governed by the CISS effect. The pronounced spin‐orbit coupling inherent to the right‐handed helical structure facilitates the transmission of electrons with one spin orientation. Specifically, spin‐up electrons encounter a lower energetic barrier and experience reduced backscattering, leading to highly efficient transport along the helical axis. Conversely, electrons with the opposite spin (spin‐down) undergo strong scattering and significantly suppress transmission. As a result, the helical structure of TpBpy acts as an effective spin filter, promoting selective transport of one spin state over the other. Meanwhile, as illustrated Figure 5, spin‐orbit coupling causes spin flipping (e.g., from spin‐up to spin‐down), leading to the spin mismatch between electrons (spin‐down) and holes (spin‐up) [46, 47]. This effect enables the prohibition of exciton recombination pathway [48, 49, 50, 51, 52]. Consequently, spin polarization promotes the separation of photo‐generated electrons and holes and in turn, prolong charge‐separated lifetimes.

## 3 Conclusion

In summary, we present a noncovalent chirality transfer strategy for constructing highly crystalline, helically assembled COF nanofibers from achiral building blocks in a chiral solvent environment. We find that chiral chloropropylene glycol can trigger the oriented stacking of achiral bipyridine‐containing modules through the multiple noncovalent interactions at the early stage of the reaction, effectively transferring its chiroptical signatures within the layered structure of 2D COFs. Moreover, this chiral solvent environment dominates the helical assembly of the COF nanofibers, leading to significantly amplified chirality without compromising the high crystallinity of 2D COFs. Hence, the obtained helical COF shows exceptional spin polarization (∼74%‐88%), ranking among the highest reported for intrinsic COF systems. This outstanding property enhances photogenerated charge concentrations, suppresses exciton recombination, and extends carrier lifetime, resulting in enhanced photocatalytic performance. The helical COF achieves a hydrogen evolution rate nearly twice that of its achiral and weakly chiral counterparts. Our findings establish a noncovalent chirality‐induced method that enables synergistic control over COFs from molecular conformation to mesoscopic morphology during crystallization. More importantly, this work advances the exploitation of the intrinsic photocatalytic potential in chiral organic materials.

## Conflicts of Interest

The authors declare no conflicts of interest.

## Supporting information




**Supporting File**: advs75127‐sup‐0001‐SuppMat.docx.

## Data Availability

The data that support the findings of this study are available from the corresponding author upon reasonable request.
