# Supplementary material for: Helicalization of Covalent Organic Framework Nanofibers with Amplified Spin Polarizability for Boosting Photocatalytic Hydrogen Evolution
Source: Adv Sci (Weinh). 2026 Apr 3;13(36):e75127. doi: 10.1002/advs.75127 (PMC13317581; doi:10.1002/advs.75127)
Supplement: Supplementary file 1 — Supporting File: advs75127‐sup‐0001‐SuppMat.docx. [file ADVS-13-e75127-s001.docx]

Supporting information

Helicalization of Covalent Organic Frameworks Nanofibers with Amplified Spin Polarizability for Boosting Photocatalytic Hydrogen Evolution

Qi Zhong, Yongtu Tian, Yutao Sang*, Changchun Wang, and Jia Guo*

State Key Laboratory of Molecular Engineering of Polymers, Department of Macromolecular Science, Fudan University, Shanghai 200438, China

E-mail: sangyt@fudan.edu.cn (Y. Sang); guojia@fudan.edu.cn (J. Guo)

**Section 1. Materials and Methods**

**1. Materials**

Anhydrous mesitylene, n-butanol, 3-chloro-1,2-propanediol (3-MCPD), 1,2-propanediol (PD), acetic acid (HOAc), ascorbic acid and chloroplatinic acid hexahydrate were purchased from Aladdin Industrial Corporation. 1,3,5-triformylphloroglucinol (Tp) and 2,2'-bipyridine-5,5'-diamine (Bpy) were purchased from Jilin Chinese Academy of Sciences-Yanshen technology Co. Ltd. Tetrahydrofuran (THF) and ethanol were purchased from Sinopharm Chemical Reagent Co. Ltd. 5% Nafion solution was purchased from DuPont Company. All the chemical materials were used without further purification.

**2. Methods**

**2.1 Synthesis of helical TpBpy**

A Pyrex tube (10 mL) was charged with Tp (12.6 mg, 0.06 mmol), Bpy (16.8 mg, 0.09 mmol), n-butanol (0.5 mL), mesitylene (0.05 mL) and (*R*/*S*)-3-chloro-1,2-propanediol (0.2 mL). The mixture was sonicated for 5 min and then 6 M HOAc (0.1 mL) was added. The mixture was degassed through three freeze-pump-thaw cycles before sealing under vacuum. The sealed tube was kept at 120°C in an oven for 3 days. The precipitate was collected by filtration, washed with THF for several times. Then the product was Soxhlet extracted with THF for 24 h and dried under vacuum at 40°C for 24 h to collect red powder with a yield of 85%.

**2.2 Synthesis of achiral TpBpy**

Achiral TpBpy was synthesized with the same procedure as for helical TpBpy except that the achiral solvent of (±)- 3-chloro-1,2-propanediol was used.

**2.3 Synthesis of chiral TpBpy**

Chiral TpBpy was synthesized with the same procedure as for helical TpBpy except that the solvent ratio is different, which become n-butanol/mesitylene = 3/5.

**2.4 Photocatalytic hydrogen evolution**

The photocatalytic hydrogen evolution experiments were conducted in a Pyrex top-irradiation reaction vessel linked to a glass closed Labsolar 6A gas circulation system (Perfect Light, China). For each test, the photocatalyst (10 mg) was dispersed in the aqueous solution of ascorbic acid (0.1 mol L^–1^, 100 mL) and added with H_2_PtCl_6_ aqueous solution (1 wt% Pt loading) for photo-deposition of Pt as cocatalyst. Before the photocatalysis, the mixture was sonicated for 30 min to obtain a homogeneously dispersed solution and evacuated several times to remove air completely. The 300 W Xe lamp equipped with a cut-off filter (> 420 nm) irradiated on the reaction system through a quartz transparent glass on the top of the vessel. The temperature of the reaction solution was kept under 10 °C by cooling water. The gas product was analyzed by gas chromatography (Techcomp GC7900, China) equipped with a thermal conductivity detector referencing against standard gas with a known concentration of hydrogen. After the photocatalysis test, the samples were recovered by thoroughly rinsing and drying at 40°C under vacuum.

**2.5 The AQE measurement**

The apparent quantum efficiency (AQE) was measured under the irradiation of a 300W Xe lamp equipped with different bandpass filters (including 450 nm, 500 nm, 550nm, 600 nm, 650 nm) using the following equation.

$\eta_{AQE}=\frac{2\times M\times N_{A}}{\frac{E_{total}}{E_{photon}}}\times100\%=\frac{2M\times N_{A}}{\frac{S\times P\times t}{\hbar\times\frac{c}{\lambda}}}\times100\%=\frac{2\times M\times N_{A}\times h\times c}{S\times P\times t\times\lambda}\times100\%$

Where, *M* is the amount of H_2_ molecules (mol), *N_A_* is Avogadro constant (6.022×10^23^ mol^-1^), *h* is the Planck constant (6.626 × 10^-34^ J s), *c* is the speed of light (3×10^8^ m s^-1^), *S* is the irradiation area (cm^2^), *𝑃* is the intensity of irradiation light (W cm^-2^), *t* is the photoreaction time (s), and 𝜆 is the wavelength of the monochromatic light (m).

**2.6 Photoelectrochemical measurements**

Photocurrent response tests, electrochemical impedance spectra (EIS) and Mott-Schottky plots were recorded on the CHI760E electrochemical workstation (Chenhua, China). 2 mg samples dispersed in a mixture of 200 μL methanol, and 10 μL 5% Nafion solution was sonicated for 30 min and then the mixture was coated on the indium-tin oxide (ITO) glass to form a film (1 cm^2^) as the working electrode. Pt wire was used as the counter electrode, the Ag/AgCl electrode as a reference electrode and 0.5 M Na_2_SO_4_ (*pH* = 6.8) as the electrolyte. 300 W Xe lamp equipped with a cut-off filter (>420 nm) was used as light source.

The voltammetry behaviors of the photocatalysts before and after Pt deposition were characterized using linear sweep voltammetry at the scan rate of 5 mV s^-1^. 0.5 M Na_2_SO_4_ was applied as the electrolyte. Pt wire was the counter electrode and the Ag/AgCl electrode was the reference electrode. 6 μL dispersion of samples was drop cast onto the polished glass carbon electrode to form the working electrode. The overpotential was defined as the potential at the current density of 10 mA cm^-2^.[1]

The applied potentials vs. Ag/AgCl are converted to RHE potentials using the following equation,

$$E_{RHE}= E_{Ag/AgCl}+0.0591pH+E_{Ag/AgCl}^{\theta} (E_{Ag/AgCl}^{\theta}=0.199 V)$$

**2.7 Calculations**

Quantum chemistry calculations with Gaussian 16 were performed to evaluate the relative position of TpBpy and 3-chloro-1,2-propanediol. The geometry structures were optimized in the gas phase using the B3LYP/6-31G (d, p). In order to study the interaction between TpBpy and 3-chloro-1,2-propanediol, Electrostatic potential (ESP) maps and non-covalent interaction (NCI) analysis based on the optimized structure were obtained from the wave function file of DFT calculation using Multiwfn.[2]

**2.8 The mc-AFM measurements**

The magnetic conductive atomic force microscopy (mc-AFM) enables spin-dependent current measurements at the interface under different voltages by adjusting the magnetization direction of the tip.[3] Before conducting the experiments, 2 mg COF powder were dispersed in 20 mL ethanol. Then the mixture was sonicated for 2 hours with a cell crusher to achieve complete dispersion and uniformity. After allowing sedimentation overnight, 20 μL of supernatant was put on the indium tin oxide (ITO)-coated glass substrates by spin coating, producing a relatively flat film (1 cm^2^). Throughout the experimental process, the AFM tip was held at a constant ground, with the potential of the substrate adjusted between −4 and +4 V. A magnetic Pt-Cr coated tip (HQ: NSC18/Co-Cr/AI), with a nominal spring constant of 2.8 N/m, was used to acquire I-V curves. The tips are pre-magnetized using a ~ 4 T permanent magnet, resulting in an upward magnetization when pointing towards the magnet's north pole and a downward magnetization when pointing towards the south pole.

**3. Characterizations**

Powder X-ray diffraction (PXRD) patterns were collected at room temperature on an X-ray diffraction spectrometer (Smartlab 9 kW, Japan) with Cu Kα radiation at λ = 0.154 nm operating at 45 kV and 200 mA. Fourier transform infrared (FT-IR) spectra were recorded on Nicolet 6700 (Thermofsher, USA) Fourier transformation infrared spectrometer. UV-vis absorption spectra were collected on a Lambda 750 spectrometer (referenced to barium sulphate). Circular dichroism (CD) spectra were acquired using the CD spectrometer of Applied Photophysics Ltd (Chirascan, English). The photoluminescent spectra were recorded on the Edinburgh FLS1000 photoluminescence spectrometer. The lifetimes were examined with a 450-nm diode laser using Time-Correlated Single Photon Counting (TCSPC) technique and calculated by fitting with first-order exponential curve. Scanning electron microscopy images were obtained using a field emission scanning electron microscope (GeminiSEM 560, Germany) operated at 3 kV accelerating voltage. Transmission electron microscopy images were obtained using a high contrast transmission electron microscope (HT7800, Japan) operated at 80 kV accelerating voltage. N_2_ adsorption-desorption isotherms were collected by a TriStar II 3020 volumetric adsorption analyzer (Micromeritics, USA) at 77 K. The samples were degassed at 120 °C for 12 h under vacuum before measurement. The surface areas were evaluated using the Brunauer-Emmett-Teller (BET) model applied between P/P_0_ values of 0.05 and 0.1 for COFs. The platinum content of materials after photo-deposition was tested by inductively coupled plasma optical emission spectroscopy (ICP-OES) using the Agilent 5110 spectrometer. X-ray photoelectron spectroscopy (XPS) measurements were analyzed using a ULVAC-PHI XPS instrument (PHI Genesis 500, Japan) equipped with an Al K source (1486.6 eV). Thermogravimetric analysis (TGA) was conducted on a Pyris 1 Thermo Gravimetric Analyzer (PE, USA) at a heating rate of 10 ℃/min from 100 ℃ to 800 ℃ under air atmosphere. The electrochemical measurements were recorded on the CHI760E electrochemical workstation (Chenhua, China) with a standard three electrode system with the photocatalyst-coated ITO as the working electrode, Pt wire as the counter electrode and the Ag/AgCl electrode as a reference electrode. The electrolytes were bubbled with Nitrogen for 30 min before the measurement. The surface photovoltage spectra were acquired on a Surface photovoltage spectrometer (CEL-SPS1000). Current-voltage measurements were performed using Multimode AFM with Nanoscope V controller (Oxford Cypher VRS1250, USA). I-V spectroscopy measurements were recorded at the tip in a contact mode. For each spectroscopy measurement, the tip was placed in a new position.

**Section 2. Figures and Tables**

**
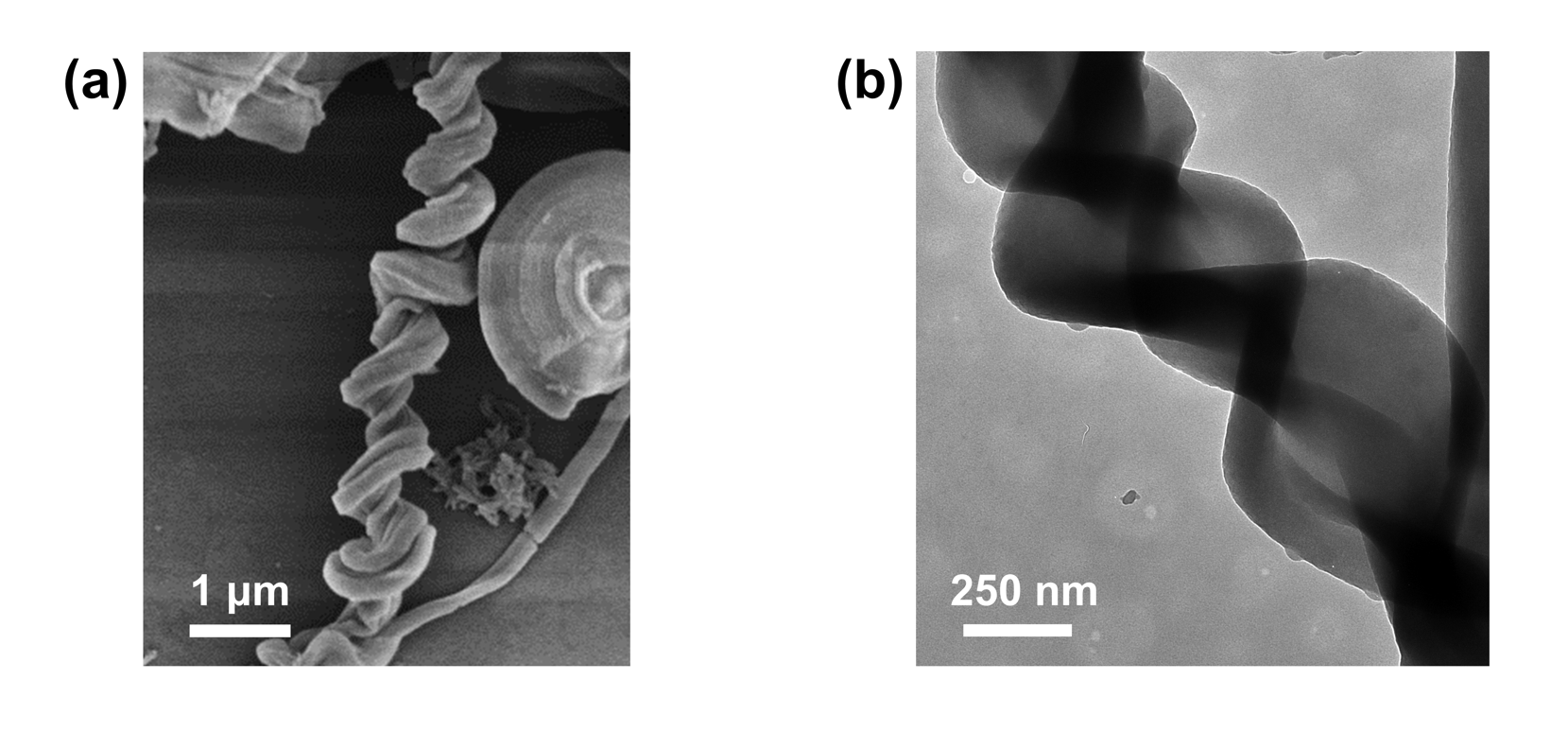
**

**Figure S1.** (a) SEM and (b) TEM images of (*P*)-heli-TpBpy.

**
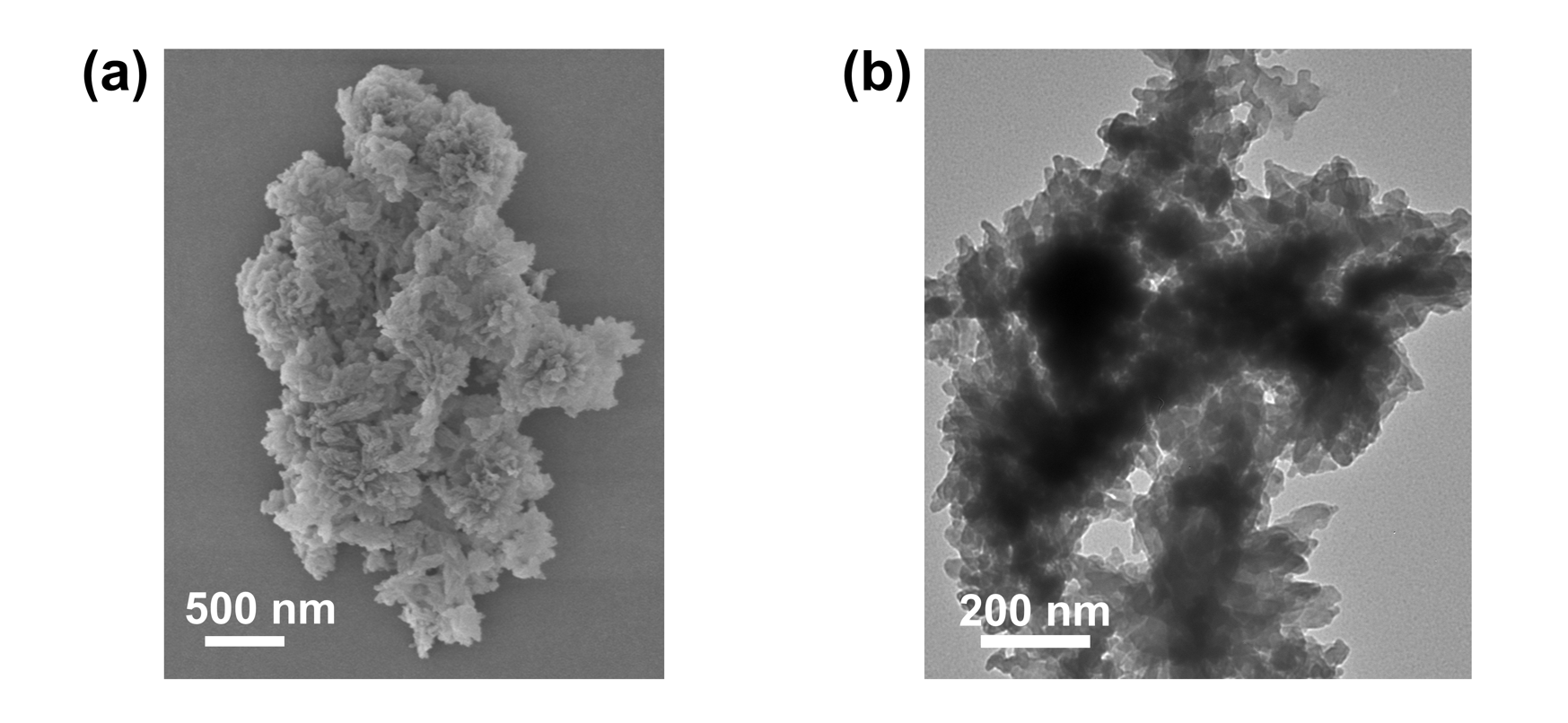
**

**Figure S2.** (a) SEM and (b) TEM images of *S*-TpBpy.


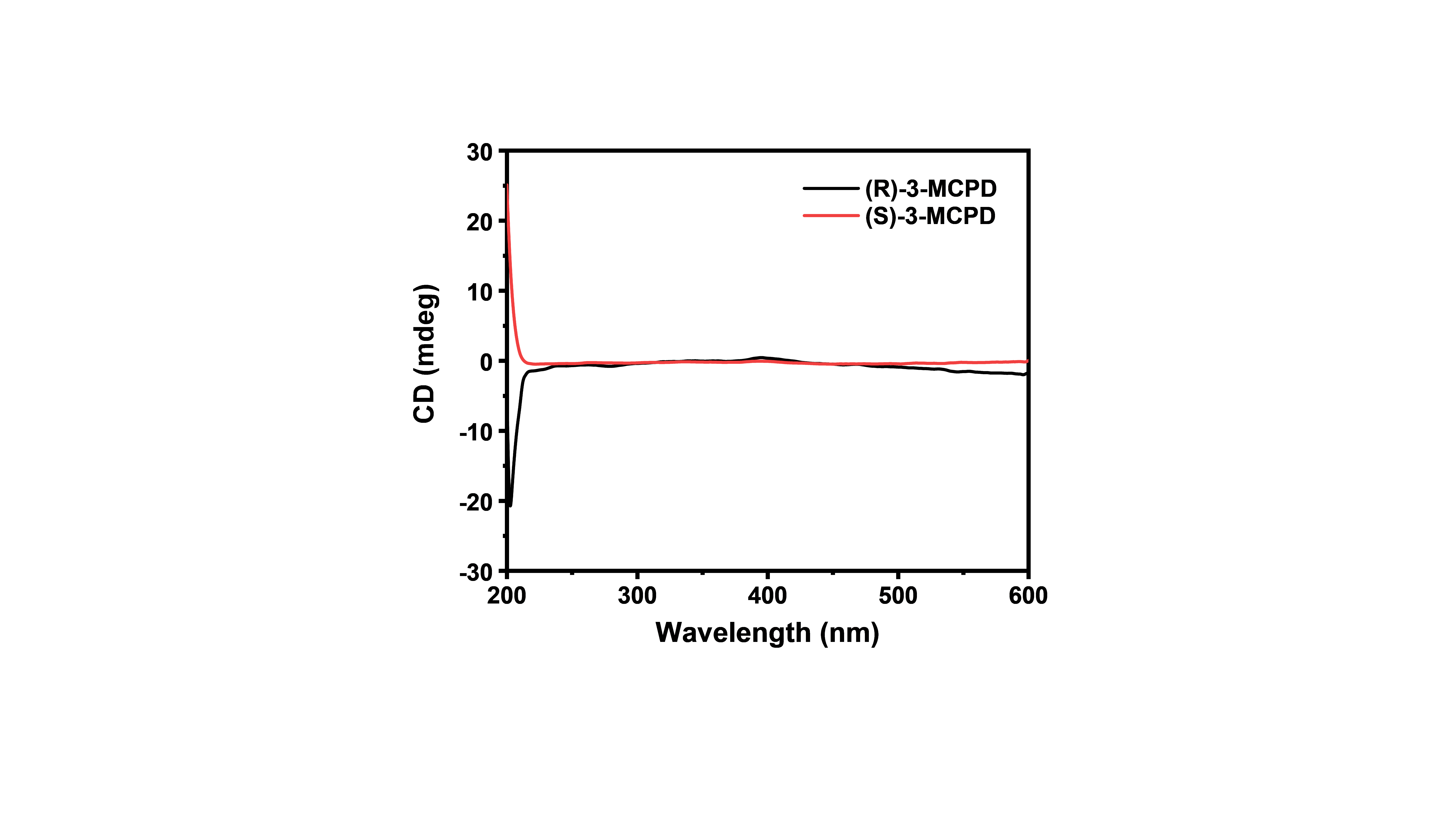


**Figure S3.** Circular dichroism (CD) spectra of chiral 3-chloro-1,2-propanediol.


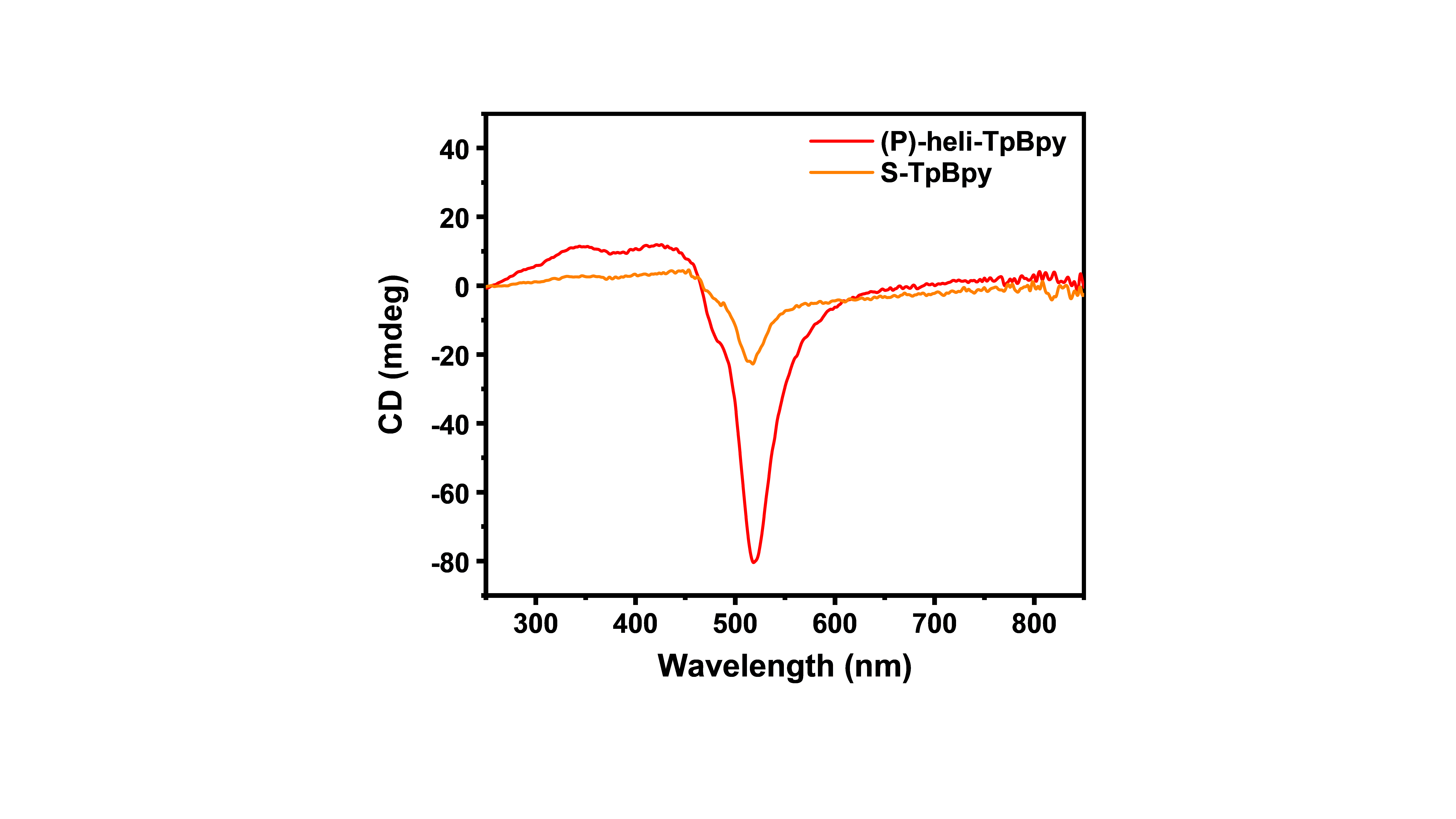


**Figure S4.** CD spectra of (*P*)-heli-TpBpy and *S*-TpBpy.


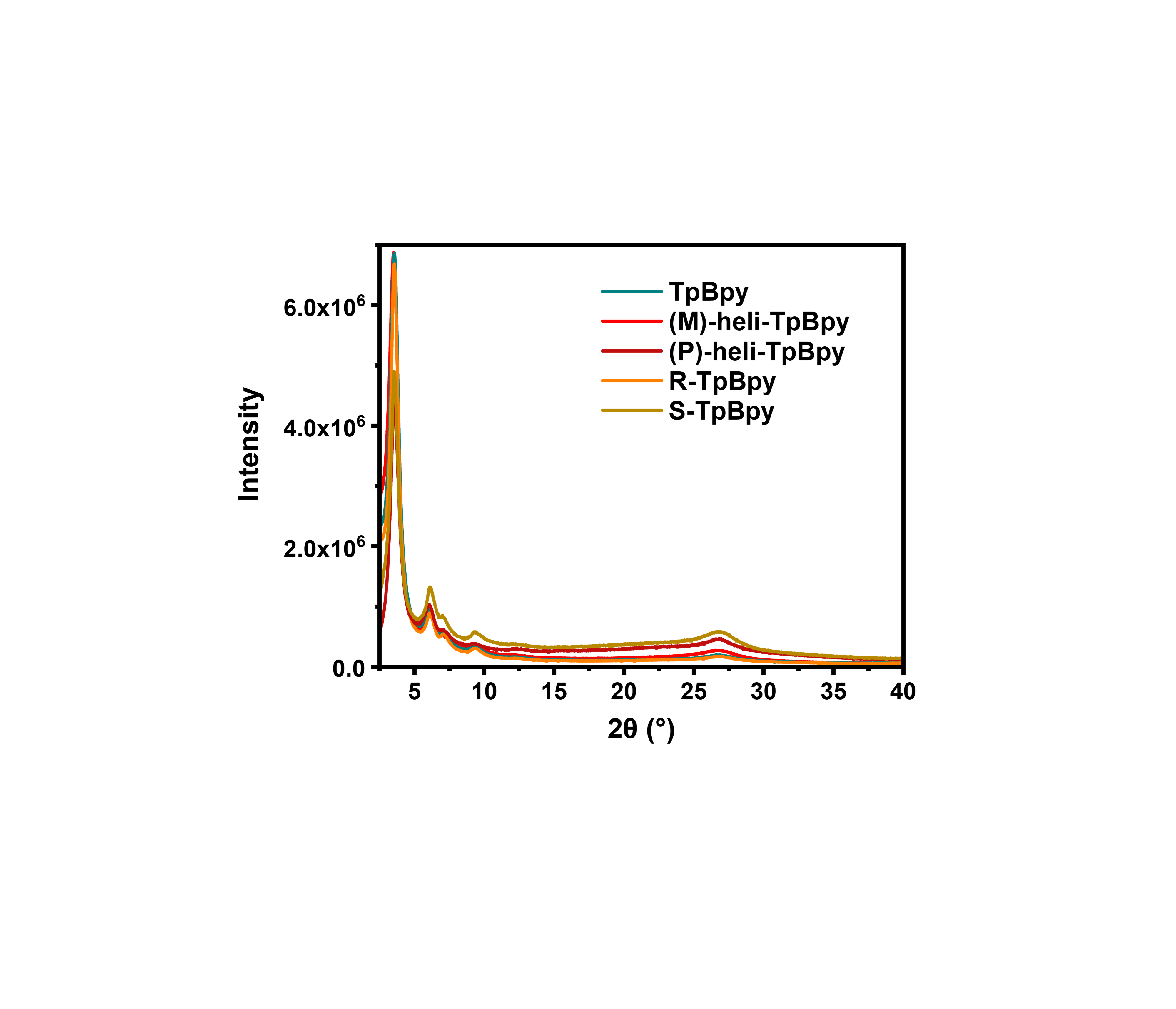


**Figure S5.** PXRD patterns of TpBpy, *R/S*-TpBpy and (*M/P*)-heli-TpBpy with equivalent quality.

**Table S1.** Full width at half maxima (FWHM) of (100) plane.

| **Sample** | **FWHM (°)** |
| --- | --- |
| TpBpy | 0.66 |
| *R*-TpBpy | 0.61 |
| *S*-TpBpy | 0.64 |
| (*M*)-heli-TpBpy | 0.70 |
| (*P*)-heli-TpBpy | 0.72 |


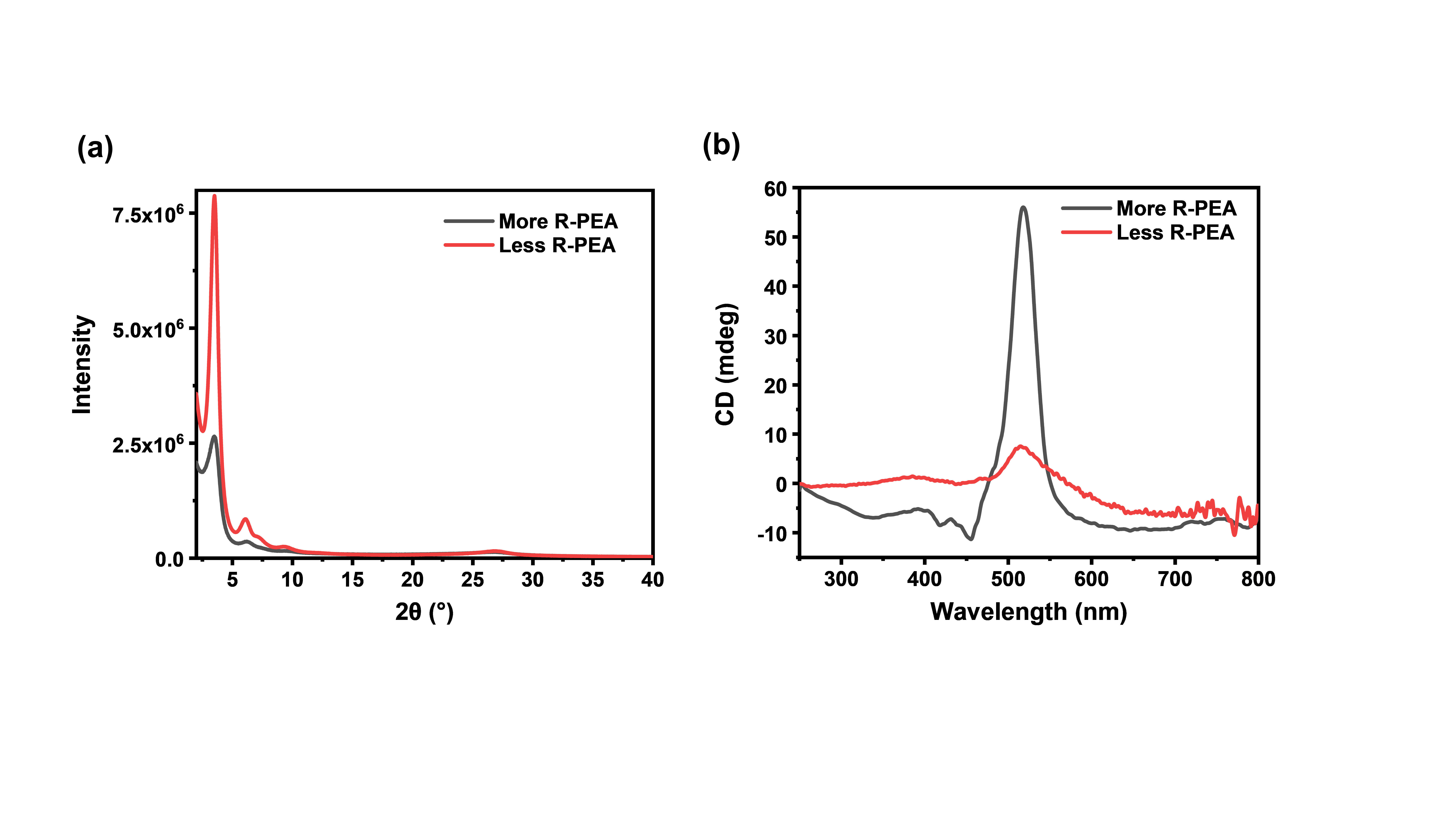


**Figure S6.** (a) PXRD patterns and (b) CD spectra of chiral TpBpy by using different doses of (*R*)-1-phenylethylamine (*R*-PEA).


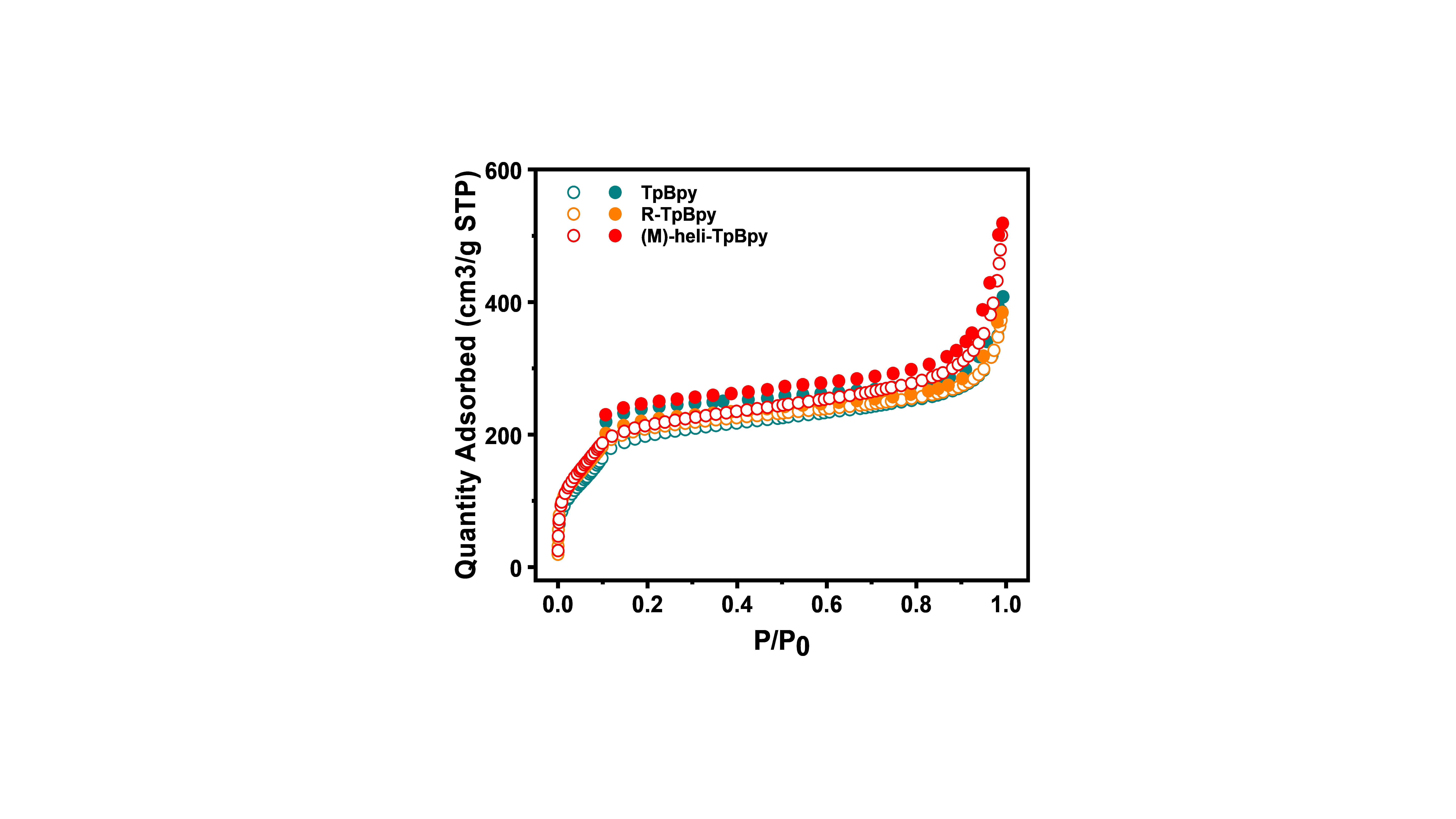


**Figure S7.** N_2_ adsorption and desorption isotherms of TpBpy COFs.


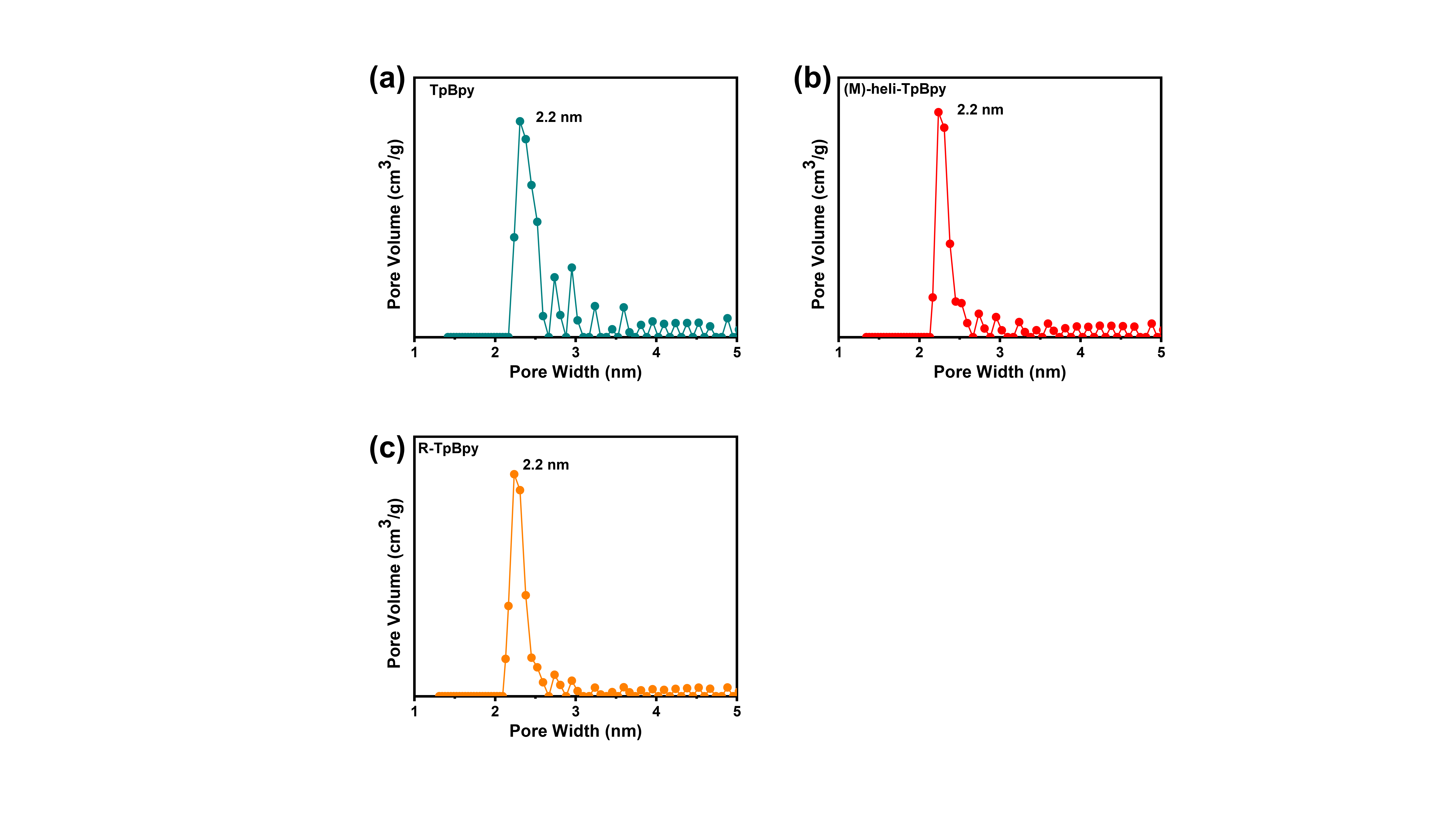


**Figure S8.** The pore-size distribution of (a) TpBpy; (b) (*M*)-heli-TpBpy and (c) *R*-TpBpy.


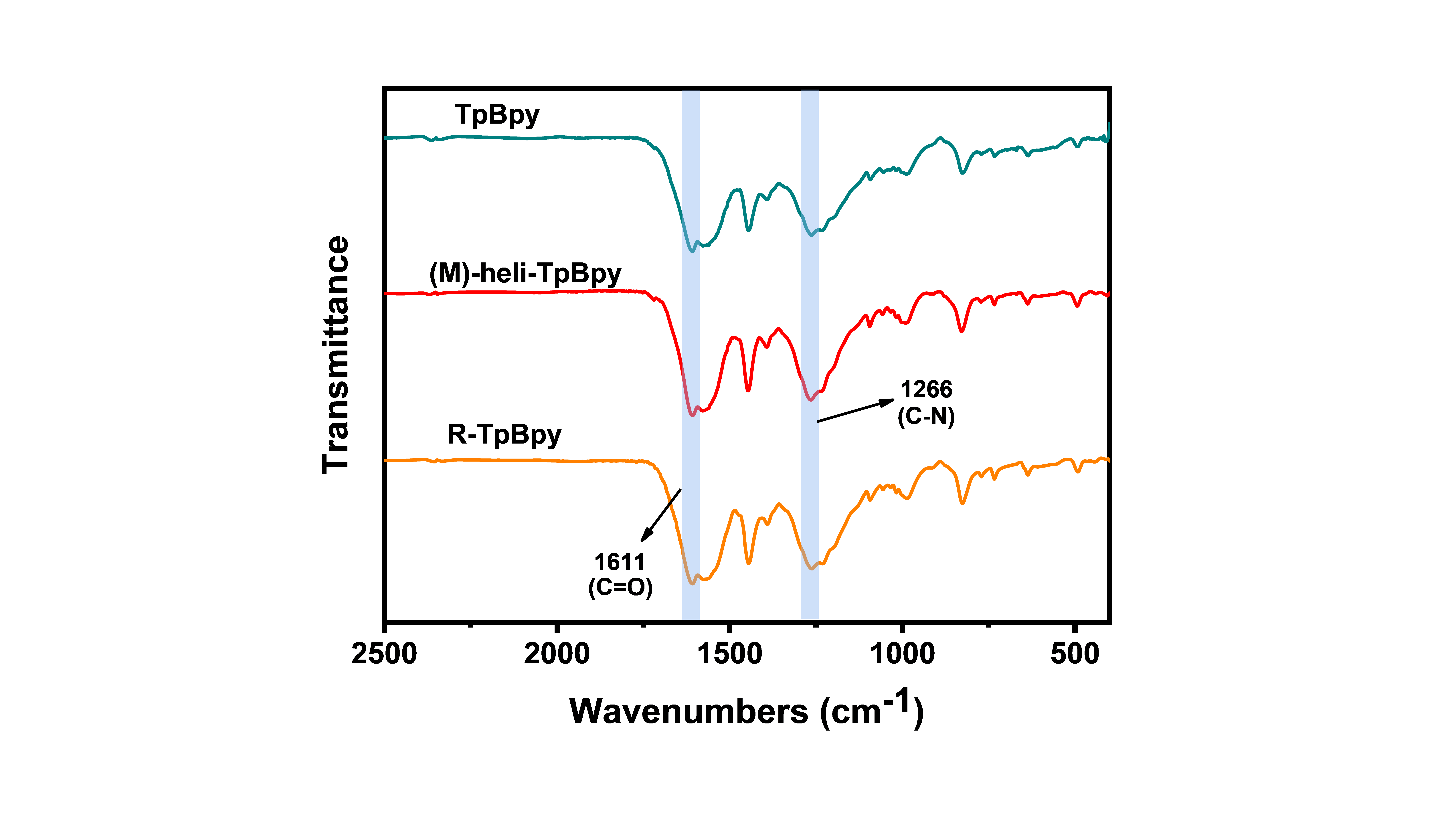


**Figure S9.** FTIR spectra of TpBpy, (*M*)-heli-TpBpy and *R*-TpBpy, respectively.


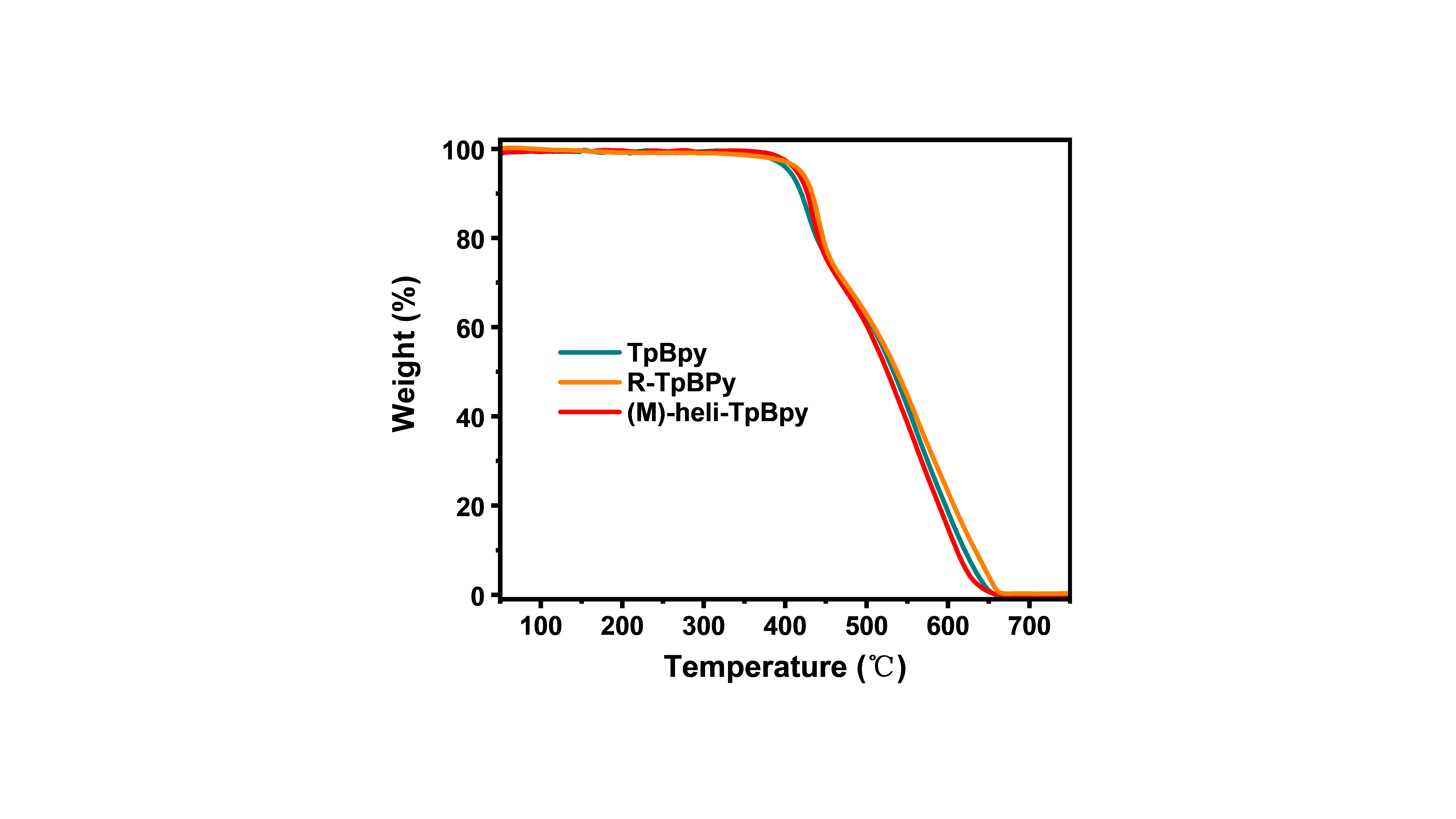


**Figure S10.** TGA curve of TpBpy COFs.


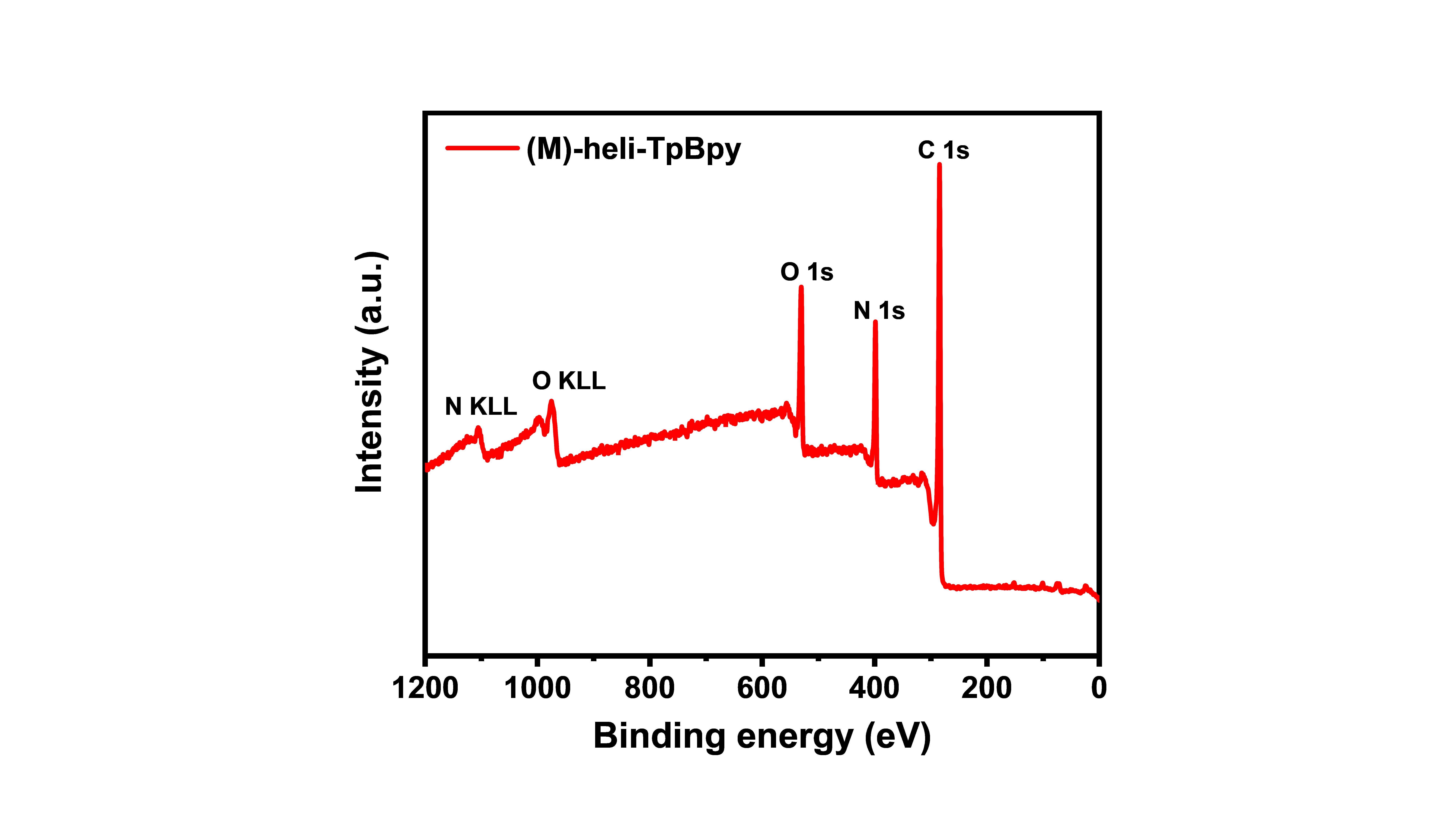


**Figure S11.** X-ray photoelectron spectroscopy (XPS) spectrum of (*M*)-heli-TpBpy.


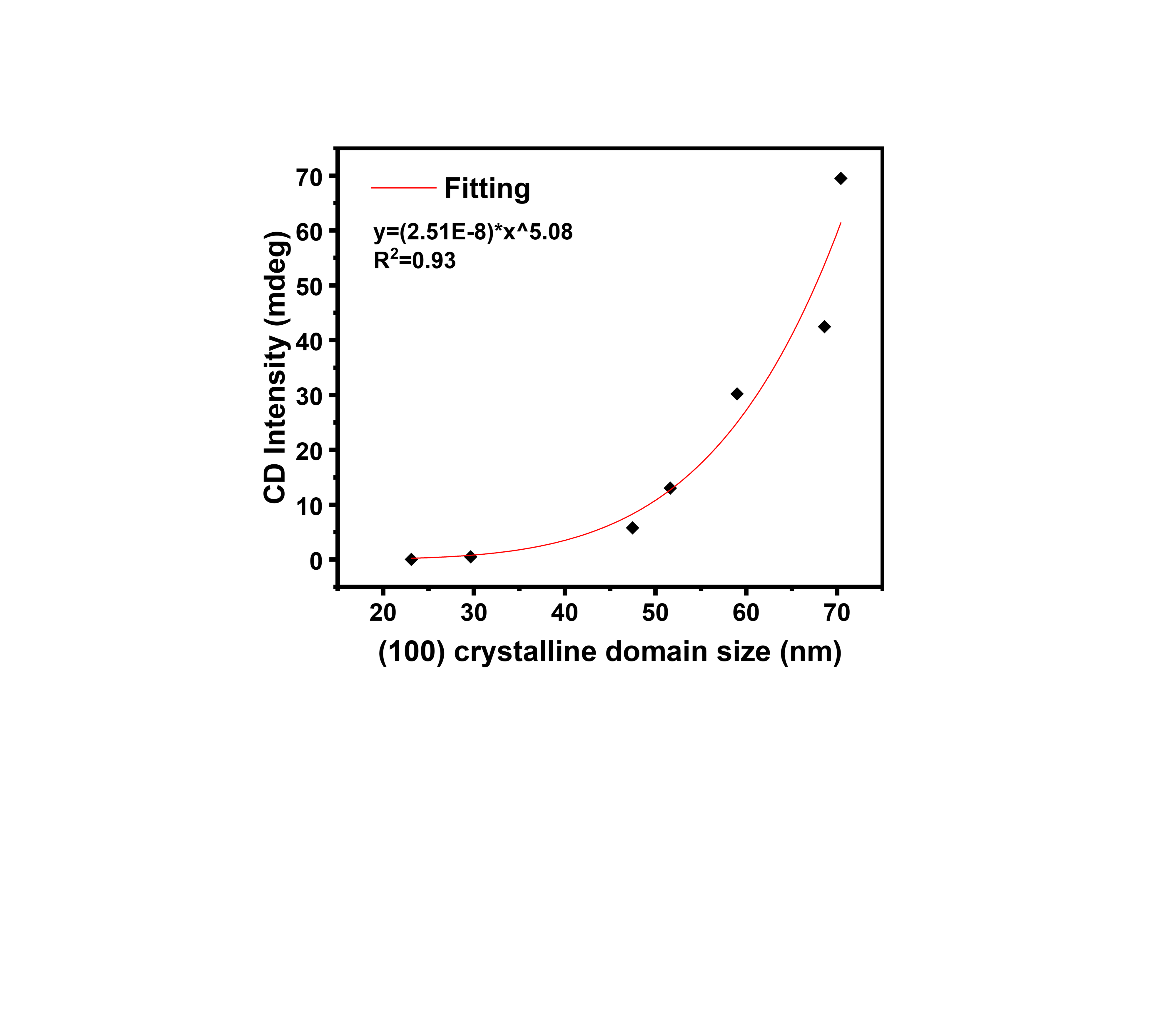


**Figure S12.** The correlation between crystalline domain size of (100) and CD intensity in different reaction time.


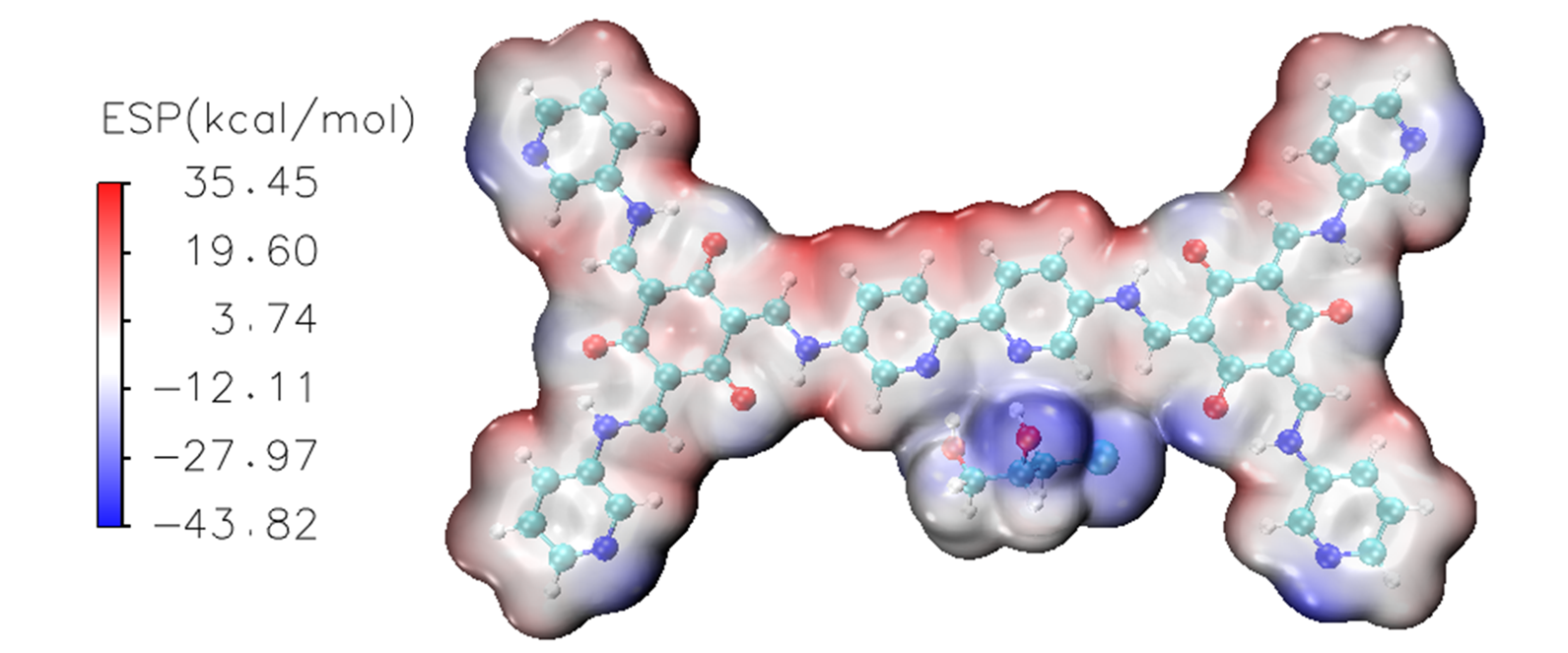


**Figure S13.** Electrostatic potential (ESP) mapping of the TpBpy moiety and (*R*)-3-MCPD molecule.


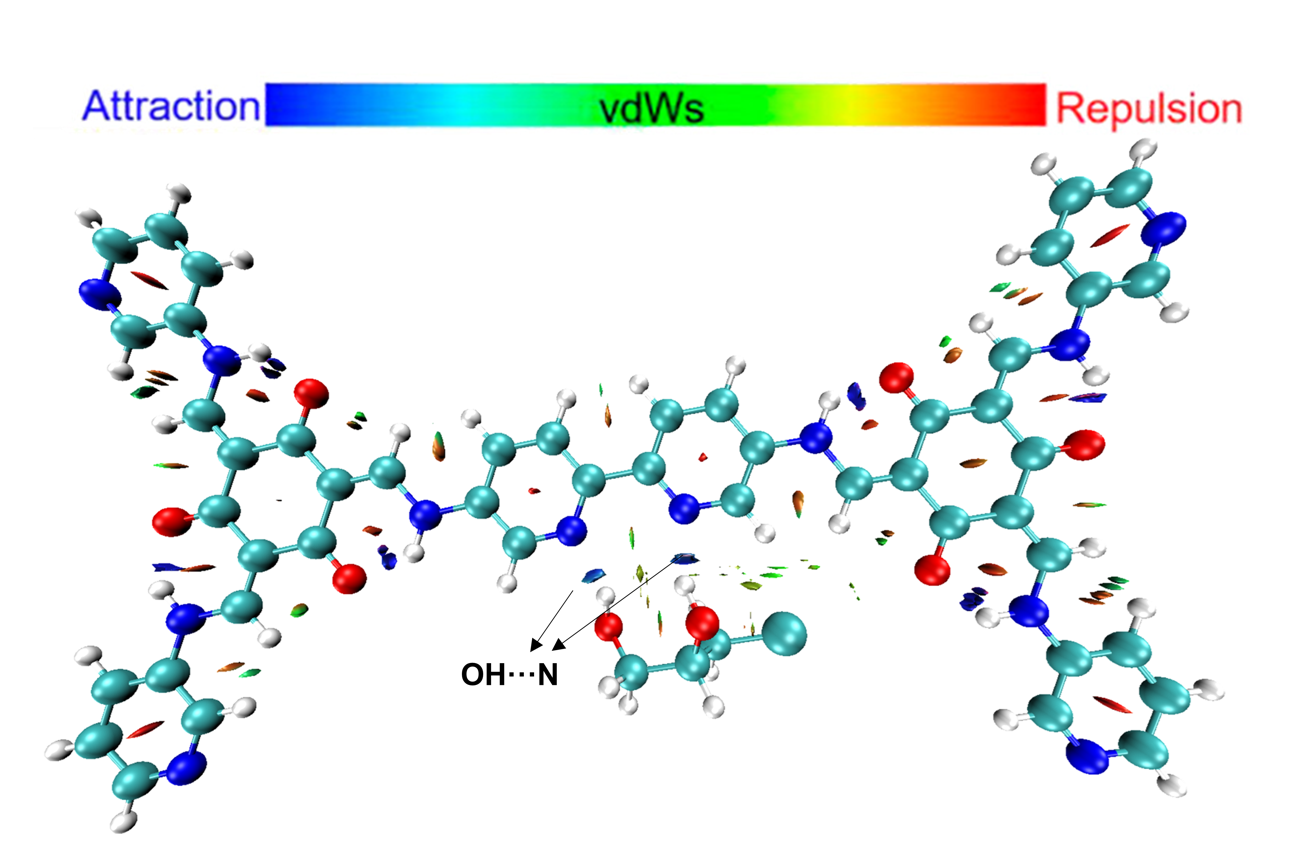


**Figure S14.** Noncovalent interaction analysis of the TpBpy moiety and (*R*)-3-MCPD molecule.


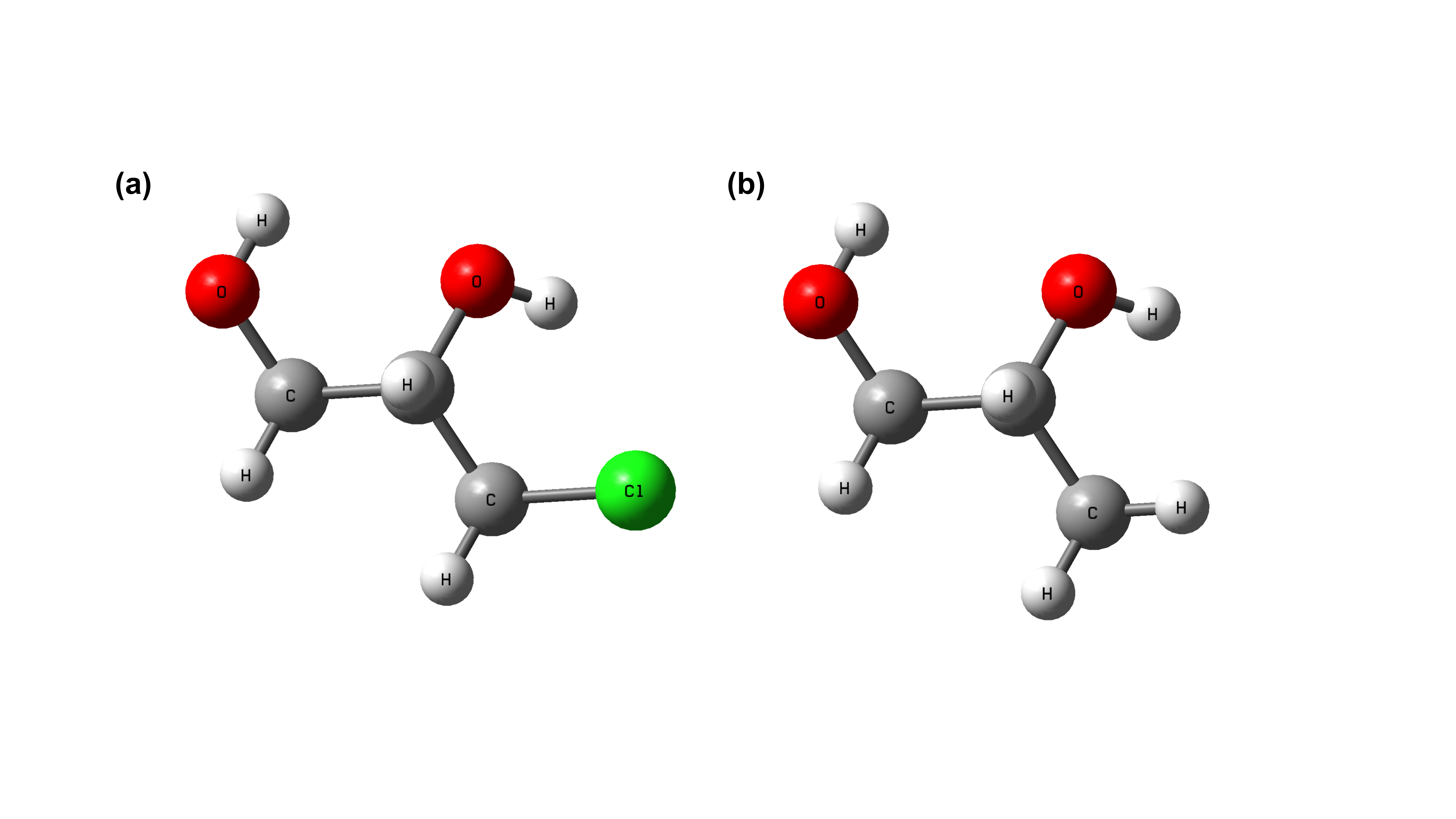


**Figure S15.** Ball-and-stick model of (a) 3-chloro-1,2-propanediol and (b) 1,2-propanediol.


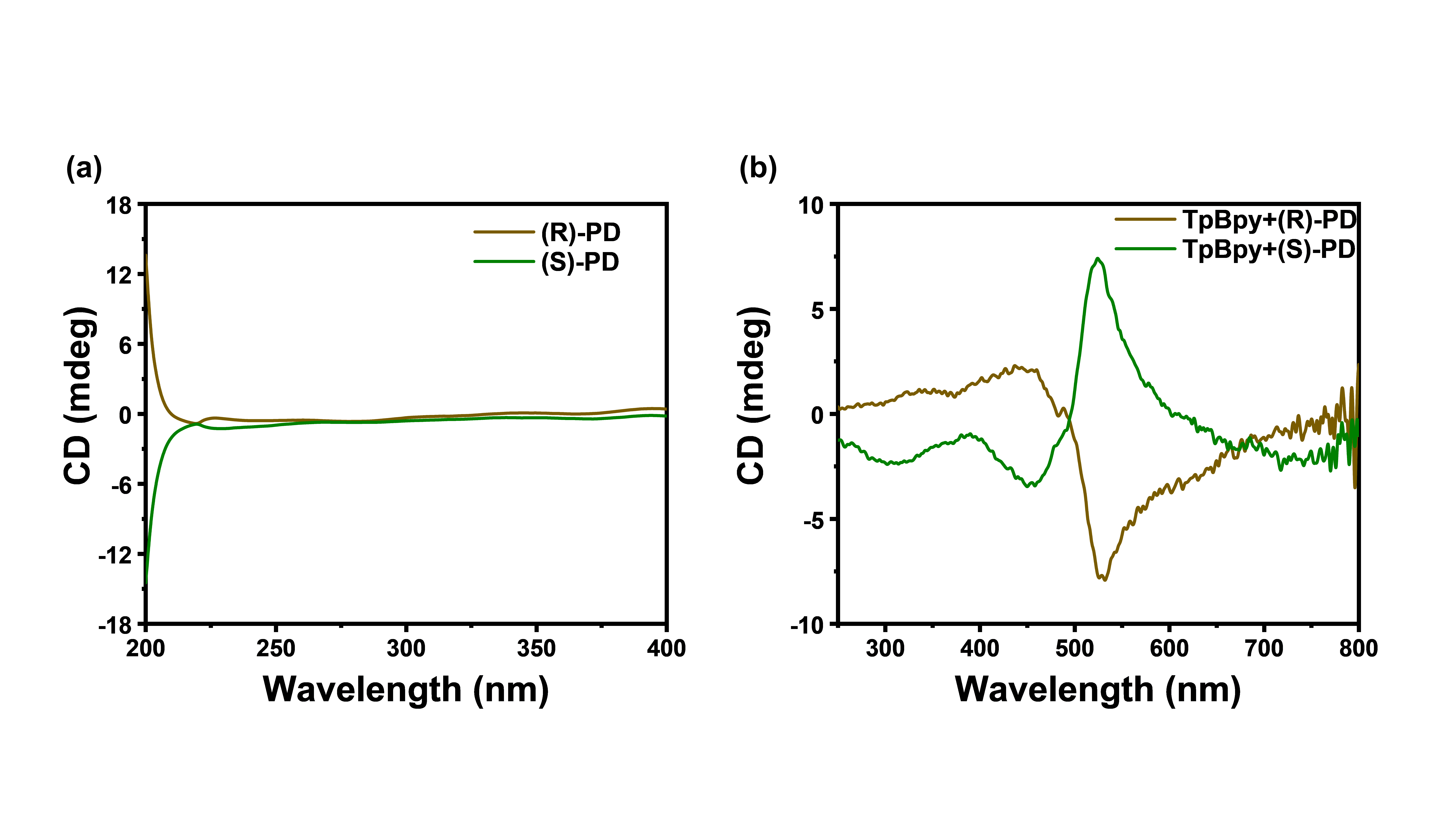


**Figure S16.** CD spectra of (a) chiral 1,2-propanediol and (b) chiral TpBpy induced by (*R/S*)-1,2-propanediol.


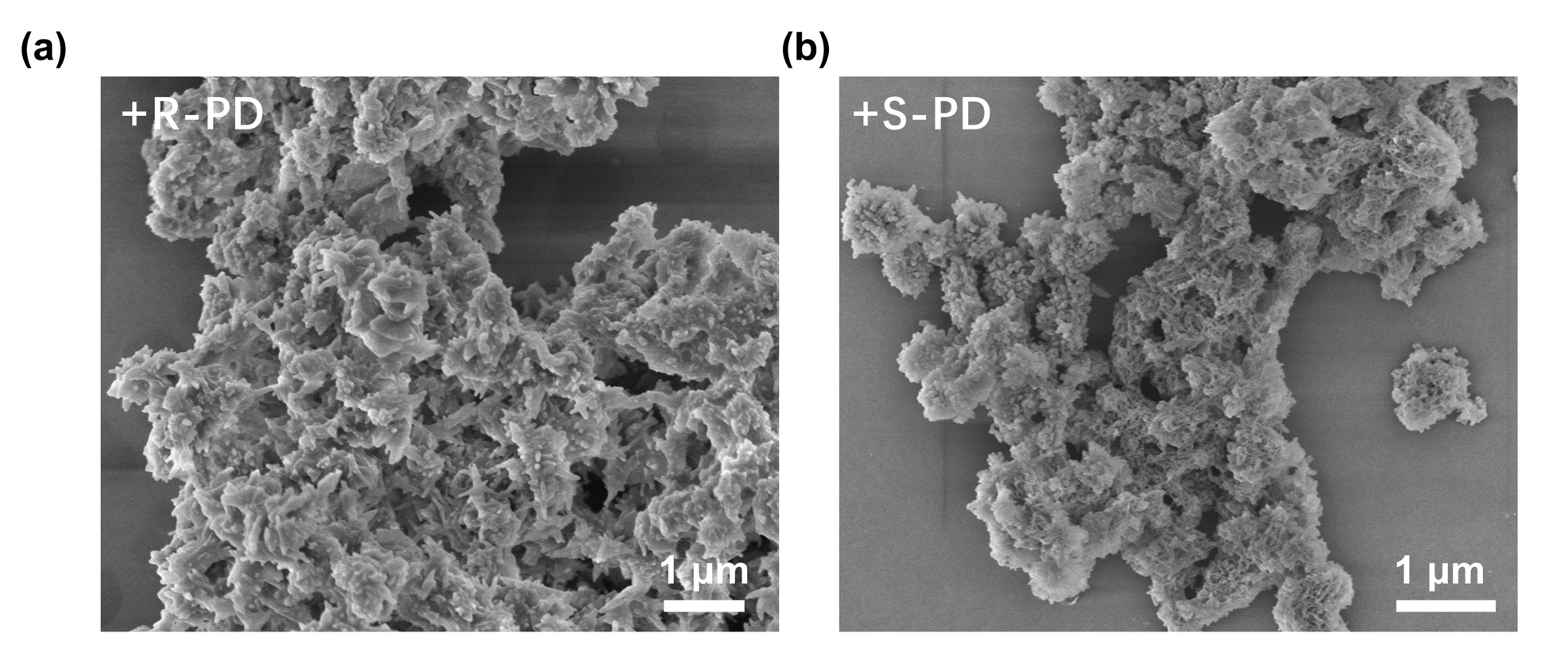


**Figure S17.** SEM images of chiral TpBpy induced by (a) (*R*)-1,2-propanediol (*R*-PD) and (b) (*S*)-1,2-propanediol (*S*-PD).

**
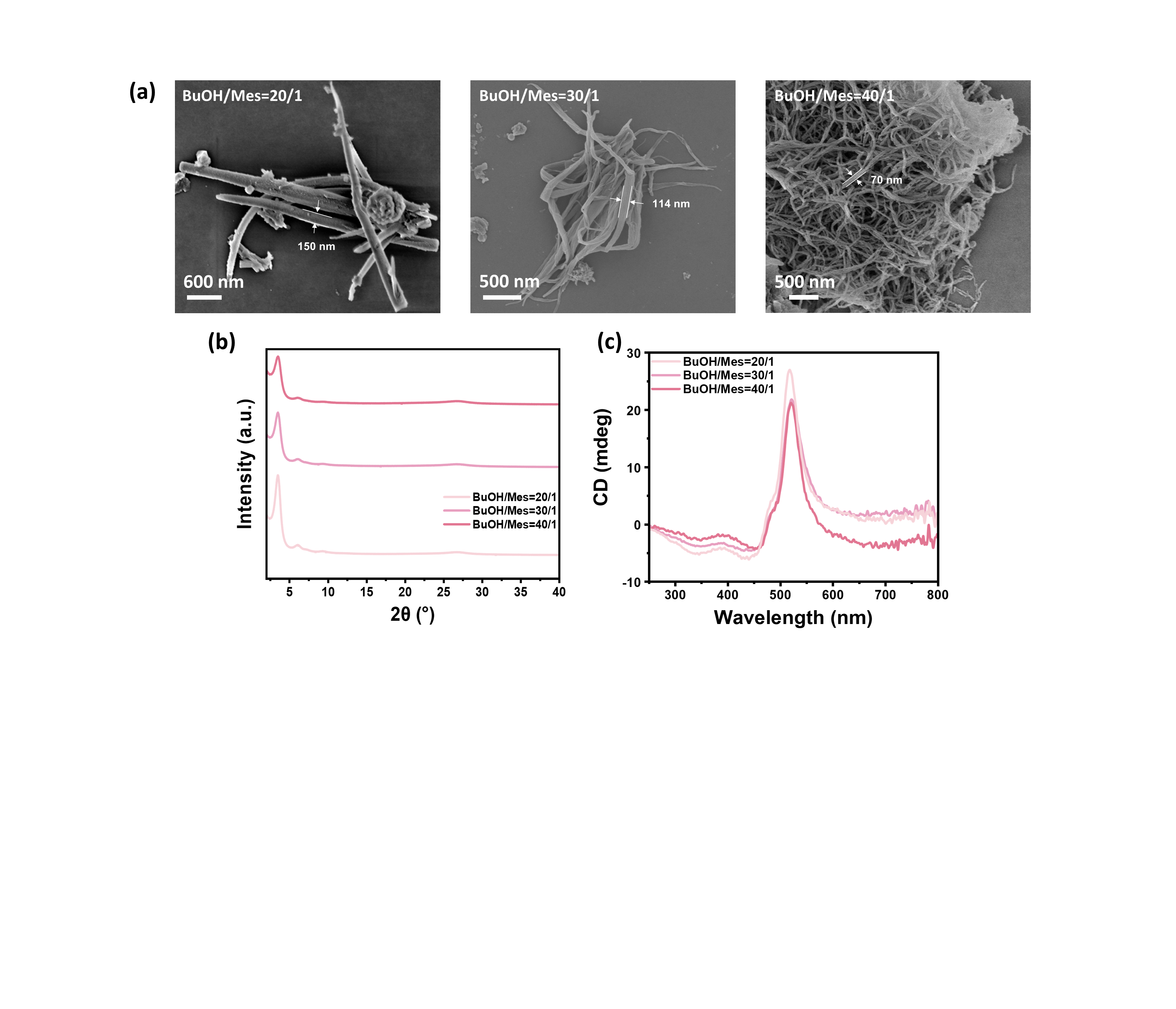
**

**Figure S18.** (a) SEM images, (b) PXRD patterns, and (c) CD spectra of a series of TpBpy synthesized by varying the ratio of BuOH/Mes while maintaining the amount of *R*-3-MCPD added.


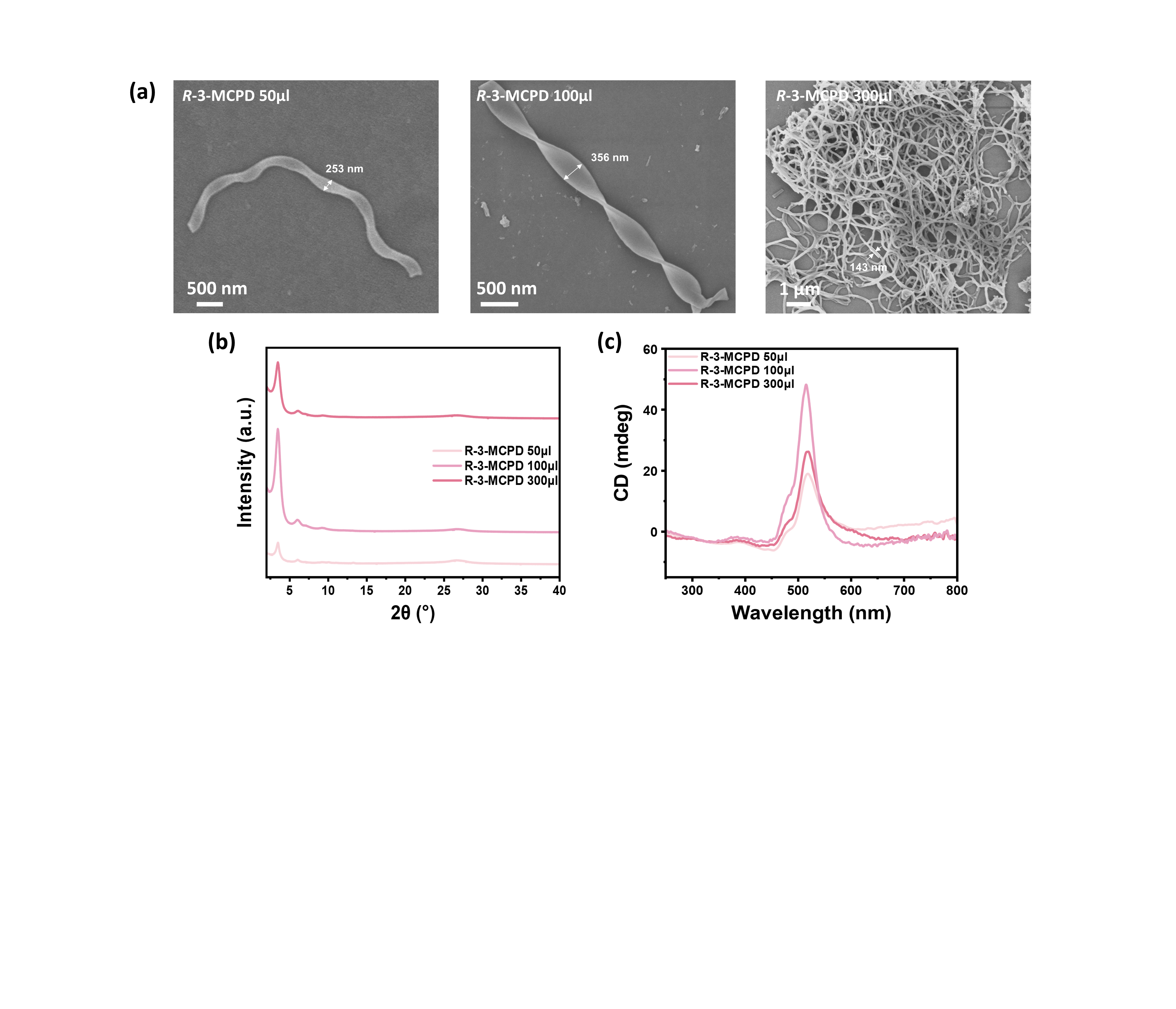


**Figure S19.** (a) SEM images, (b) PXRD patterns, and (c) CD spectra of a series of TpBpy synthesized by varying the amount of *R*-3-MCPD while maintaining the ratio of BuOH/Mes (10/1, v/v).


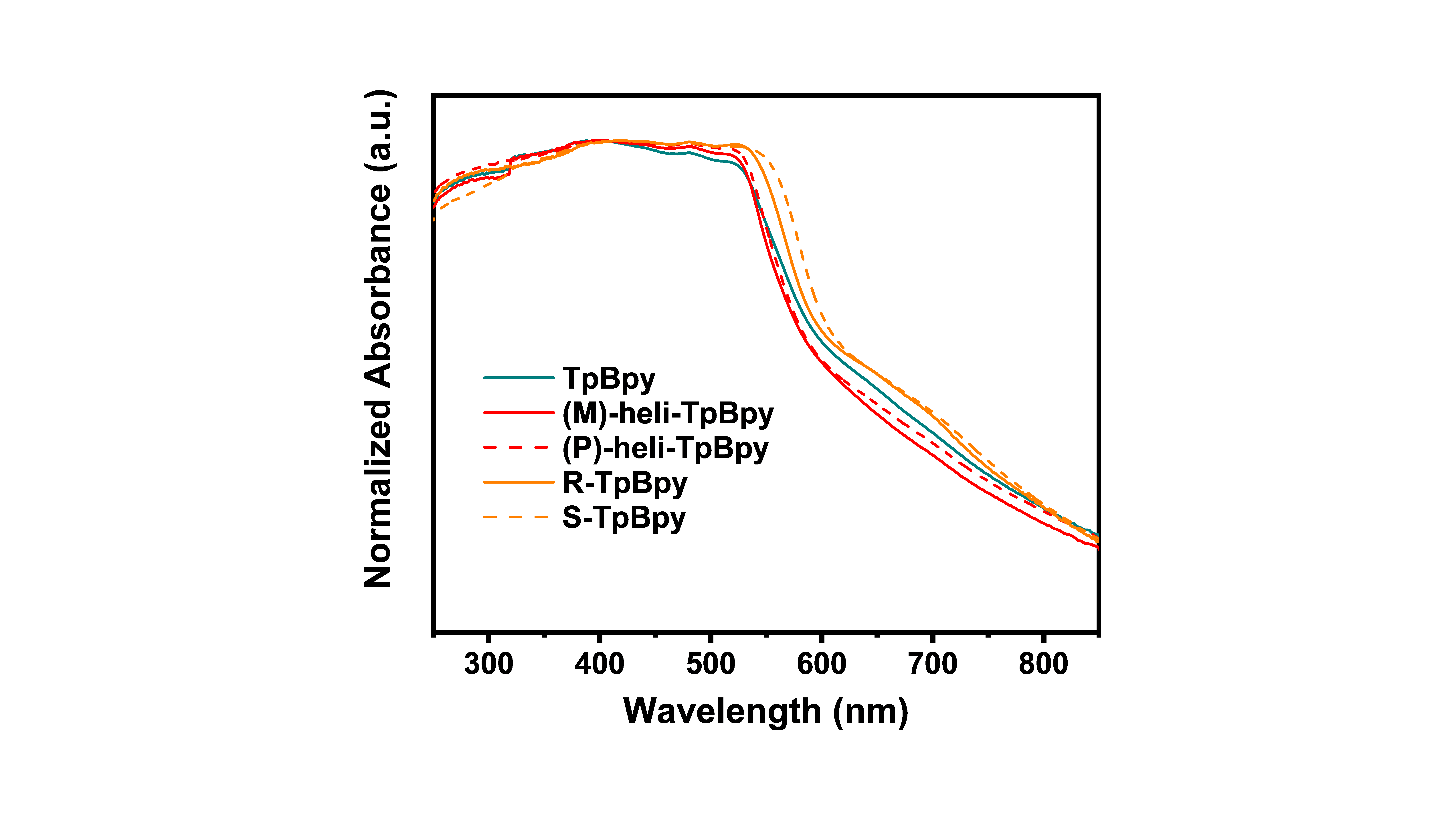


**Figure S20**. Solid-state UV-vis diffuse reflection spectra of photocatalysts.


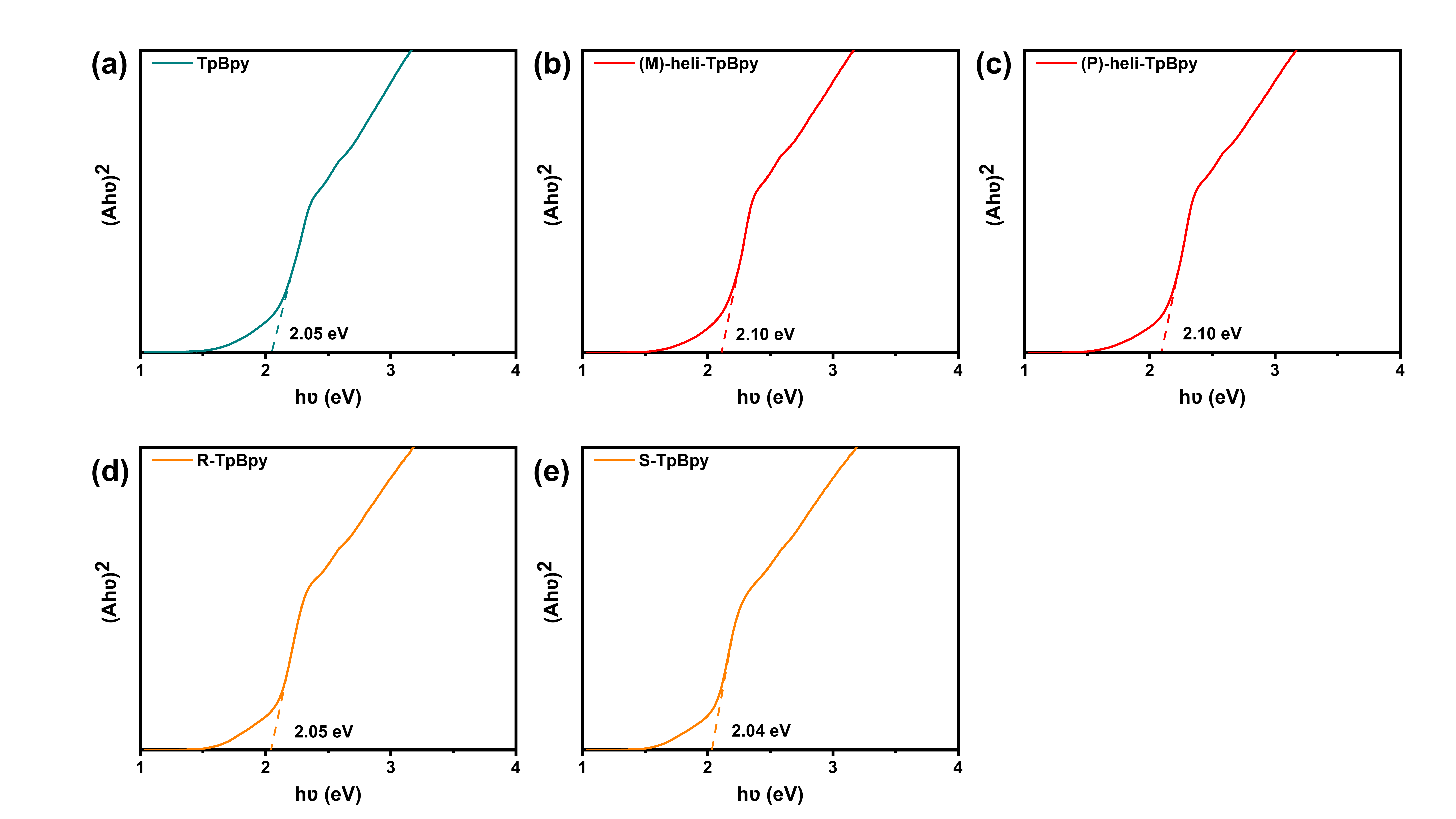


**Figure S21.** Tauc plots of (a)TpBpy, (b, c) helical TpBpy, and (d, e) chiral TpBpy.


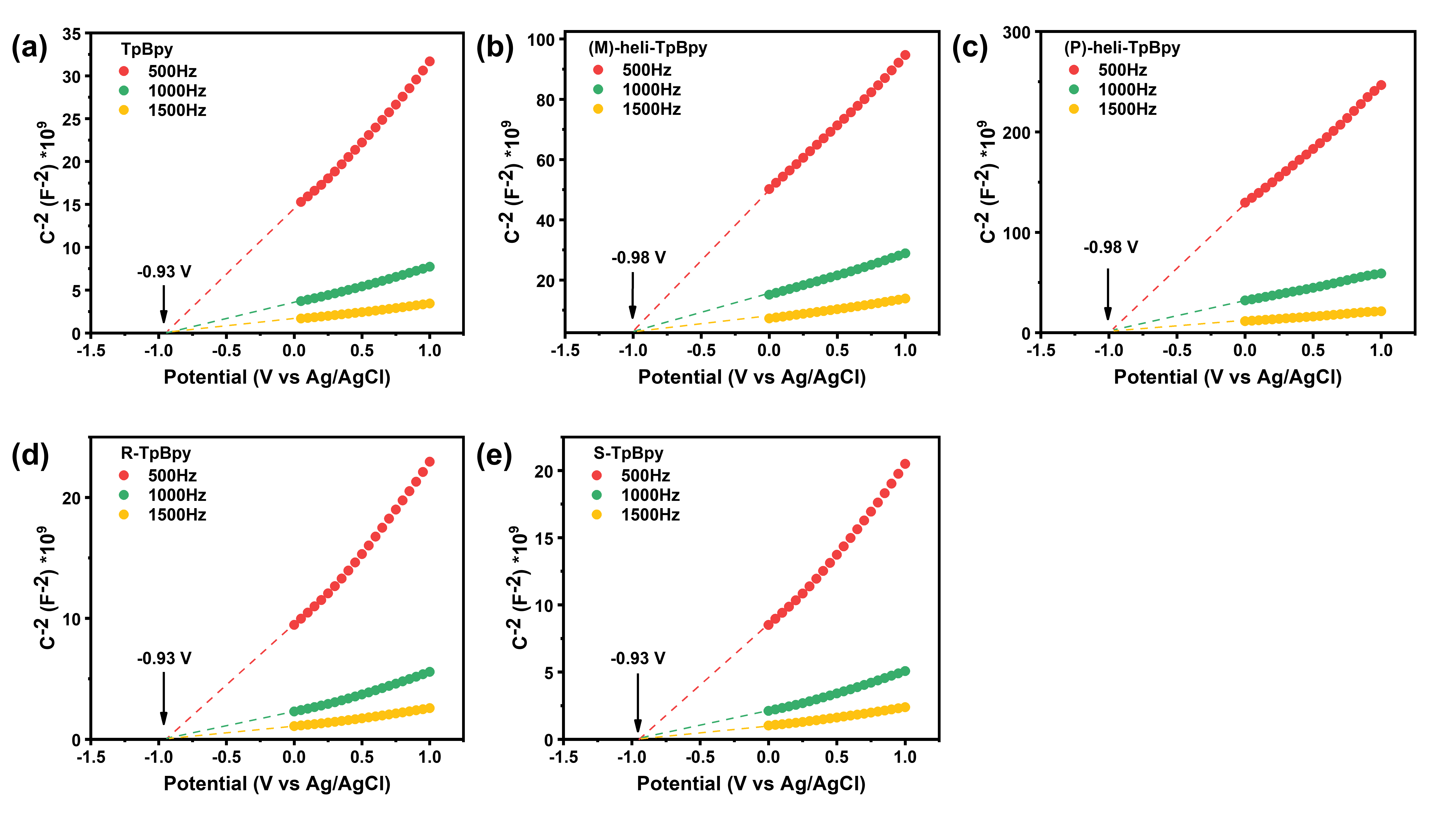


**Figure S22.** Mott-Schottky plots of (a)TpBpy, (b, c) helical TpBpy, and (d, e) chiral TpBpy.


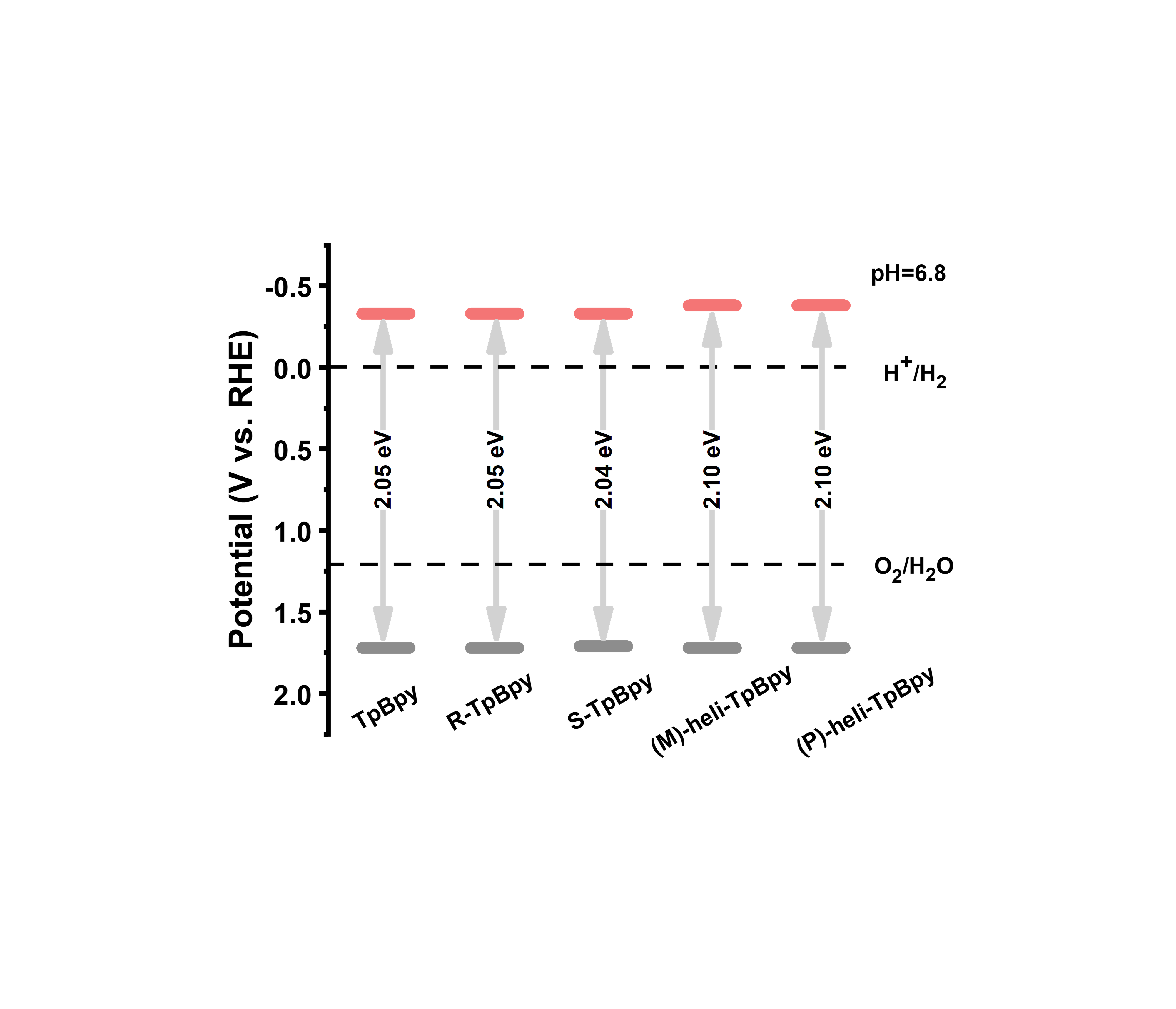


**Figure S23.** Energy band structures of the achiral TpBpy, chiral TpBpy and helical TpBpy.


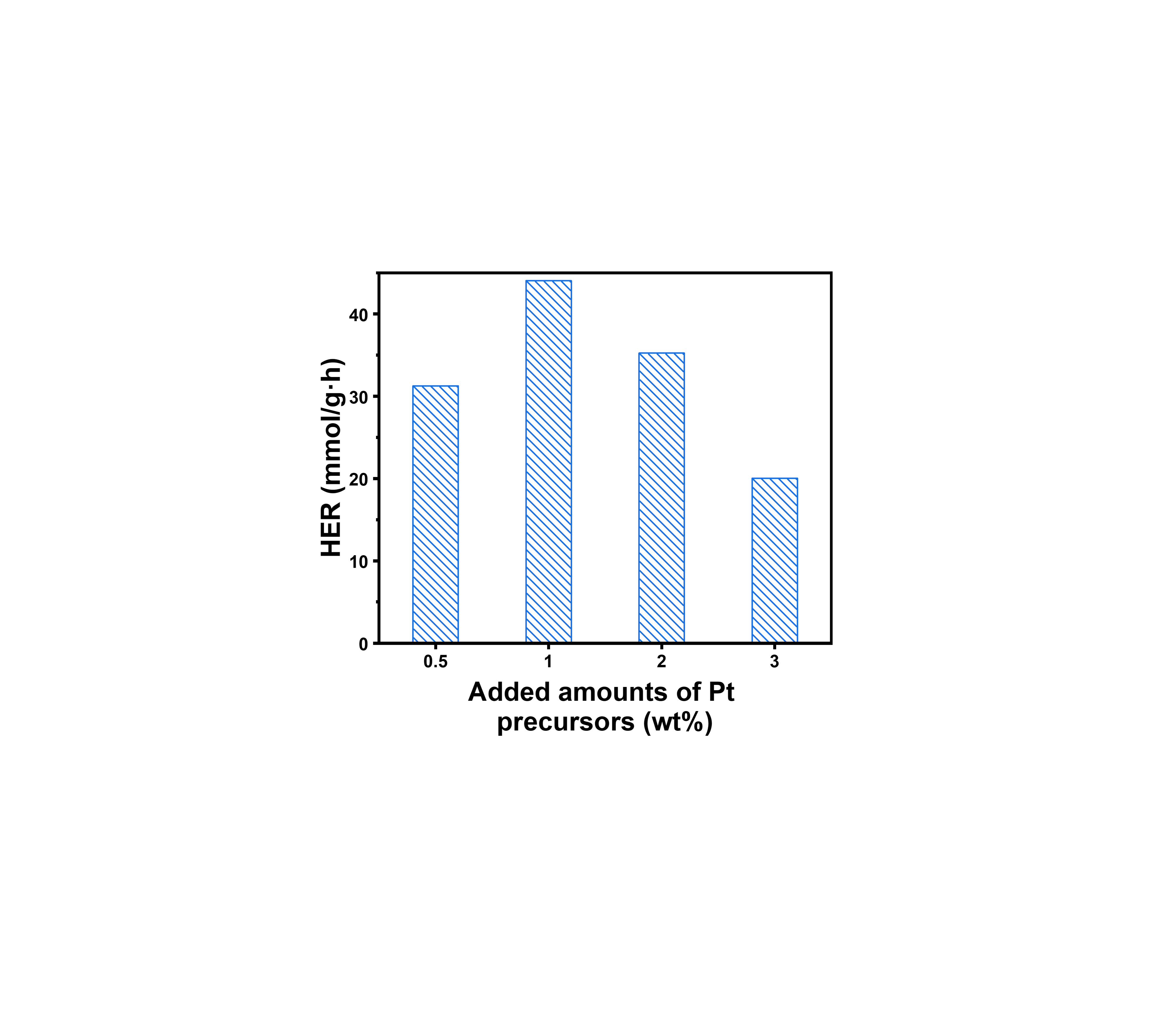


**Figure S24.** Average H_2_ evolution rates (HERs) for the Pt-loaded (*M*)-heli-TpBpy using different added contents of Pt precursor.


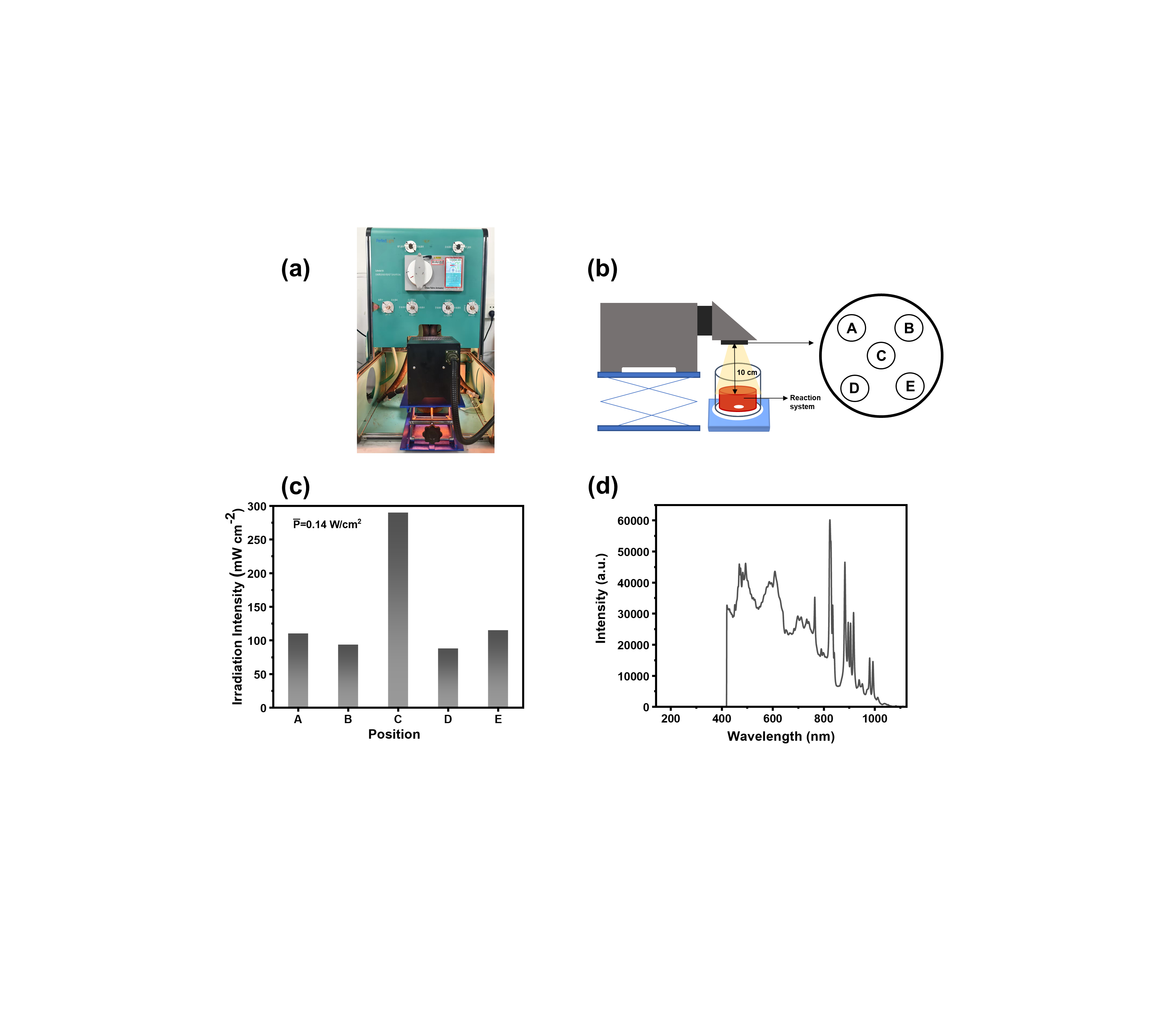


**Figure S25.** (a) Photograph of the device used in the photocatalytic H_2_ evolution tests. (b) Scheme of the reaction apparatus assembly and light irradiation condition. (c) The irradiation intensity measured for the position illustrated in Scheme b. (d) Light source spectra for the 300 W Xe lamp equipped with a cut-off filter (>420 nm).


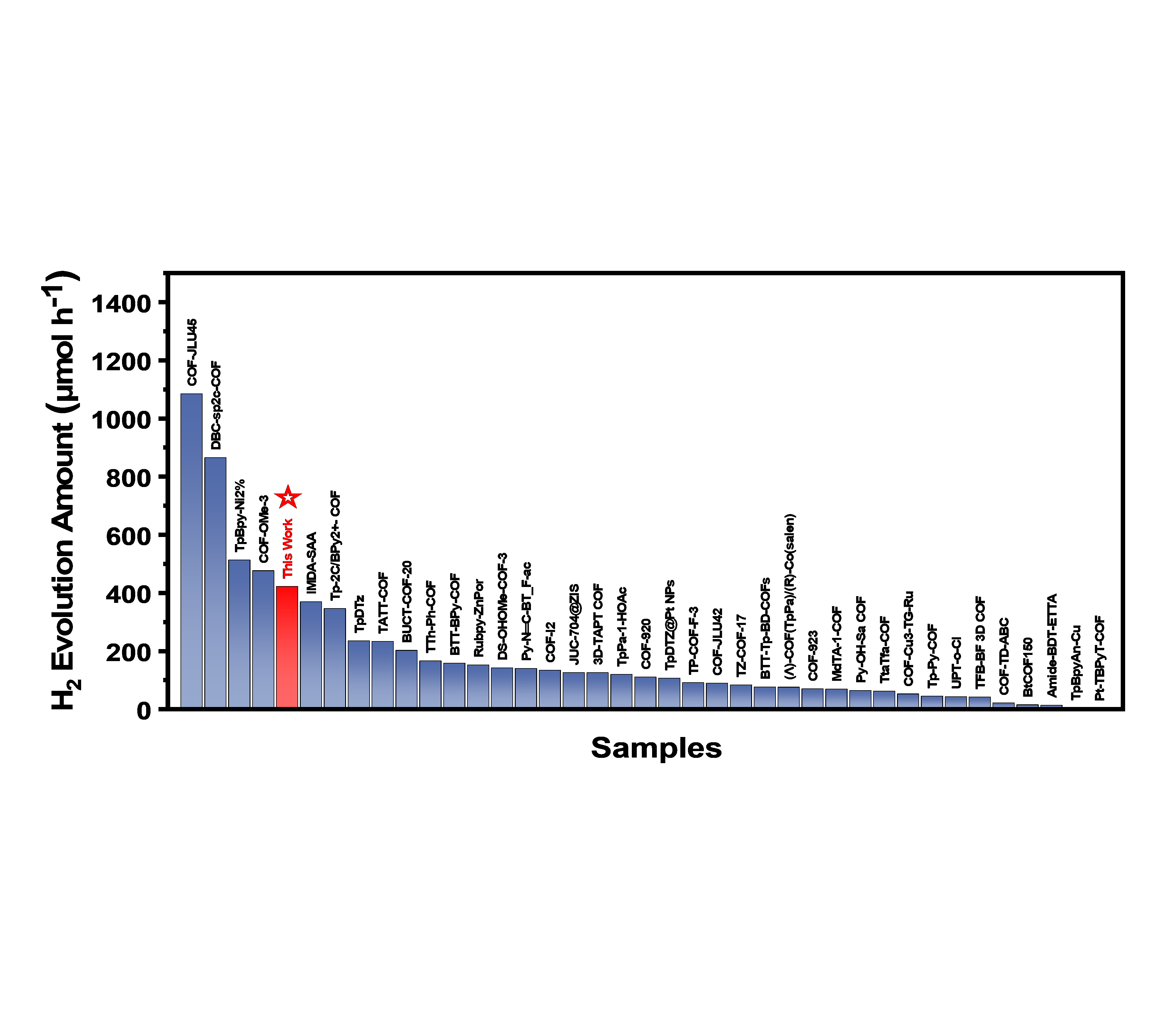


**Figure S26.** Comparing the H_2_ evolution amount for COF-based photocatalyst based on this study and previous reports.

**Table S2.** Comparison of photocatalytic H_2_ evolution performances with different COF-based photocatalysts.

| **Catalyst** | **Catalysts Amount (mg)** | **Co-catalyst** | **Sacrificial agent** | **HER (μmol g^-1^ h^-1^)** | **AQE (%)** | **H_2_ Evolution Amount**  **(μmol h^-1^)** | **Light Source** | **Ref** |
| --- | --- | --- | --- | --- | --- | --- | --- | --- |
| **(*M*)-heli-TpBpy** | **10** | **1 wt% Pt** | **Ascorbic acid** | **42210** | **7.18 (500 nm)** | **422.1** | **>420 nm (Xe, 300W)** | **This work** |
| COF-JLU45 | 5 | 1 wt% Pt | AA | 216900 | 12.90 (600 nm) | **1084.5** | >420 nm (Xe, 300W) | [4] |
| DBC-sp2c-COF | 5 | 0.8 wt% Pt | AA | 172900 | 14.91 (420 nm) | **864.5** | >420 nm (Xe, 300W) | [5] |
| TpBpy-Ni2% | 10 | 3 wt% Pt | AA | 51300 | 5.30  (475 nm) | **513** | >420 nm (Xe, 300W) | [6] |
| COF-OMe-3 | 20 | 3 wt% Pt | AA | 23800 | 9.62 (450 nm) | **476** | >420 nm (Xe, 300W) | [7] |
| IMDA-SAA | 5 | 1.5 wt% Pt | AA | 73800 | 5.76  (420 nm) | **369** | >420 nm (Xe, 300W) | [8] |
| Tp-2C/BPy^2+^- COF | 10 | 3 wt% Pt | AA | 34600 | 6.93 (420 nm) | **346** | >420 nm (Xe, 300W) | [9] |
| TpDTz | 10 | 3 wt% Pt | AA | 23511 | 15.58 (420 nm) | **235.11** | >420 nm (Xe, 300W) | [10] |
| TATT-COF | 10 | 3 wt% Pt | AA | 23300 | / | **233** | 320 nm < 𝜆 < 780 nm (Xe, 300W) | [11] |
| BUCT-COF-20 | 5 | 1.6 wt% Pt | AA | 40360 | 2.58 (450 nm) | **201.8** | >400 nm (Xe, 300W) | [12] |
| TTh-Ph-COF | 3 | 3 wt% Pt | AA | 55360 | 20.31 (420 nm) | **166.08** | >420 nm (Xe, 300W) | [13] |
| BTT-BPy-COF | 10 | 3.56 wt% Pt | AA | 15800 | 3.72  (500 nm) | **158** | >420 nm (Xe, 300W) | [14] |
| Rubpy-ZnPor | 5 | 1 wt% Pt | AA | 30338 | 9.68 (420 nm) | **151.69** | 380 nm < 𝜆 < 800 nm (Xe, 300W) | [15] |
| DS-OHOMe-COF-3 | 1 | 1 wt% Pt | AA | 141800 | 1.33 (420 nm) | **141.8** | >420 nm (Xe, 300W) | [16] |
| Py-N═C-BT_F-ac | 10 | 8 wt % Pt | AA | 14000 | 1.40  (420 nm) | **140** | >420 nm (Xe, 300W) | [17] |
| COF-I2 | 5 | 1 wt% Pt | AA | 26720 | 12.10 (420 nm) | **133.6** | >420 nm (Xe, 300W) | [18] |
| JUC-704@ZIS | 5 | 2 wt% Pt | AA | 25080 | / | **125.4** | >420 nm (LED, 10W) | [19] |
| 3D-TAPT COF | 4 | 5 wt% Pt | AA | 31300 | 2.10 (420 nm) | **125.2** | >420 nm (Xe, 300W) | [20] |
| TpPa-1-HOAc | 20 | / | AA | 5950 | 4.10 (420 nm) | **119** | >420 nm (Xe, 300W) | [21] |
| COF-920 | 5 | 3 wt% Pt | AA | 22100 | 2.27 (520 nm) | **110.5** | >420 nm (Xe, 300W) | [22] |
| TpDTZ@Pt NPs | 1 | 5 wt% Pt | SA | 106000 | / | **106** | 𝜆 = 525 nm (LED, 100 mW/cm^2^) | [23] |
| TP-COF-F-3 | 10 | 3 wt% Pt | TEOA | 9060 | 4.96 (420 nm) | **90.6** | >420 nm (Xe, 300W) | [24] |
| COF-JLU42 | 5 | 3 wt% Pt | AA | 17790 | 1.18  (450 nm) | **88.95** | 420 nm < 𝜆 < 780 nm (Xe, 300W) | [25] |
| TZ-COF-17 | 2.5 | 3 wt% Pt | AA | 33270 | 2.7  (450 nm) | **83.175** | >420 nm (Xe, 300W) | [26] |
| BTT-Tp-BD-COFs | 10 | 3 wt% Pt | AA | 7620 | 0.48 (450 nm) | **76.2** | >420 nm (Xe, 300W) | [27] |
| (Λ)-COF(TpPa)/(R)-Co(salen) | 13.21 | / | AA | 5700 | 0.48  (475 nm) | **75.297** | >420 nm (Xe, 300W) | [28] |
| COF-923 | 5 | 3 wt% Pt | TEOA | 13900 | 0.68 (450 nm) | **69.5** | AM 1.5 | [29] |
| MdTA-1-COF | 10 | 3 wt% Pt | AA | 6920 | 0.17 (420 nm) | **69.2** | >420 nm (Xe, 300W) | [30] |
| Py-OH-Sa COF | 1 | 1 wt% Pt | AA | 64210 | 7.01  (420 nm) | **64.21** | Xe, 300 W | [31] |
| TtaTfa-COF | 3 | 8 wt% Pt | AA | 20700 | 1.43  (450 nm) | **62.1** | >420 nm (Xe, 300W) | [32] |
| COF-Cu3-TG-Ru | 5 | / | TEA | 10470 | 0.62 (420 nm) | **52.35** | 420 nm < 𝜆 < 780 nm (Xe, 300W) | [33] |
| Tp-Py-COF | 2 | / | AA | 22450 | 3.30  (420 nm) | **44.9** | >420 nm (Xe, 300W) | [34] |
| UPT-*o*-Cl | 3 | Pt | SA | 14210 | 13.16 (400 nm) | **42.63** | >420 nm (Xe, 300W) | [35] |
| TFB-BF 3D COF | 2 | 1.02 wt% Pd | AA | 21040 | 8.7  (420 nm) | **42.08** | AM 1.5 | [36] |
| COF-TD-ABC | 2 | 2 wt% Pt | AA | 10920 | 8.79 (420 nm) | **21.84** | >420 nm (Xe, 300W) | [37] |
| BtCOF150 | 20 | 1 wt% Pt | TEOA | 750 | 0.2  (420 nm) | **15** | >400 nm (Xe, 300W) | [38] |
| Amide-BDT-ETTA | 5 | 1 wt% Pt | AA | 2600 | / | **13** | >420 nm (Xe, 300W) | [39] |
| TpBpyAn-Cu | 0.25 | / | AA | 12300 | / | **3.075** | >420 nm (Xe, 300W) | [40] |
| Pt-TBPyT-COF | 2 | 5 wt% Pt | SA | 469.8 | 8.91 (450 nm) | **0.9396** | >420 nm (Xe, 300W) | [41] |

Note: /=none or not described, AA=ascorbicacid, TEOA=triethanolamine, TEA=triethylamine, and SA=sodiumascorbate.


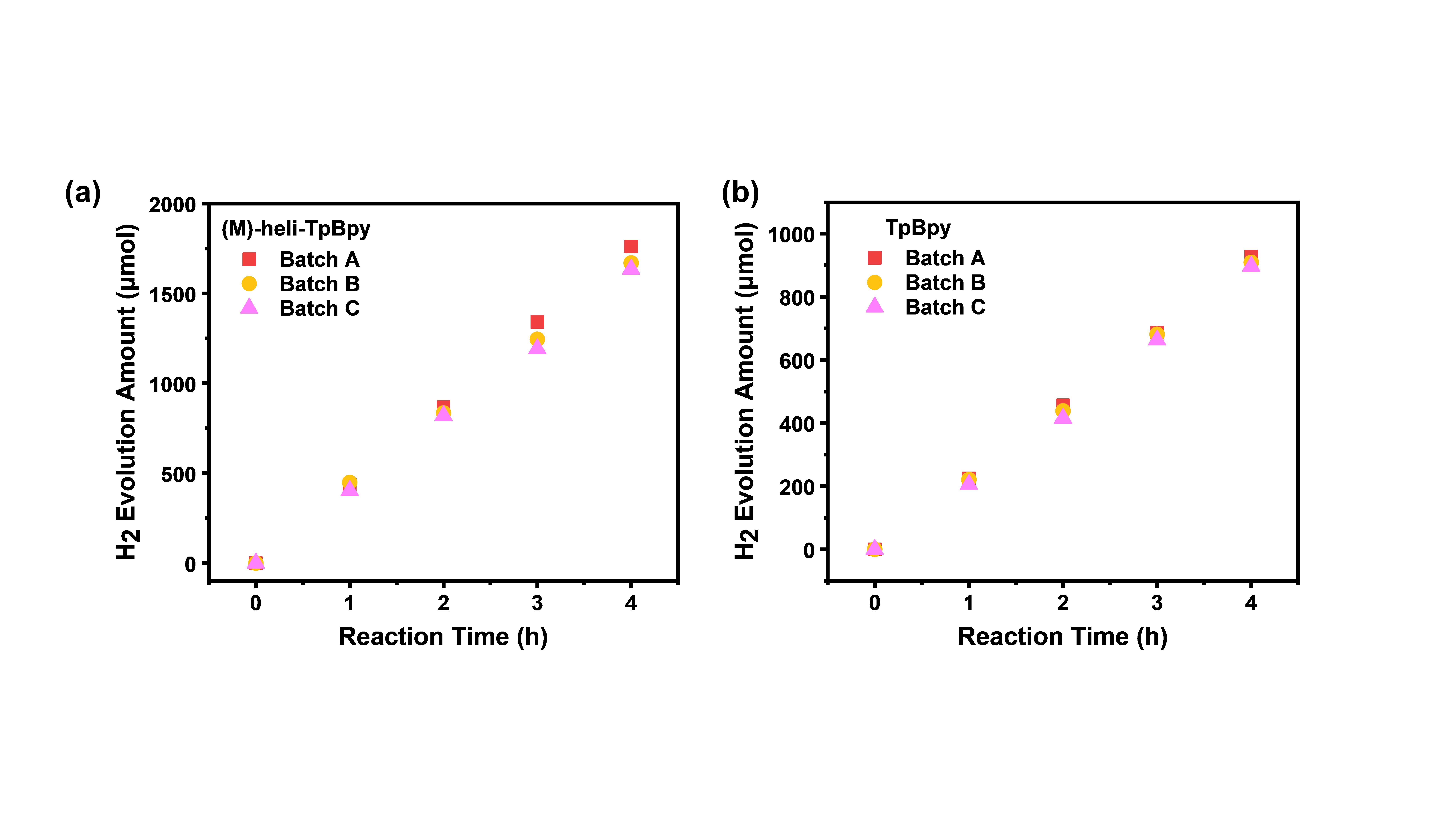


**Figure S27.** Repetitive test for photocatalytic H_2_ evolution by using (a) (*M*)-heli-TpBpy and (b) TpBpy from three different batches.


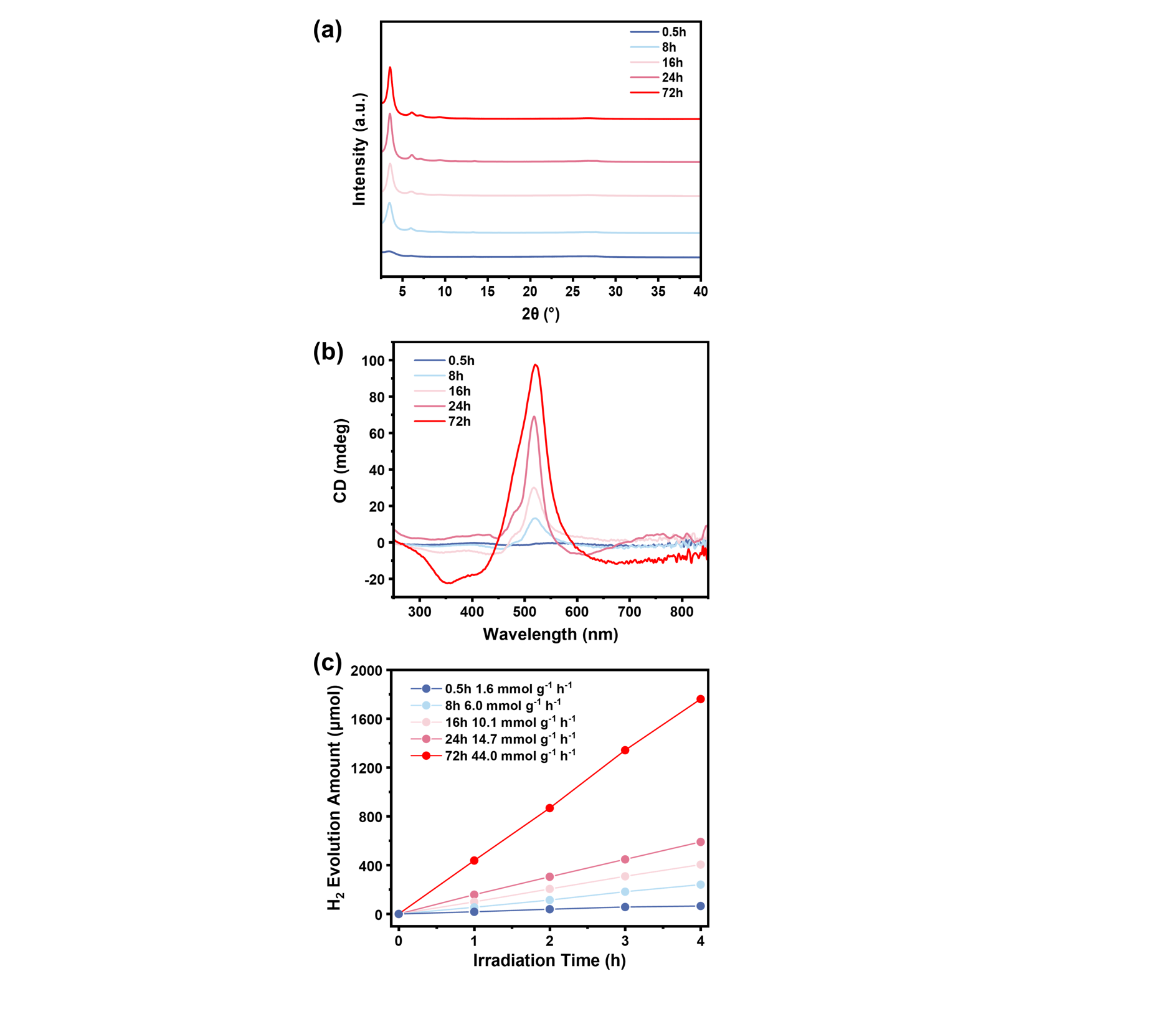


**Figure S28.** (a) PXRD patterns, (b) CD sprctra and (c) Time-dependent hydrogen evolution curves for (*M*)-heli-TpBpy synthesized by different solvothermal reaction time.


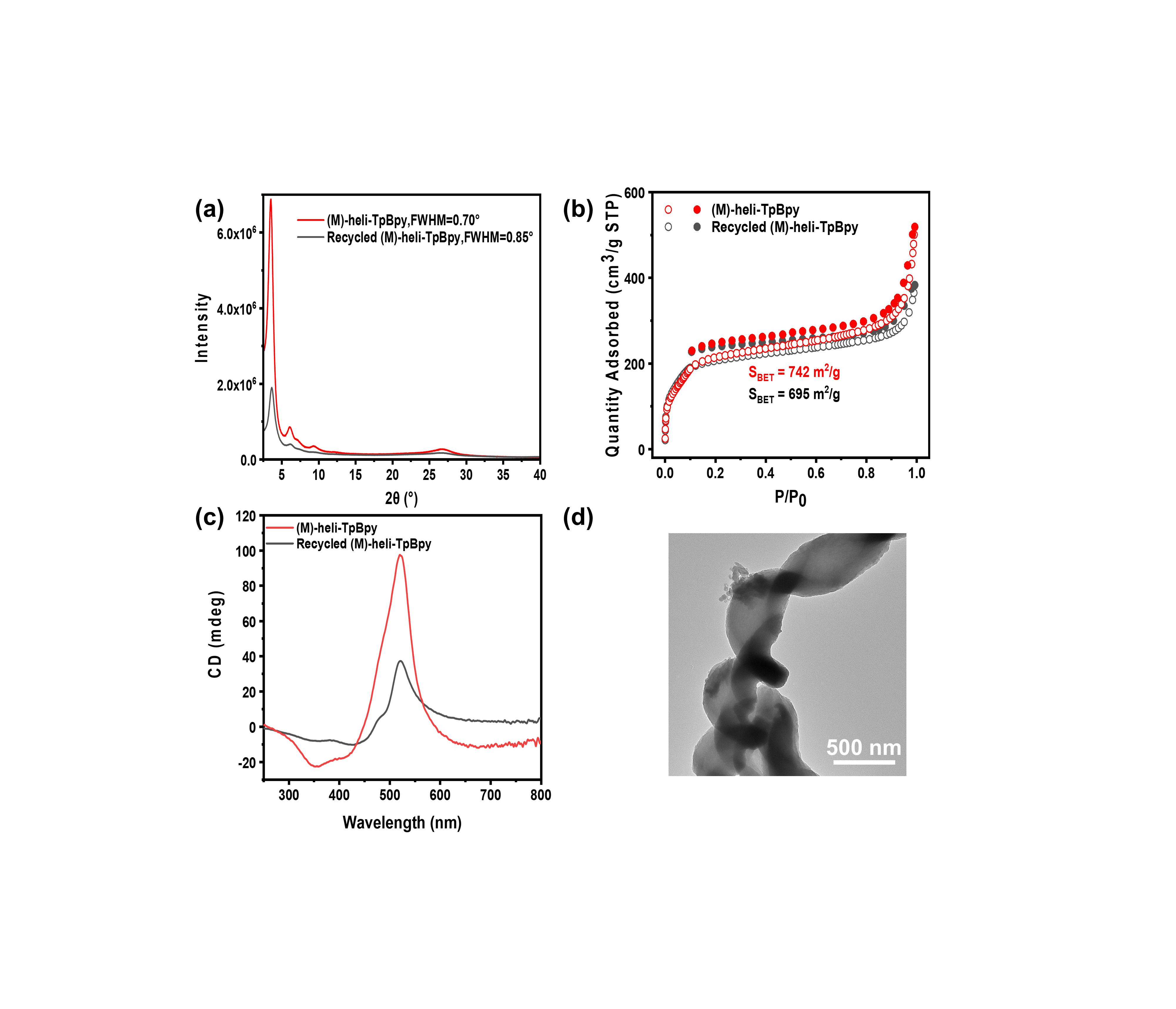


**Figure S29**. (a) PXRD patterns. (b) Nitrogen adsorption (open circle) and desorption (solid circle) isotherm profiles. (c) CD spectrum of (*M*)-heli-TpBpy before (red) and after irradiation (black). (d) TEM image of (*M*)-heli-TpBpy after irradiation.


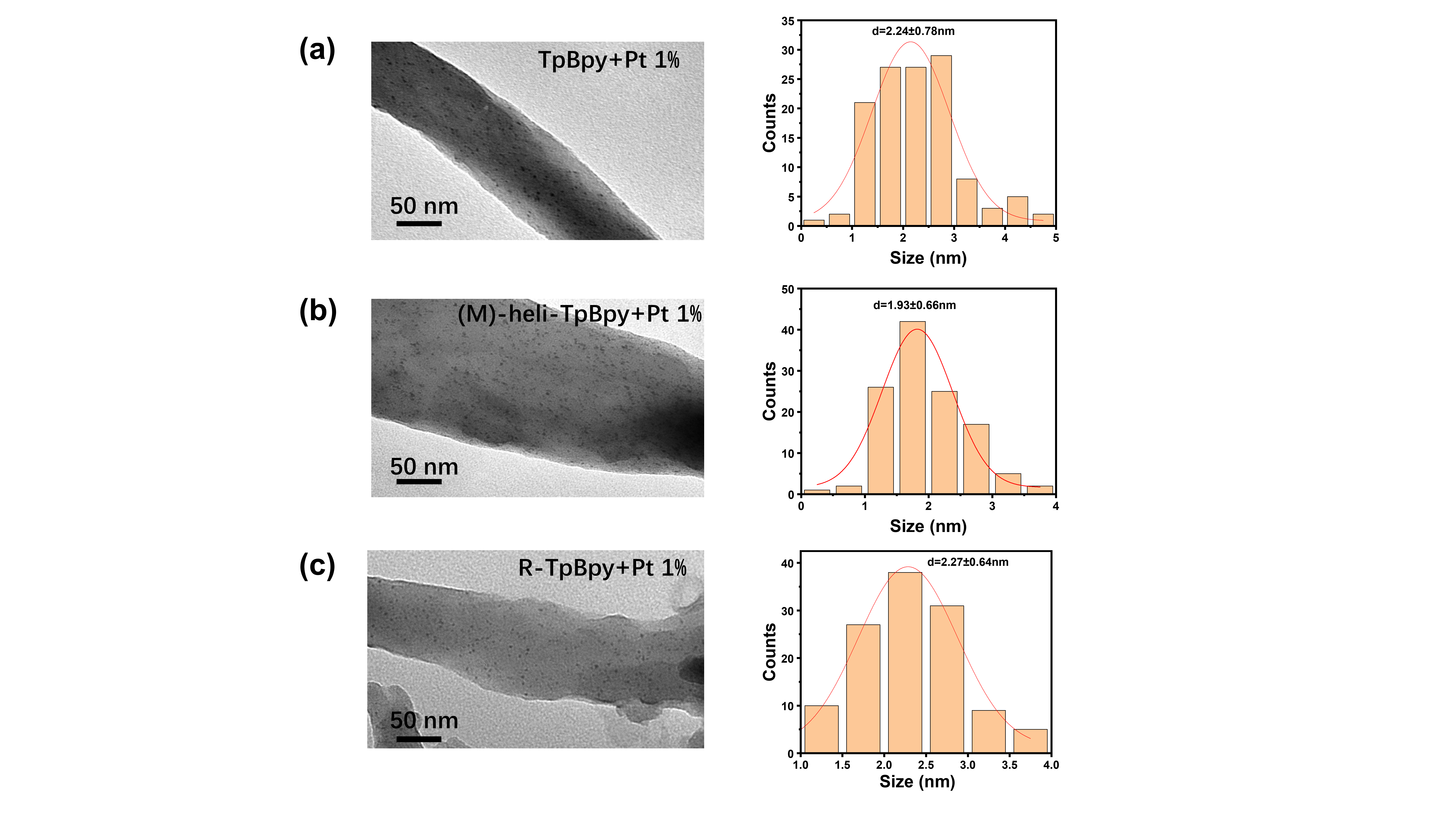


**Figure S30.** TEM images and statistical size distributions of the photo-deposited Pt nanoparticles onto (a) TpBpy, (b) (*M*)-heli-TpBpy and (c) *R*-TpBpy.


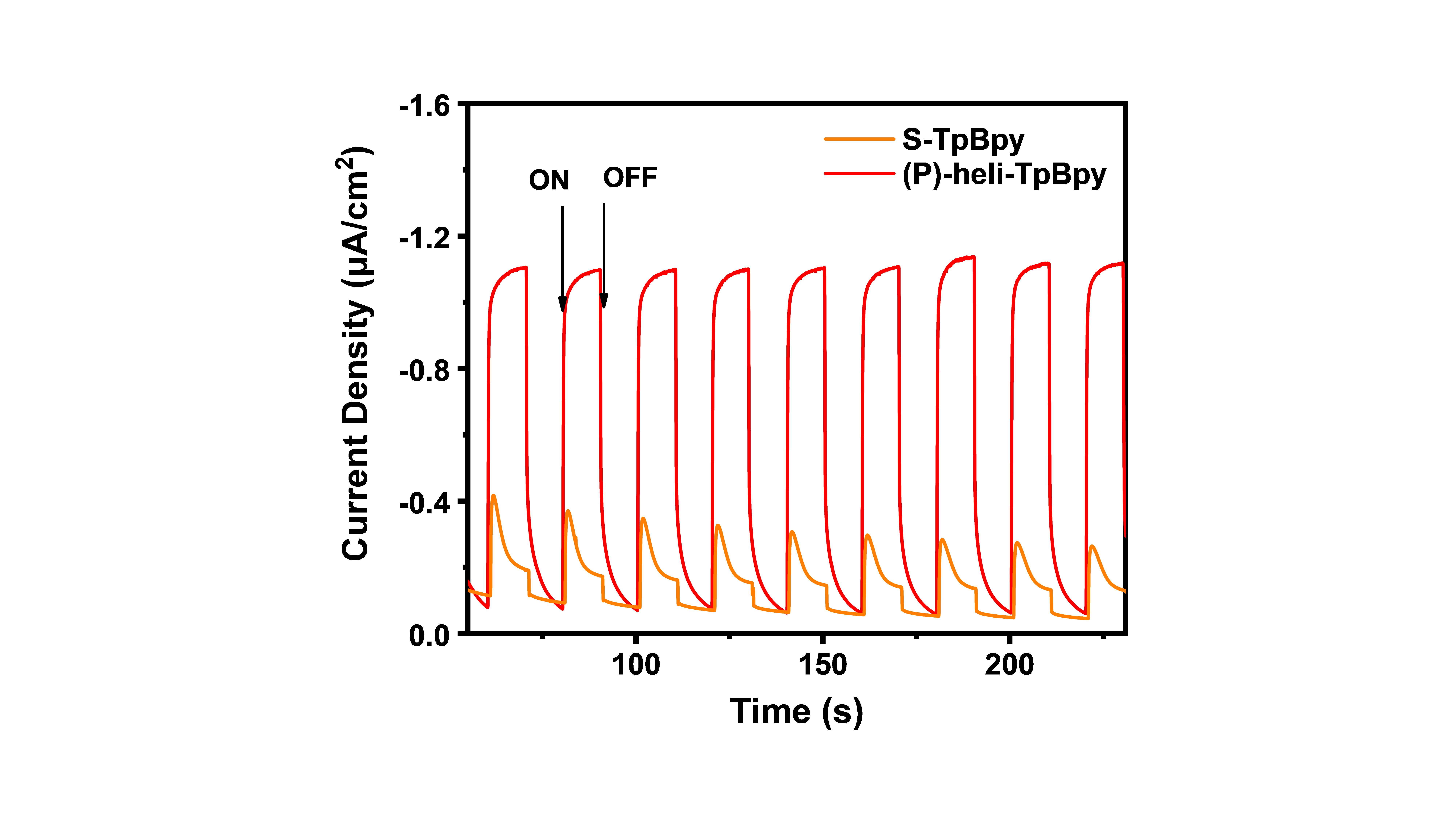


**Figure S31.** Transient photocurrent response of (*P*)-heli-TpBpy and *S*-TpBpy.


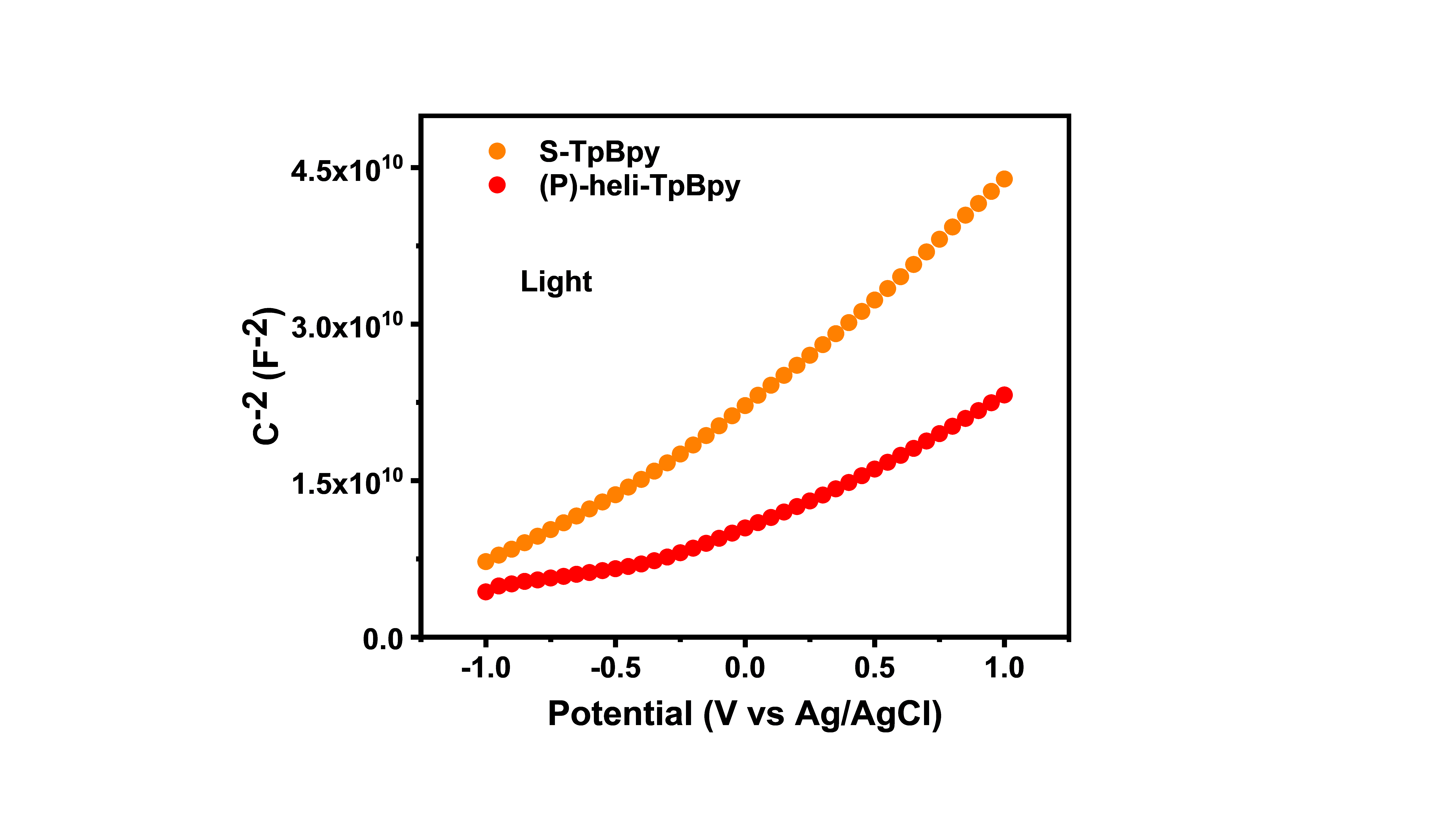


**Figure S32.** Mott–Schottky plots of COFs under visible light irradiation (λ > 420 nm, 300 W Xe lamp) of (*P*)-heli-TpBpy and *S*-TpBpy.


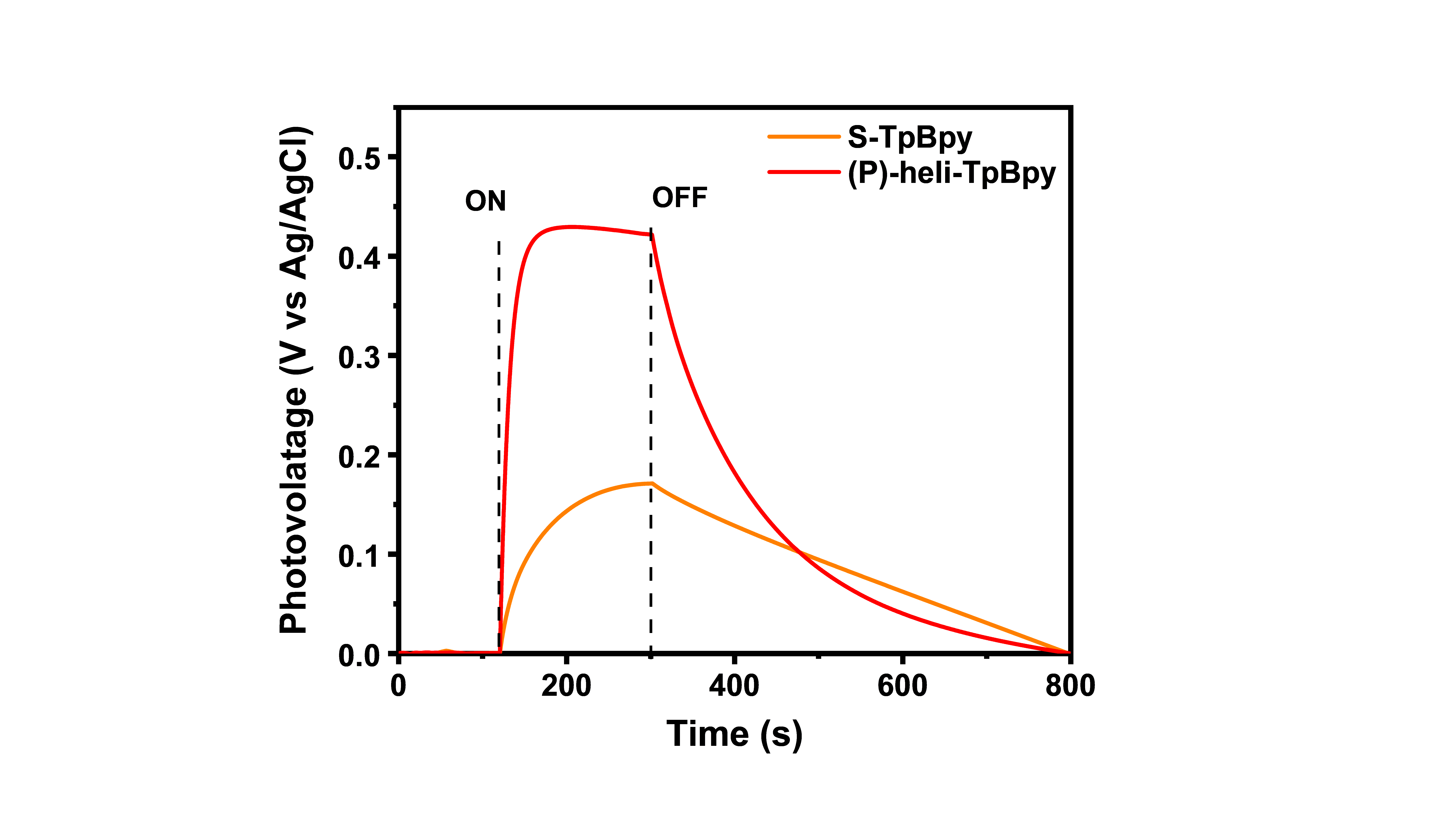


**Figure S33.** Transient open-circuit voltage decay (OCVD) measurements of (*P*)-heli-TpBpy and *S*-TpBpy.


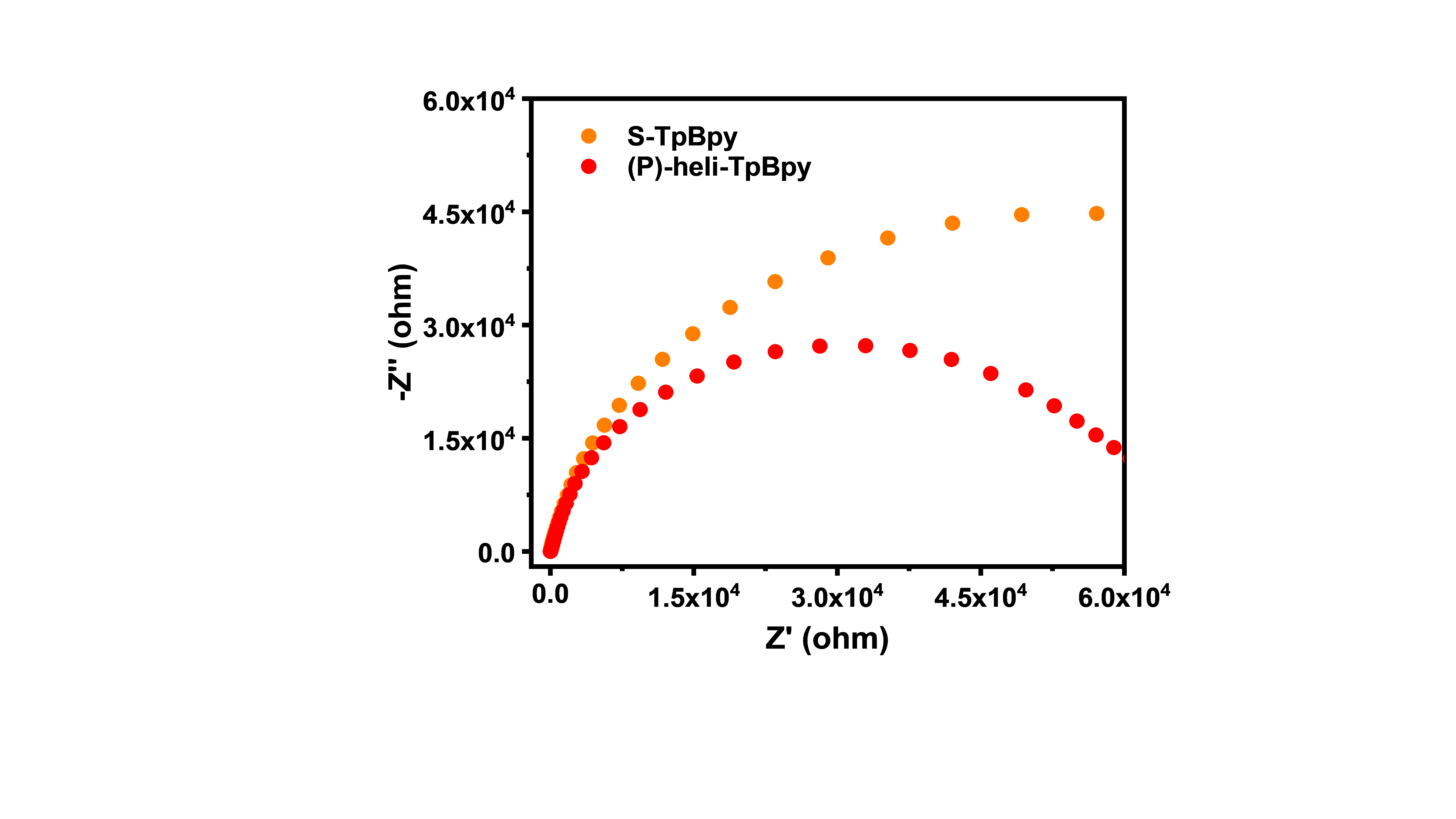


**Figure S34.** Nyquist plots of (*P*)-heli-TpBpy and *S*-TpBpy.


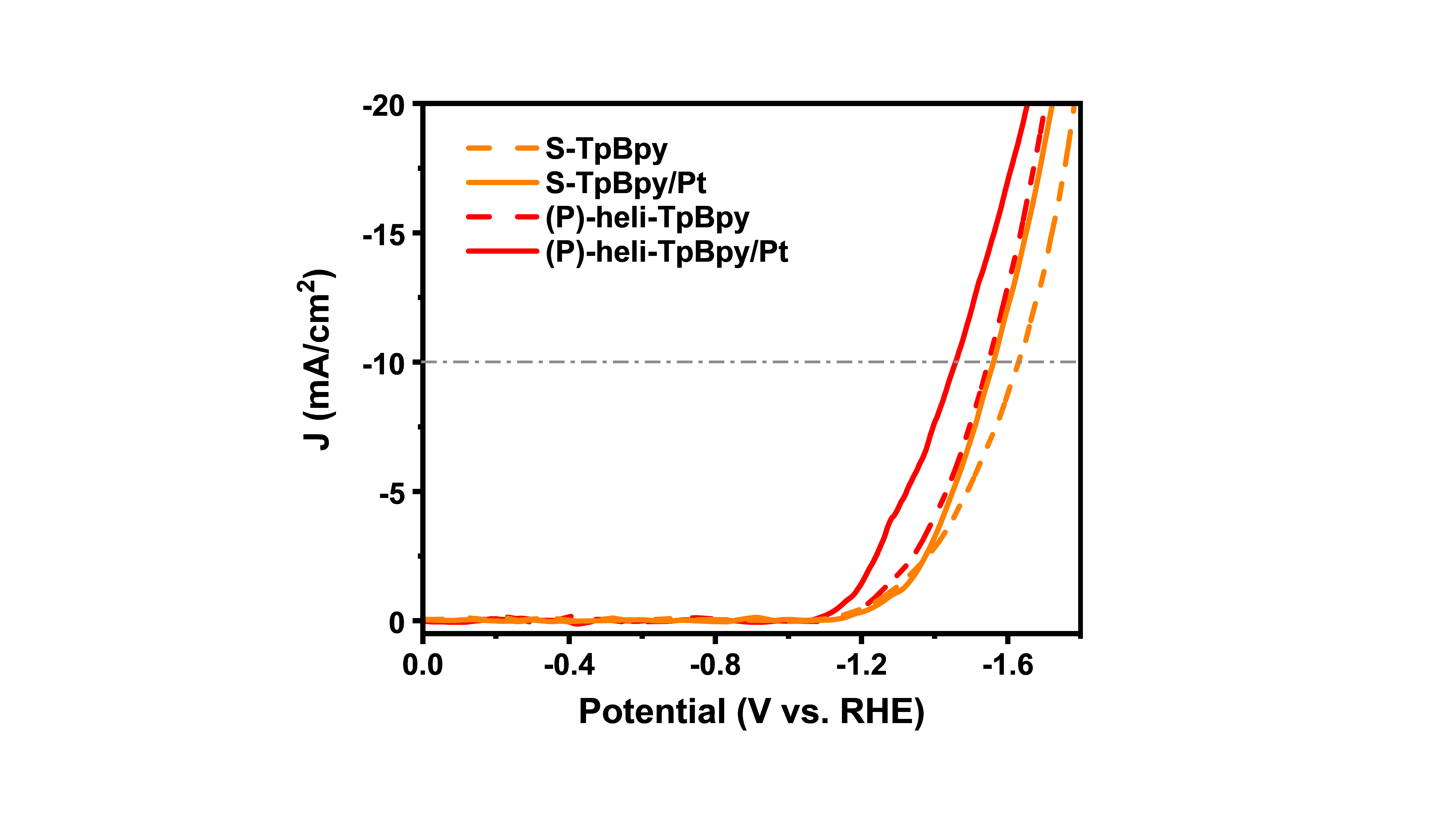


**Figure S35.** LSV plots of (*P*)-heli-TpBpy and *S*-TpBpy before and after Pt loading.


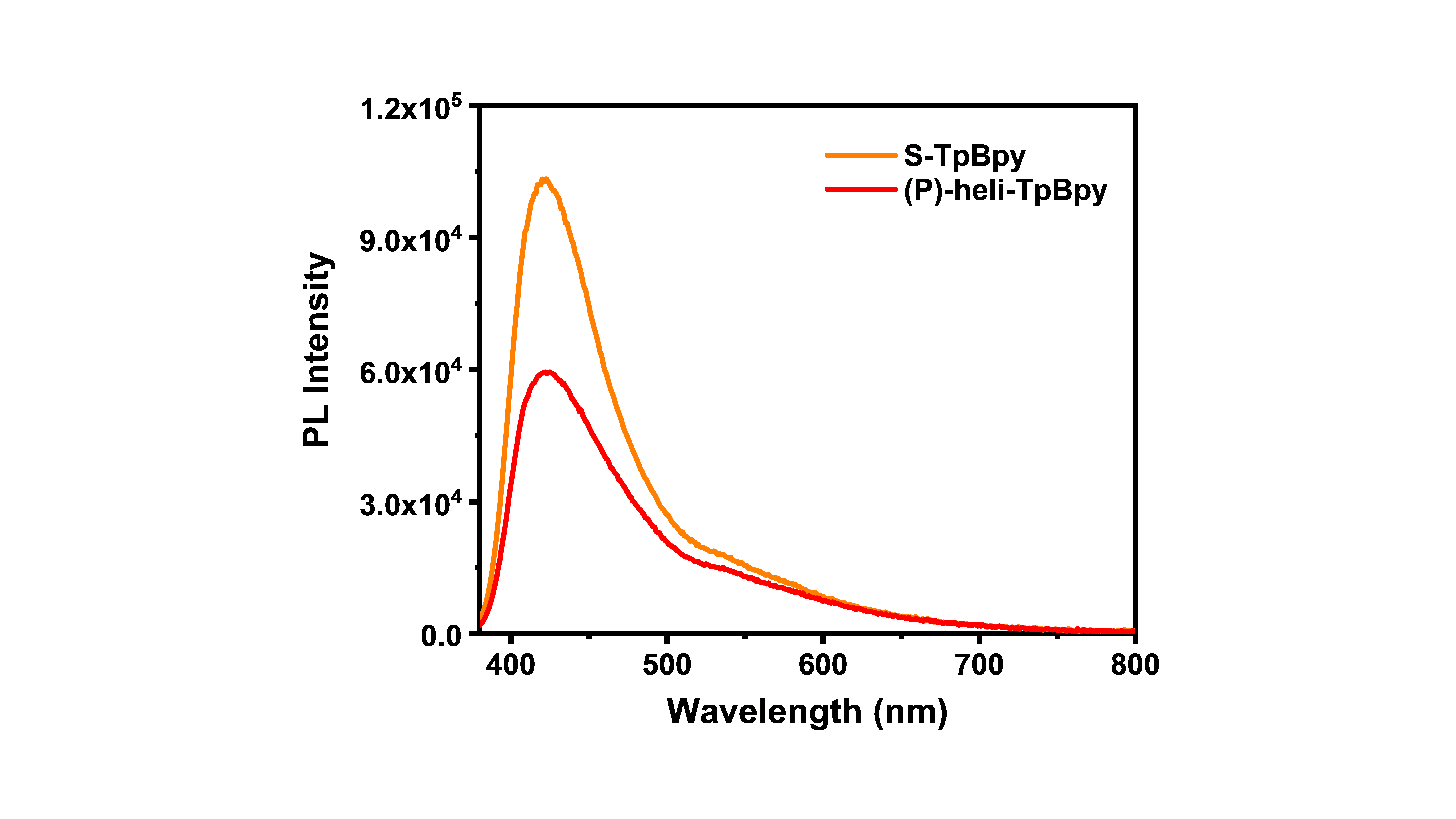


**Figure S36.** PL spectra of (*P*)-heli-TpBpy and *S*-TpBpy.


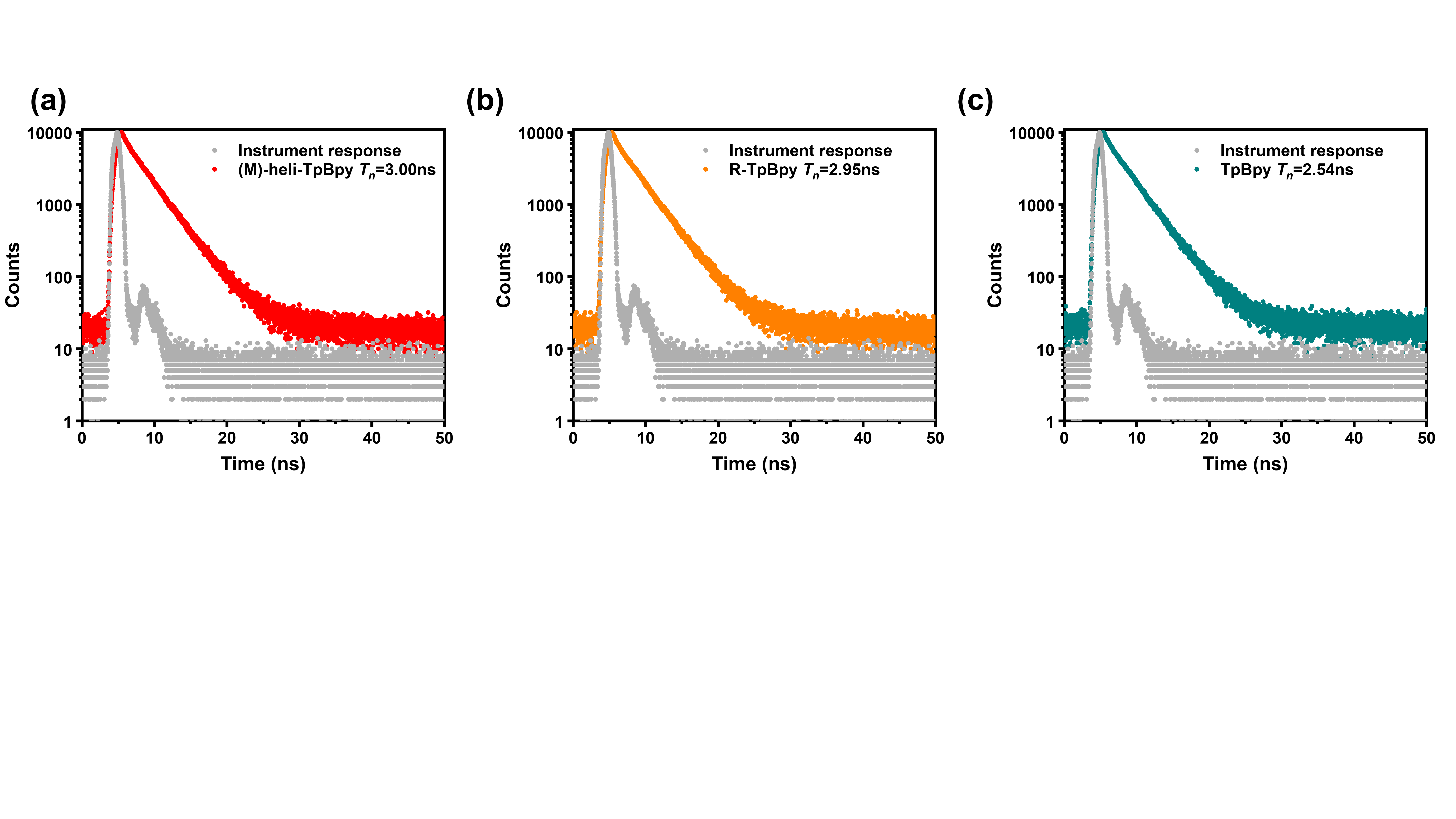


**Figure S37**. Transient decay profile in deionized water of (a) (*M*)-heli-TpBpy, (b) *R*-TpBpy and (c) TpBpy.


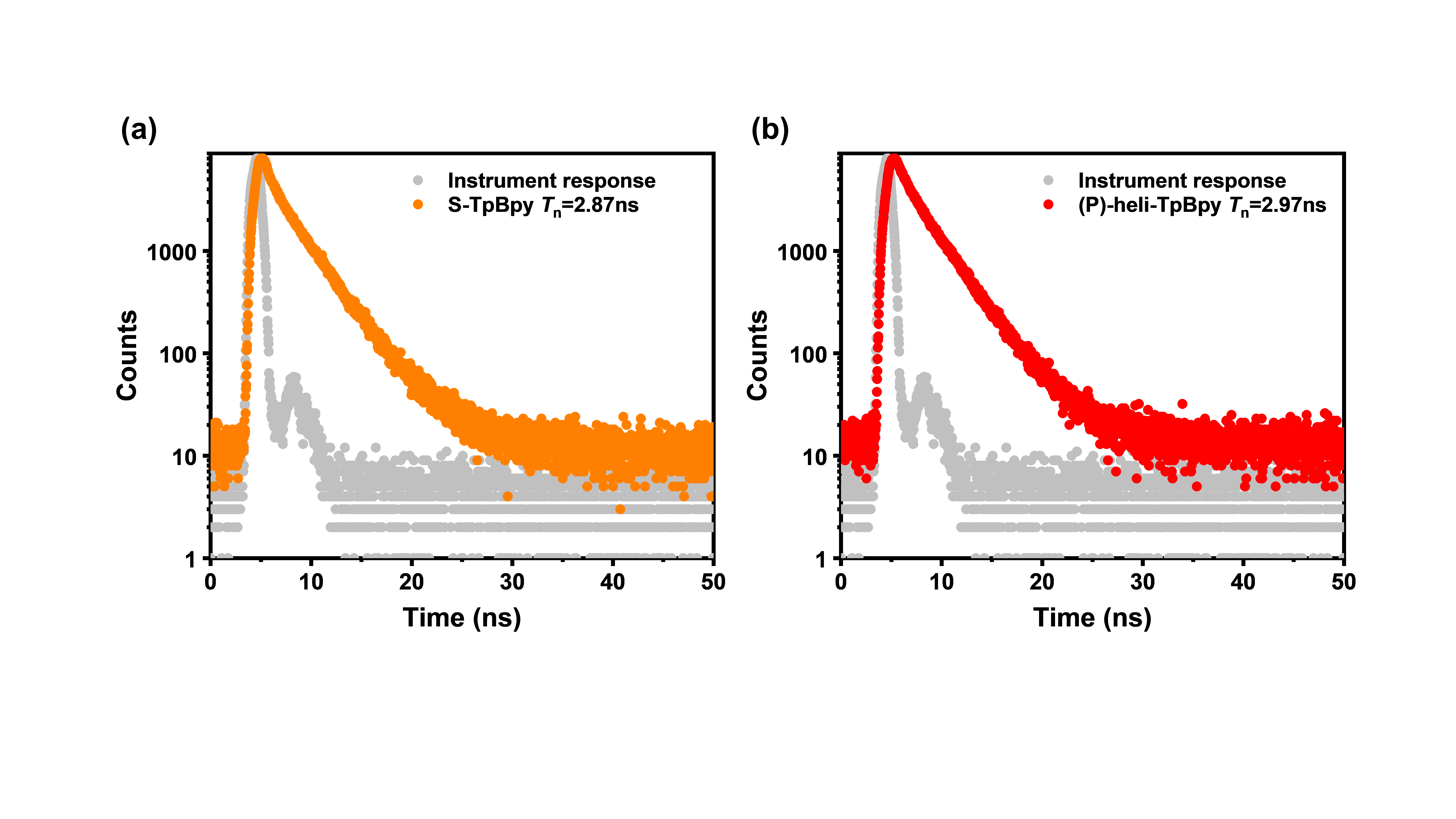


**Figure S38.** Transient decay profile in deionized water of (a) (*P*)-heli-TpBpy and (b) *S*-TpBpy.

**
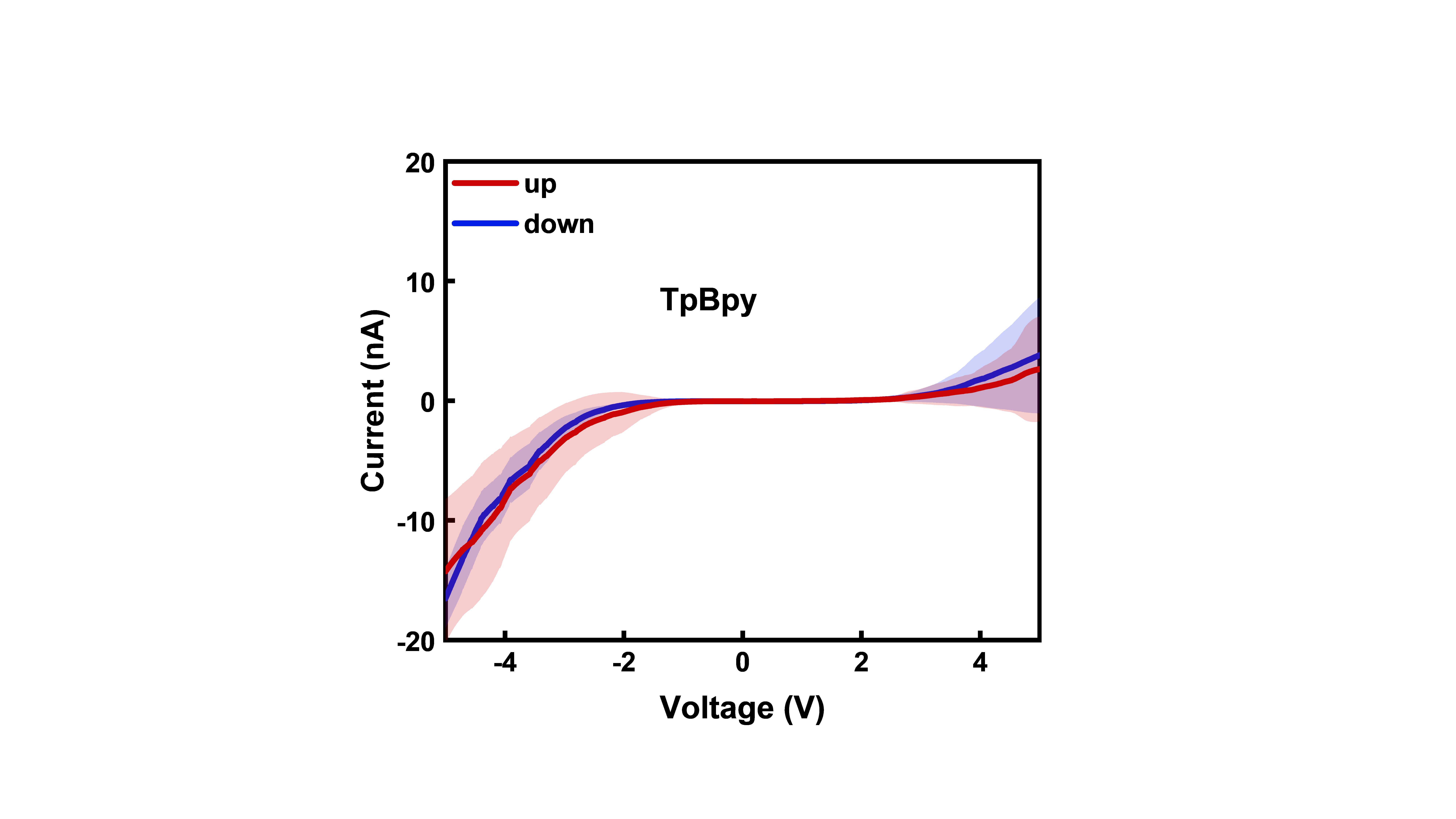
**

**Figure S39.** Average I-V curves for TpBpy, measured by mc-AFM.


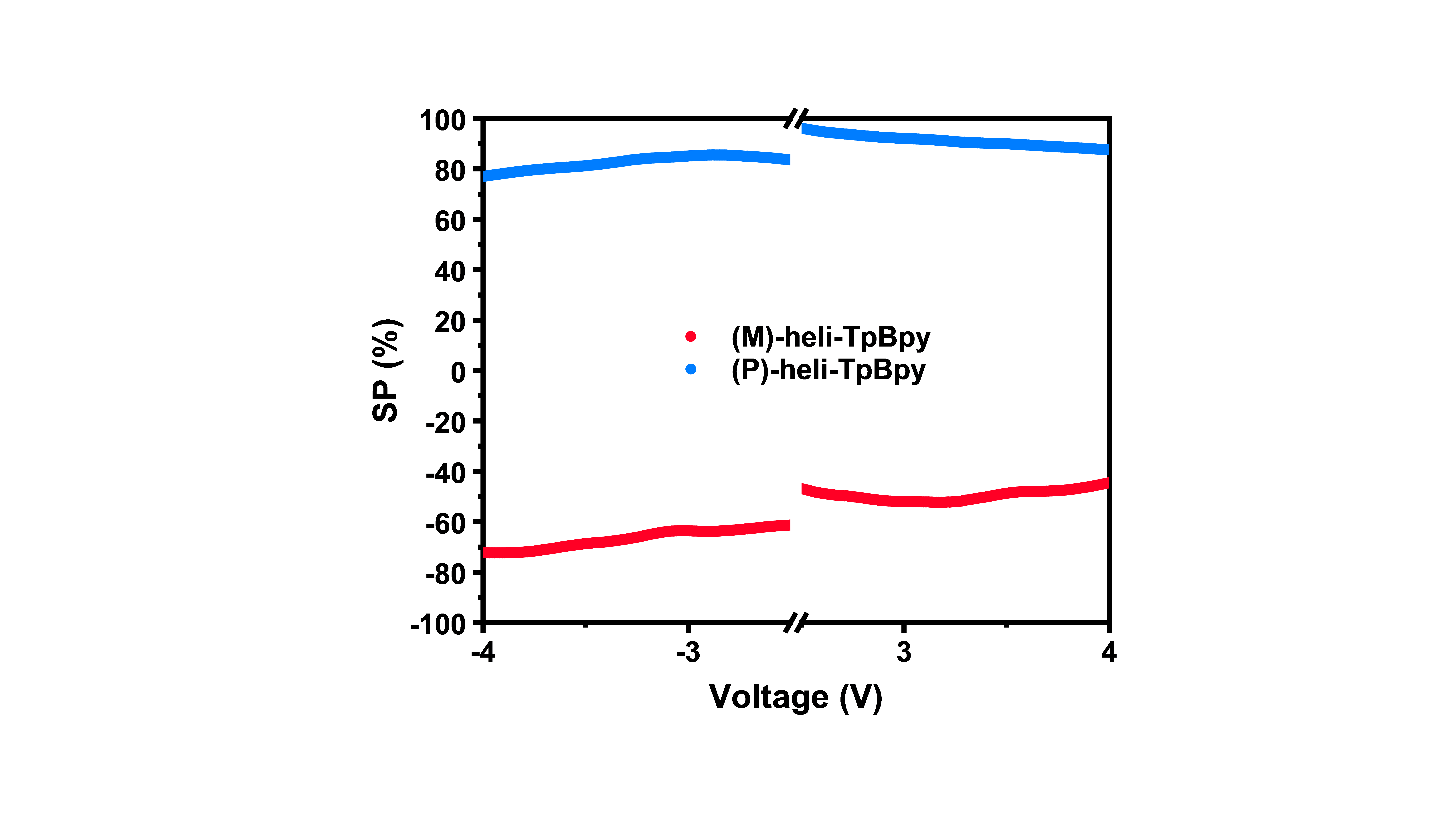


**Figure S40.** The spin polarized curves of (*P/M*)-heli-TpBpy.

**Table S3.** Comparison of the spin polarization (SP) of helical TpBpy with the reported chiral COFs.

| **Samples** | **Loaded object** | **\|SP\| (%)** | **Ref** |
| --- | --- | --- | --- |
| **(*M*)-heli-TpBpy** | **None** | **74** | **This work** |
| **(*P*)-heli-TpBpy** | **None** | **88** | **This work** |
| (*R*)-37 | None | 46 | [42] |
| (*S*)-37 | None | 50 |  |
| (*R*)-38 | None | 55 |  |
| (*S*)-38 | None | 59 |  |
| (*R*)-13-OEt-h | None | 48 | [43] |
| (*S*)-13-OEt-h | None | 51 |  |
| 25 systems | None | 57 | [44] |
| 26 systems | None | 52 |  |
| 27 systems | None | 41 |  |
| (*R*)-CCOF-9-Co | Co | 88 | [45] |
| (*S*)-CCOF-9-Co | Co | 94 |  |
| S-py-Co-COF | Co | 49 | [46] |
| L-His-Co-COF | Co | 51 |  |
| L-MA-Co-COF | Co | 60 |  |
| L-Ser-Co-COF | Co | 70 |  |
| L-Ala-Co-COF | Co | 72 |  |


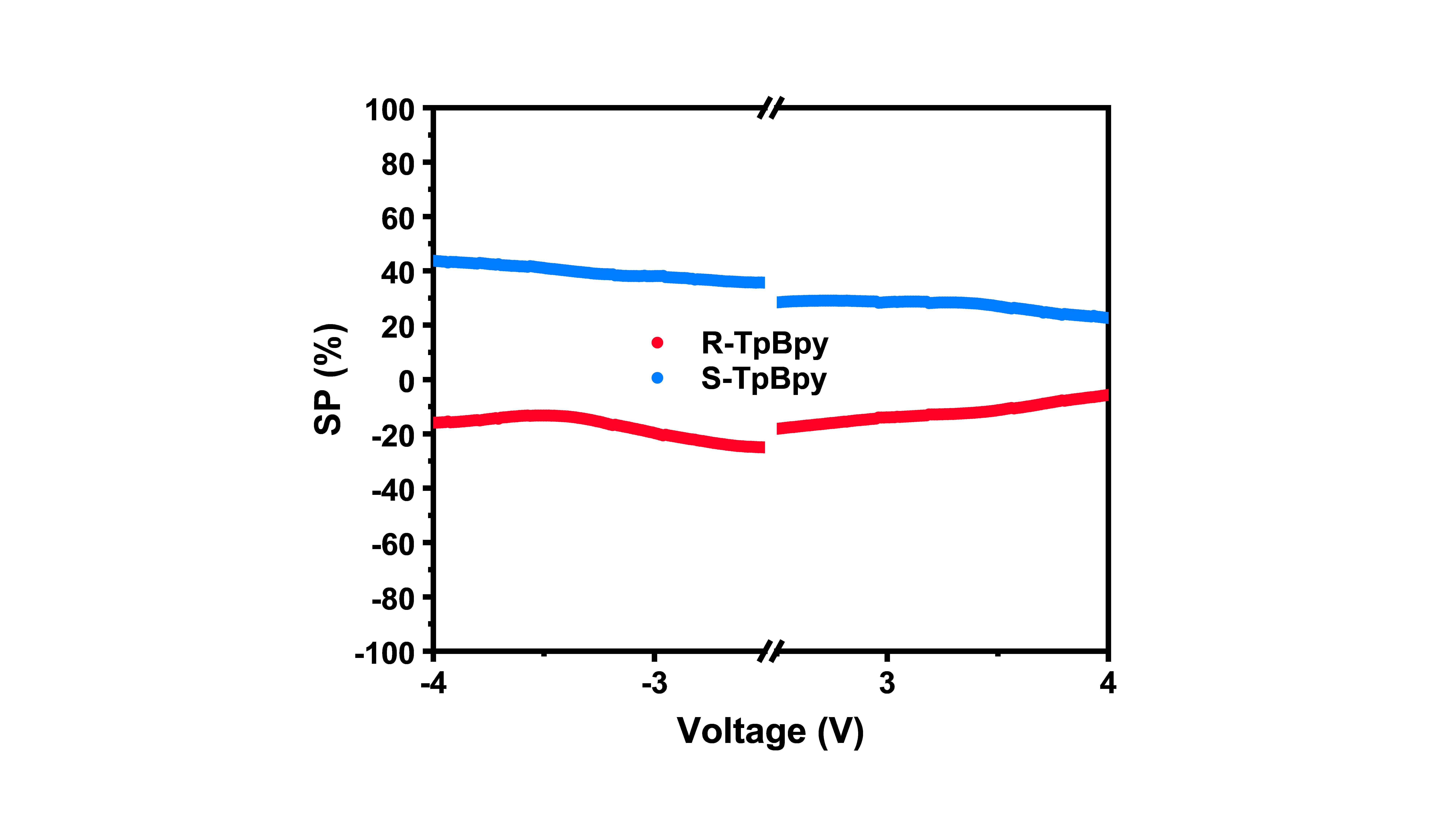


**Figure S41.** The spin polarized curves of (*R/S*)-TpBpy.

**Reference**

1. S. Anantharaj, S. R. Ede, K. Karthick, S. S. Sankar, K. Sangeetha, P. E. Karthik, S. Kundu, "Precision and correctness in the evaluation of electrocatalytic water splitting: revisiting activity parameters with a critical assessment," *Energy & Environmental Science* 11, no.4 (2018): 744, <https://doi.org/10.1039/c7ee03457a>.

2. T. Lu, F. Chen, "Multiwfn: A multifunctional wavefunction analyzer," *Journal of Computational Chemistry* 33, no.5 (2012): 580, <https://doi.org/10.1002/jcc.22885>.

3. T. Bai, J. Ai, L. Liao, J. Luo, C. Song, Y. Duan, L. Han, S. Che, "Chiral Mesostructured NiO Films with Spin Polarisation," *Angewandte Chemie-International Edition* 60, no.17 (2021): 9421, <https://doi.org/10.1002/anie.202101069>.

4. S. Ma, Z. Li, Y. Hou, J. Li, Z. Zhang, T. Deng, G. Wu, R. Wang, S.-w. Yang, X. Liu, "Fully Conjugated Benzobisoxazole-Bridged Covalent Organic Frameworks for Boosting Photocatalytic Hydrogen Evolution," *Angewandte Chemie-International Edition* 64, no.19 (2025): <https://doi.org/10.1002/anie.202501869>.

5. J.-J. Yu, X. Huang, L.-Y. Wang, Y.-Z. Wu, Z.-W. Huang, L.-L. Su, N.-N. Wang, J.-P. Yu, W.-Q. Shi, "Topology-Templated Synthesis of Dibenzo g,p Chrysene-Based sp2 Carbon-Linked Covalent Organic Frameworks with Kagome Lattice for Enhanced Photocatalytic Hydrogen Evolution," *Advanced Materials* (2025): <https://doi.org/10.1002/adma.202504808>.

6. H. Zhang, Z. Lin, P. Kidkhunthod, J. Guo, "Stable Immobilization of Nickel Ions on Covalent Organic Frameworks for Panchromatic Photocatalytic Hydrogen Evolution," *Angewandte Chemie International Edition* 62, no.21 (2023): <https://doi.org/10.1002/anie.202217527>.

7. Z. Luo, S. Zhu, H. Xue, W. Yang, F. Zhang, F. Xu, W. Lin, H. Wang, X. Chen, "Manipulating p‐π Resonance through Methoxy Group Engineering in Covalent Organic Frameworks for an Efficient Photocatalytic Hydrogen Evolution," *Angewandte Chemie International Edition* 64, no.6 (2025): <https://doi.org/10.1002/anie.202420217>.

8. Y. Shuang, Y. Zhang, H. Wang, L. Li, X. Hao, Z. Ma, S. Wang, J. Wang, F. Wang, X. Yang, P. Guo, F. Xu, H. Wang, Q. Ye, W. Liu, J. Jian, H. Wang, "Proton-Mediated Topological Interlayer Shift in 2D Covalent Organic Frameworks for Efficient Photocatalysis," *Advanced Materials* 37, no.24 (2025): <https://doi.org/10.1002/adma.202500468>.

9. Z. Mi, T. Zhou, W. Weng, J. Unruangsri, K. Hu, W. Yang, C. Wang, K. A. I. Zhang, J. Guo, "Covalent Organic Frameworks Enabling Site Isolation of Viologen‐Derived Electron‐Transfer Mediators for Stable Photocatalytic Hydrogen Evolution," *Angewandte Chemie International Edition* 60, no.17 (2021): 9642, <https://doi.org/10.1002/anie.202016618>.

10. H. Hou, K. Wu, X. Chen, X. Liu, Y. Zhao, "Thiazole-Bimodulated Covalent Organic Frameworks for Synergistic Water Harvesting and Photosplitting," *Journal of the American Chemical Society* 147, no.31 (2025): 27835, <https://doi.org/10.1021/jacs.5c06823>.

11. B. Yang, F. Jin, Z. Jin, "Ingenious strategy of a strong intermolecular force-linked donor-acceptor-donor system in COFs for efficient and improved photocatalytic hydrogen production," *Journal of Materials Chemistry A* 13, no.48 (2025): 42088, <https://doi.org/10.1039/d5ta06950e>.

12. Y. Wang, Z. Qiao, H. Li, R. Zhang, Z. Xiang, D. Cao, S. Wang, "Molecular Engineering for Modulating Photocatalytic Hydrogen Evolution of Fully Conjugated 3D Covalent Organic Frameworks," *Angewandte Chemie International Edition* 63, no.25 (2024): <https://doi.org/10.1002/anie.202404726>.

13. J. Chen, Q. Zhang, S. Liu, J. Qu, Y. Cai, X. Yang, C. Ming Li, J. Hu, H. B. Yang, "Dual-Channel Charge Transfer in Olefin-Linked Covalent Organic Frameworks for Efficient Photocatalytic Hydrogen Evolution from Seawater," *Advanced Functional Materials* (2025): <https://doi.org/10.1002/adfm.202530802>.

14. L. Dai, A. Dong, X. Meng, H. Liu, Y. Li, P. Li, B. Wang, "Enhancement of Visible‐Light‐Driven Hydrogen Evolution Activity of 2D π‐Conjugated Bipyridine‐Based Covalent Organic Frameworks via Post‐Protonation," *Angewandte Chemie International Edition* 62, no.15 (2023): <https://doi.org/10.1002/anie.202300224>.

15. M. Lu, S. B. Zhang, M. Y. Yang, Y. F. Liu, J. P. Liao, P. Huang, M. Zhang, S. L. Li, Z. M. Su, Y. Q. Lan, "Dual Photosensitizer Coupled Three‐Dimensional Metal‐Covalent Organic Frameworks for Efficient Photocatalytic Reactions," *Angewandte Chemie International Edition* 62, no.31 (2023): <https://doi.org/10.1002/anie.202307632>.

16. Y. Xu, Y. Lang, N. Sun, X. Ci, R. Tu, C. Yang, X. Fang, T. Liu, L. Yang, C.-C. Liu, W.-Q. Deng, Z. Li, "Systematic Regulation of Ternary Covalent Organic Frameworks for Boosted Photocatalytic Hydrogen Evolution," *ACS Catalysis* 15, no.22 (2025): 18976, <https://doi.org/10.1021/acscatal.5c05892>.

17. Z. Li, B. Cai, Q. Li, D. Zhang, Y. Liang, Y. Liu, Y. Jiao, A. Thomas, X. Zhao, "Modulating the Polarity of Imine Bonds in Donor-Acceptor Covalent Organic Frameworks for Enhanced Photocatalytic H<sub>2</sub> Production," *Angewandte Chemie-International Edition* 64, no.30 (2025): <https://doi.org/10.1002/anie.202509444>.

18. H. Li, Z. Luo, J. Han, Z. Yu, Q. Xue, Y. Zhao, J. Du, X. Zhou, F. Wang, "Nitrogen‐Shift‐Engineered Pt Single‐Atom/Cluster Synergy Boosts Covalent Organic Frameworks for Photocatalytic Hydrogen Evolution," *Angewandte Chemie International Edition* (2025): <https://doi.org/10.1002/anie.202524704>.

19. J. Zhang, X. Li, J. Chang, P. She, H. Li, S. Qiu, Q. Fang, "Dual Electric Fields in Cyclooctatetrathiophene-Based COF/ ZnIn2S4 Z-Scheme Heterojunction Boost Photocatalytic Seawater Hydrogen Evolution," *Angewandte Chemie-International Edition* (2025): <https://doi.org/10.1002/anie.202519752>.

20. H. Li, J. Fan, M. Ran, R. A. Borse, S. X. Lin, D. Yuan, "Design and Construction of D‐A‐Extended 3D Covalent–Organic Frameworks for Boosting Photocatalytic Hydrogen Evolution," *Angewandte Chemie International Edition* 64, no.24 (2025): <https://doi.org/10.1002/anie.202500937>.

21. P. Dong, Y. Zhang, L. Zhang, L. Zhang, B. Zhang, L. Wang, J. Zhang, "Structural Variation and Charge-Transfer Dynamics of Protonated β-Ketoenamine-Linked Covalent Organic Framework for Boosted Photocatalytic H2 Evolution," *ACS Catalysis* 15, no.21 (2025): 18138, <https://doi.org/10.1021/acscatal.5c05148>.

22. W. Dong, X. Yu, Z. Qin, Y. Chen, S. Ren, L. Li, "Synergistic Enhancement of Photocatalytic Hydrogen Evolution in Covalent Organic Frameworks via Isoreticular Design, Isomerism, and Protonation," *Angewandte Chemie-International Edition* 64, no.39 (2025): <https://doi.org/10.1002/anie.202511200>.

23. D. Reyes-Mesa, P. Sarró, M. F. Gusta, A. Jiménez-Solano, S. Das, B. P. Biswal, H. A. Vignolo-González, L. Velasco-Garcia, A. Llobet, N. G. Bastús, V. Puntes, A. Vallribera, R. Pleixats, A. Granados, B. V. Lotsch, C. Gimbert-Suriñach, "The Power of Catalytic Centers and Ascorbate in Boosting the Photocatalytic Hydrogen Evolution Performance of TpDTz 2D-COF," *Journal of the American Chemical Society* 148, no.1 (2025): 1316, <https://doi.org/10.1021/jacs.5c17806>.

24. S. Zhu, Z. Luo, F. Zhang, Y. Huang, W. Yang, Y. Xiong, R. Zhang, Z. Yu, C. Lin, X. Zeng, S. Xu, Y. Li, W. Lin, J. Chen, X. Chen, "Fluorination‐Modulated Molecular Engineering in β‐Ketoenamine Covalent Organic Frameworks toward Efficient Photocatalytic Hydrogen Evolution†," *Chinese Journal of Chemistry* 43, no.24 (2025): 3397, <https://doi.org/10.1002/cjoc.70349>.

25. C. Yang, Z. Zhang, J. Li, Y. Hou, Q. Zhang, Z. Li, H. Yue, X. Liu, "Benzotrifuran-based donor–acceptor covalent organic frameworks for enhanced photocatalytic hydrogen generation," *Green Chemistry* 26, no.5 (2024): 2605, <https://doi.org/10.1039/d3gc04972h>.

26. Z. Jia, N. Ji, J. Qi, T. Wang, Y. Che, Z. Zhao, J. Zhao, Z. Jiao, K. Wang, W. Zhang, W.-H. Zhu, "Fused-Heterocycle-Linked Covalent Organic Frameworks With Enhanced Chemical and Photochemical Stability for Photocatalysis," *Angewandte Chemie-International Edition* 64, no.35 (2025): <https://doi.org/10.1002/anie.202511245>.

27. J. Li, Z. Wang, Y. Yang, L. Zhang, J. Fan, Q. Xu, "Constructing asymmetric covalent organic frameworks to facilitate photocatalytic hydrogen production," *Chemical Communications* (2026): <https://doi.org/10.1039/D5CC07278F>.

28. X. Huang, W. Xie, T. Xu, W. Weng, T. Zhou, J. Guo, "Enantioselective Immobilization of Nonprecious Metal Complexes on Chiral Covalent Organic Frameworks for Improved Single-Site Photocatalytic Hydrogen Evolution," *Angewandte Chemie-International Edition* 64, no.30 (2025): <https://doi.org/10.1002/anie.202509095>.

29. W. Dong, Z. Qin, K. Wang, Y. Xiao, X. Liu, S. Ren, L. Li, "Isomeric Oligo(Phenylenevinylene)‐Based Covalent Organic Frameworks with Different Orientation of Imine Bonds and Distinct Photocatalytic Activities," *Angewandte Chemie International Edition* 62, no.5 (2022): <https://doi.org/10.1002/anie.202216073>.

30. R. Wang, Z. Wang, L. Li, L. Zhang, J. Zhang, H. Jin, Q. Xu, Y. Wei, Y. Yang, S. Wang, "Modulating charge transfer dynamics in one-dimensional covalent organic frameworks for boosted photocatalytic H2 generation," *Journal of Catalysis* 450, (2025): <https://doi.org/10.1016/j.jcat.2025.116289>.

31. P. Huang, Y.-Y. Peng, X.-H. Wang, R.-H. Li, M.-H. Qin, M. Zhang, S.-M. Wang, M. Lu, S.-L. Li, Y.-Q. Lan, "Charge-Distribution and Microenvironment Dual Regulation of Covalent Organic Frameworks for Enhancing Photocatalytic H2O2 and H2 Production," *Advanced Materials* (2025): <https://doi.org/10.1002/adma.202507849>.

32. J. Yang, A. Acharjya, M. Y. Ye, J. Rabeah, S. Li, Z. Kochovski, S. Youk, J. Roeser, J. Grüneberg, C. Penschke, M. Schwarze, T. Wang, Y. Lu, R. van de Krol, M. Oschatz, R. Schomäcker, P. Saalfrank, A. Thomas, "Protonated Imine‐Linked Covalent Organic Frameworks for Photocatalytic Hydrogen Evolution," *Angewandte Chemie International Edition* 60, no.36 (2021): 19797, <https://doi.org/10.1002/anie.202104870>.

33. J. Li, J. Zhou, X. H. Wang, C. Guo, R. H. Li, H. Zhuang, W. Feng, Y. Hua, Y. Q. Lan, "In situ Construction of Single‐Atom Electronic Bridge on COF to Enhance Photocatalytic H2 Production," *Angewandte Chemie International Edition* 63, no.45 (2024): <https://doi.org/10.1002/anie.202411721>.

34. X. Du, H. Ji, Y. Xu, S. Du, Z. Feng, B. Dong, R. Wang, F. Zhang, "Covalent organic framework without cocatalyst loading for efficient photocatalytic sacrificial hydrogen production from water," *Nature Communications* 16, no.1 (2025): <https://doi.org/10.1038/s41467-025-58337-w>.

35. H. Li, Z. G. Li, X. Zhang, H. Jiao, H. Wang, Z. Fan, Y. Wang, Y. Li, J. Zhang, X. H. Bu, "Construction and Microenvironment Regulation of Short Charge Transfer Tunnel at MOF/COF Heterointerfaces for Visible‐Light‐Driven Hydrogen Evolution," *Advanced Materials* (2026): <https://doi.org/10.1002/adma.202522294>.

36. J. H. Wang, A. E. Hassan, A. M. Elewa, A. F. M. El-Mahdy, "Donor-acceptor hetero 6 radialene-based three-dimensional covalent organic frameworks for organic pollutant adsorption, photocatalytic degradation, and hydrogen production activity," *Journal of Materials Chemistry A* 12, no.23 (2024): 14005, <https://doi.org/10.1039/d3ta07691a>.

37. Y. Xu, F. Qiu, Y. Fu, S.-F. Li, X. Su, K. Hong, M.-M. Zhang, X. Zhao, Y. Wang, S.-Q. Xu, "Solvent-Driven Precise Control of Stacking Configurations in Covalent Organic Frameworks for High-Efficiency Photocatalysis," *Angewandte Chemie-International Edition* 64, no.41 (2025): <https://doi.org/10.1002/anie.202512603>.

38. S. Ghosh, A. Nakada, M. A. Springer, T. Kawaguchi, K. Suzuki, H. Kaji, I. Baburin, A. Kuc, T. Heine, H. Suzuki, R. Abe, S. Seki, "Identification of Prime Factors to Maximize the Photocatalytic Hydrogen Evolution of Covalent Organic Frameworks," *Journal of the American Chemical Society* (2020): <https://doi.org/10.1021/jacs.0c02633>.

39. K. Paliusyte, L. Leao Nascimento, H. Illner, M. Wiedmaier, R. Guntermann, M. Doeblinger, T. Bein, A. O. T. Patrocinio, J. Schneider, "Surface Charge Modulation in Covalent Organic Frameworks for Controlled Pt-Photodeposition and Enhanced Photocatalytic Hydrogen Evolution," *Small* 21, no.27 (2025): <https://doi.org/10.1002/smll.202500870>.

40. L. Xiang, A. Wang, M. Shao, J. Peng, X. Song, F. Peng, L. Wang, "Cu(I)diimine-Decorated Anthracene-Containing Covalent Organic Frameworks for Photocatalytic Hydrogen Evolution," *ACS Applied Polymer Materials* 8, no.3 (2026): 1542, <https://doi.org/10.1021/acsapm.5c04208>.

41. T.-T. Sun, H. Zhang, Y. Wang, N.-N. Zhang, L.-H. Shao, H. Dong, F.-M. Zhang, "Covalent Organic Frameworks Anchoring Single-Atom Pt for Three-Phase Interface-Assisted Photocatalytic Overall Water Splitting," *Angewandte Chemie-International Edition* 64, no.46 (2025): <https://doi.org/10.1002/anie.202515397>.

42. X. Zhang, S. Fu, L. Jia, B. Hou, Y. Cui, Y. Liu, "Amplifying Chirality-Induced Spin Selectivity in Helical Covalent Organic Frameworks through Fullerene Encapsulation," *Journal of the American Chemical Society* 147, no.30 (2025): 26546, <https://doi.org/10.1021/jacs.5c06460>.

43. B. Hou, K. Wang, C. Jiang, Y. Guo, X. Zhang, Y. Liu, Y. Cui, "Homochiral Covalent Organic Frameworks with Superhelical Nanostructures Enable Efficient Chirality‐Induced Spin Selectivity," *Angewandte Chemie International Edition* 63, no.51 (2024): <https://doi.org/10.1002/anie.202412380>.

44. Z. Li, Y. Xiao, C. Jiang, B. Hou, Y. Liu, Y. Cui, "Engineering spin-dependent catalysts: chiral covalent organic frameworks with tunable electroactivity for electrochemical oxygen evolution," *National Science Review* 11, no.9 (2024): <https://doi.org/10.1093/nsr/nwae332>.

45. X. Han, C. Jiang, B. Hou, Y. Liu, Y. Cui, "Covalent Organic Frameworks with Tunable Chirality for Chiral-Induced Spin Selectivity," *Journal of the American Chemical Society* 146, no.10 (2024): 6733, <https://doi.org/10.1021/jacs.3c13032>.

46. Z. Liu, S. Zhang, E. Zhang, K. Wei, M. Zhang, X. Li, D. Fa, S. De Feyter, G. Feng, S. Lei, W. Hu, "An Insight into the Relation of Spin-Polarization and Oxygen Evolution Enhancement with a Monolayer Chiral Covalent Organic Framework Model Catalyst," *Journal of the American Chemical Society* 147, no.35 (2025): 31975, <https://doi.org/10.1021/jacs.5c09729>.
